# Orphanhood and caregiver death among children in the United States by all-cause mortality, 2000–2021

**DOI:** 10.1038/s41591-024-03343-6

**Published:** 2025-01-10

**Authors:** Andrés Villaveces, Yu Chen, Sydney Tucker, Alexandra Blenkinsop, Lucie Cluver, Lorraine Sherr, Jan L. Losby, Linden Graves, Rita Noonan, Francis Annor, Victor Kojey-Merle, Douhan Wang, Greta Massetti, Laura Rawlings, Charles A. Nelson, H. Juliette T. Unwin, Seth Flaxman, Susan Hillis, Oliver Ratmann

**Affiliations:** 1 National Center for Injury Prevention and Control, Centers for Disease Control and Prevention, Atlanta, GA, USA.; 2 Department of Mathematics, Imperial College London, London, UK.; 3 Centre for Evidence-Based Social Intervention, Department of Social Policy and Intervention, University of Oxford, Oxford, UK.; 4 Department of Psychiatry and Mental Health, University of Cape Town, Cape Town, South Africa.; 5 Institute of Global Health, University College London, London, UK.; 6 Gender Group, World Bank, Washington DC, USA.; 7 Harvard Medical School and Boston Children’s Hospital, Harvard University, Boston, MA, USA.; 8 School of Mathematics, University of Bristol, Bristol, UK.; 9 Department of Computer Science, University of Oxford, Oxford, UK.; 10 Global Reference Group for Children Affected by Crisis, University of Oxford, Oxford, UK.

## Abstract

Deaths of parents and grandparent caregivers threaten child well-being owing to losses of care, financial support, safety and family stability, but are relatively unrecognized as a public health crisis. Here we used cause-specific vital statistics death registrations in a modeling approach to estimate the full magnitude of orphanhood incidence and prevalence among US children aged 0–17 years between 2000 and 2021 by cause, child age, race and ethnicity, sex of deceased parent and state, and also accounted for grandparent caregiver loss using population survey data. In 2021, we estimate that 2.91 million children (4.2% of children) had in their lifetime experienced prevalent orphanhood and caregiver death combined, with incidence increasing by 49.5% and prevalence by 7.9% since 2000. Populations disproportionately affected by orphanhood included 5.2% of all adolescents; 6.4% and 4.7%, respectively, of non-Hispanic American Indian or Alaska Native, and non-Hispanic Black children; and children in southern and eastern states. In 2021, drug overdose was the leading cause of orphanhood among non-Hispanic white children, but not among minoritized subgroups. Effective policies and programs to support nearly three million bereaved children are needed to reduce the acute and long-term negative effects of orphanhood.

Children’s lives are increasingly impacted by intersecting crises, including pandemics, organized crime, political and social conflict, and climate change. One of the most fundamental threats that children are facing through escalating crises is orphanhood, defined by United Nations Children’s Fund as the death of one or both parents^1,2^, and more generally caregiver loss, including the death of co-residing grandparent caregivers who are responsible for most or some needs of their grandchildren^3,4^. Nearly 15 years ago, the World Health Organization (WHO) identified parental death as an adverse childhood experience increasing adult mental health risks^4^. As an adverse childhood experience, parent or caregiver loss may have lifelong consequences^5–8^, including increased risks of suicide, post-traumatic stress disorder, violence, insecure housing^9^, and chronic and infectious diseases^10,11^. These consequences often lead to ongoing needs for health and mental health services; parenting, educational and economic support for affected children remaining with surviving parents or caregivers; and foster care or adoption services for children bereft of care^11^. Yet, available data on caregiver loss are limited to specific causes such as human immunodeficiency virus (HIV) and acquired immunodeficiency syndrome (AIDS)^12^, coronavirus disease (COVID-19)^13^, maternal cancers^14^ or drug overdose^15,16^.

Despite serious risks for bereaved children, we know that timely, multifactored policies that address social determinants of health and promote safe, stable and nurturing relationships and environments are effective in restoring hope and building resilience for orphaned and vulnerable children, their families and communities^17,18^. For example, during the COVID-19 pandemic, real-time COVID-19-associated caregiver-loss incidence estimates^19–21^ led to policies that support bereaved children and families including recommendations in the US National COVID-19 Pandemic Preparedness Plan^22^; investments in financial, bereavement and mental health support in some states^23^; and a range of global financial, legal and educational support across Brazil, Colombia, Peru, Indonesia and Mexico City^24^. The World Bank Rapid Social Response Program dedicated funding supporting 12 nations addressing pandemic-linked orphanhood and caregiver-loss agendas^25^. Furthermore, since 2003, the multibillion dollar President’s Emergency Plan for AIDS Relief has been investing 10% of bilateral funding to provide nutritional, educational, psychosocial and livelihood support for 7.2 million children who were orphaned and vulnerable due to the HIV pandemic^26^. Together, these responses and frameworks^27^ raise the possibility that new standards of care can be extended for any child experiencing orphanhood or grandparent caregiver loss, regardless of cause^14^.

In the United States, the COVID-19 pandemic intersected with increasing challenges including substance use, economic crises and mental health distress, resulting in compounding deaths due to overdose, suicide, excessive alcohol use and contagion^28,29^. Caregiver loss amidst intersecting social and health crises is profoundly exacerbated by inequities in access to social determinants of health, including financial support, education, housing, employment and access to health care. These inequities intensify child risks and vulnerability in particular populations, such as non-Hispanic American Indian or Alaska Native children^20^.

Understanding the full extent of orphanhood and grandparent caregiver loss due to all causes in the United States, including incidence, prevalence trends and associated inequalities, is essential for informing evidence-based prevention and response strategies for affected children and families. In particular, relative to white children, non-Hispanic Black, non-Hispanic Asian and Hispanic children are twice as likely to live with a grandparent^20^. From a policy perspective, estimates of orphanhood and grandparent-caregiver-loss prevalence are essential for informing the extent of essential investments needed for children under age 18, as the psychosocial, economic, housing and educational consequences of such losses continue to threaten well-being, health and mental health throughout childhood and adolescence. Just as vital statistics and death records inform public health prevention and response policies according to leading causes of death^30^, this approach may widely inform support systems for children experiencing caregiver loss.

In this study, we have three aims. First, we leverage standard vital statistics records to establish a modeling approach for comprehensively estimating prevalence, incidence and trends in all-cause orphanhood and co-residing grandparent caregiver loss. Second, we aim to estimate the numbers, rates, time trends and disparities in all-cause caregiver loss among children in the United States between 2000 and 2021, accounting for orphanhood and co-residing grandparent caregiver loss among the ~6.6 million (9.1%) US children who live with a grandparent who owned or rented their housing and provided some or all of their basic needs^31,32^. Third, we aim to characterize the leading and evolving drivers of orphanhood in terms of causes of parental death among children in the United States between 2000 and 2021. We characterize leading causes of orphanhood and identify the extent to which the compounding crises of the COVID-19 pandemic and drug overdose epidemic in 2020 and 2021 were associated with escalating orphanhood. We aim to identify populations disproportionately affected, by child age, race and ethnicity, orphanhood type (maternal or paternal) and state, to advance evidence-based strategies responsive to social determinants of health. Our findings may strengthen or expand policies and programs that address parental and caregiver loss and its consequences (Table 1).

## Results

### Estimating all-cause orphanhood from vital statistics data

**Extended Data Fig. 1 |:**
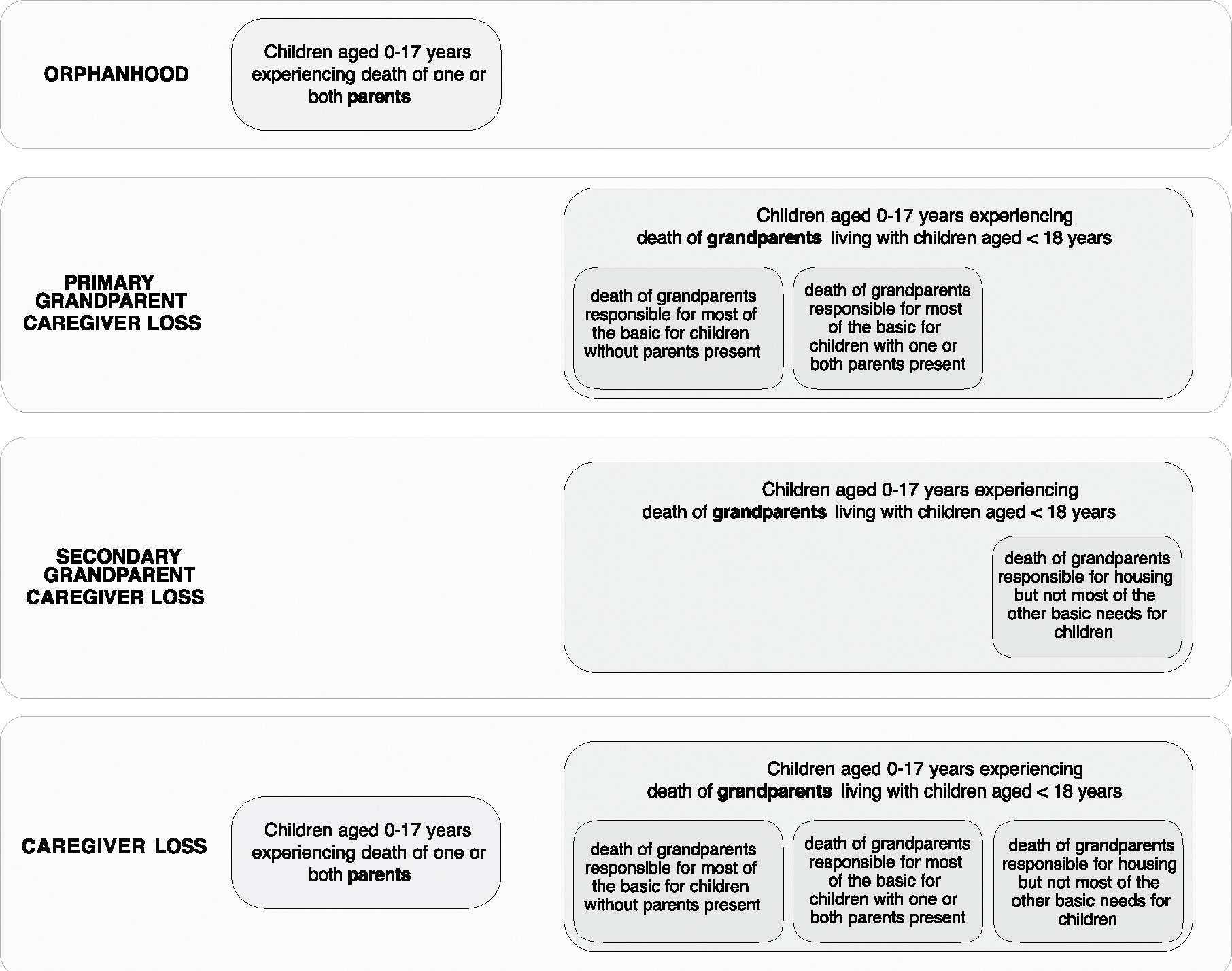
Definition of orphanhood and grandparent caregiver loss. To quantify caregiver loss from all causes, we considered orphanhood defined as children experiencing the death of one or both parents, as well as in^20^ and following American Community Cohort definitions^61–63^ the loss of primary grandparent caregivers aged 30 years or older who live in the same household and provide care in the absence of a parent, or who provide for most of the basic needs for children, and separately the loss of secondary grandparent caregivers aged 30 years or older serving as head of household who own or rent the family’s housing and provide for some but not most of the basic needs of their grandchildren^32,63^.

**Extended Data Fig. 2 |:**
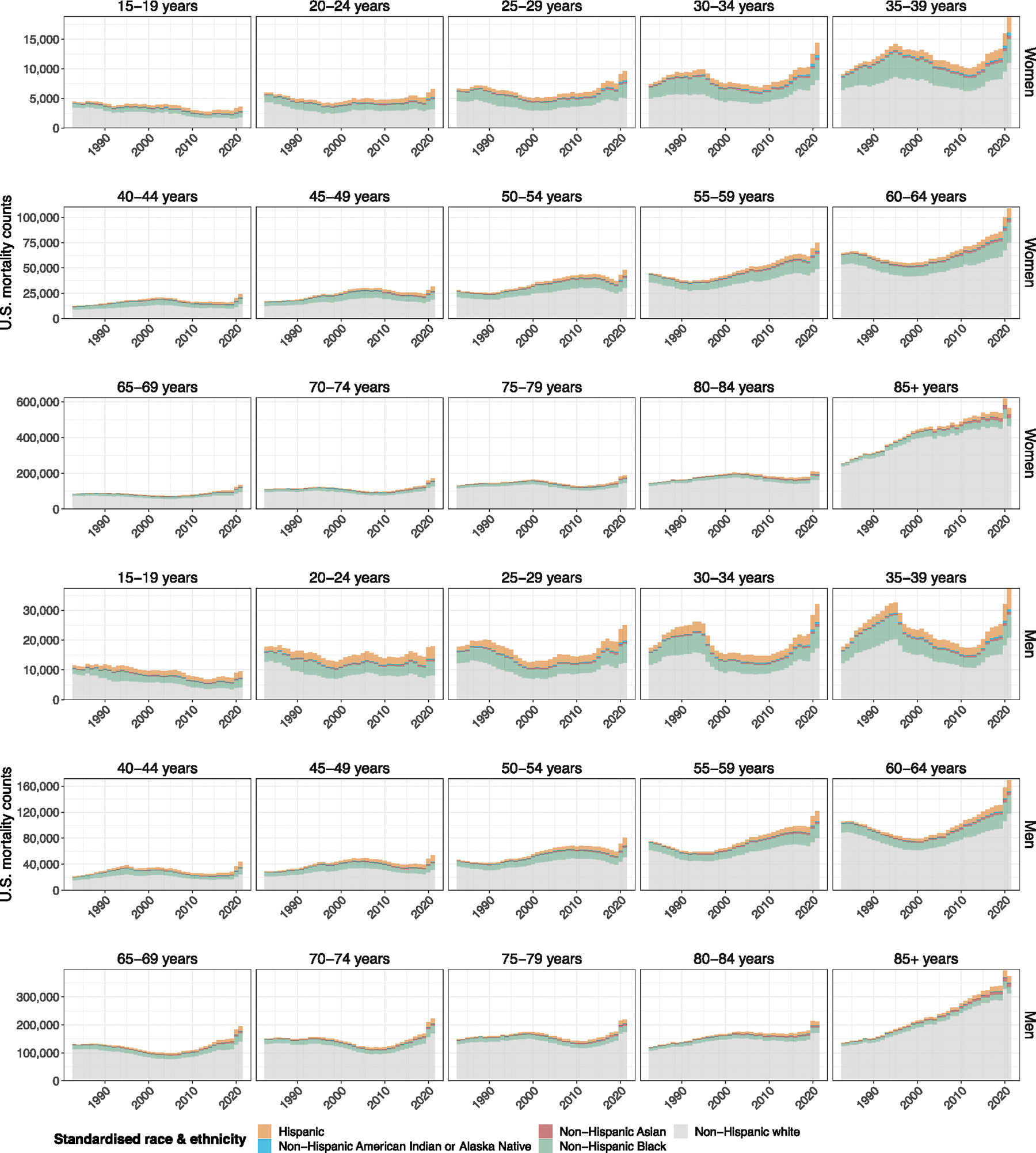
Aggregated age- and sex-specific U.S. mortality counts in 1983–2021, by standardized race and ethnicity. Line-list mortality data on US residents were retrieved from the NCHS Vital Statistics portal for each year in 1983–2021 (https://www.cdc.gov/nchs/data_access/vitalstatsonline.htm) for orphanhood and grandparent caregiver loss estimation. Data were collected from 1983 onwards because the corresponding children who lost a caregiver in 1983 at age 0 were of age 17 in 2000, and so entered our estimation of orphanhood prevalence in 2000. Individuals of other races in 1984–1991 and individuals of more than one race in 2021 were not coded consistently and not included in this study (see Methods). In total, n=93,921,920 death records were included in national orphanhood and grandparent caregiver loss estimation.

**Extended Data Fig. 3 |:**
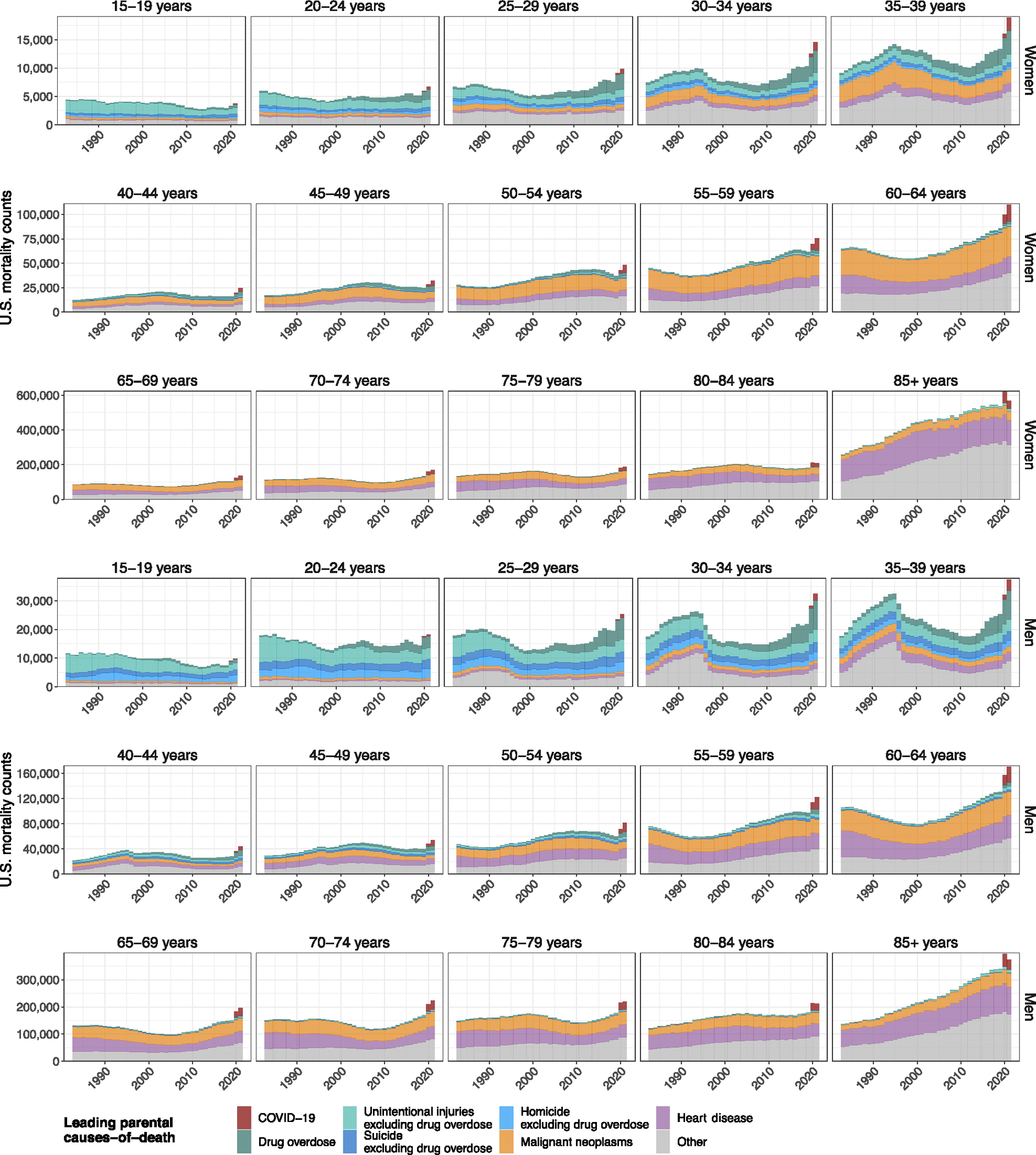
Aggregated age- and sex-specific US mortality counts in 1983–2021, by leading parental causes of death. Line-list mortality data on US residents were retrieved from the NCHS Vital Statistics portal for each year in 1983–2021 (https://www.cdc.gov/nchs/data_access/vitalstatsonline.htm) for orphanhood and grandparent caregiver loss estimation. Causes of death were reported in the Ninth or Tenth Revision of the International Classification of Disease, which we mapped into one of 53 caregiver loss causes-of-death categories (or ‘Other’ category, Supplementary Table 1), and analysed in terms of the leading parental causes of death in 2021 (Methods). In total, n=93,921,920 death records were included in national orphanhood and grandparent caregiver loss estimation.

**Extended Data Fig. 4 |:**
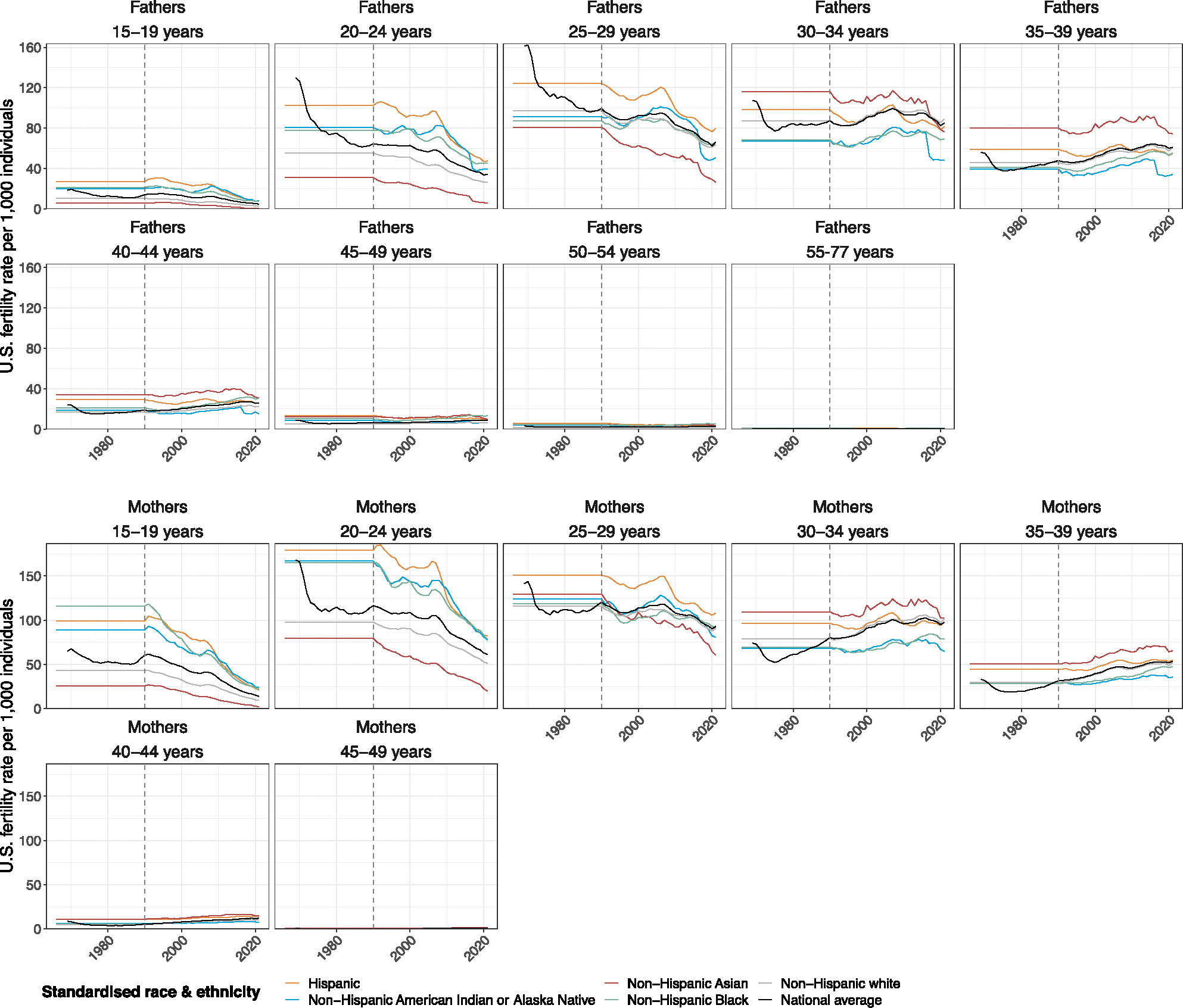
Age- and sex-specific fertility rates, by standardised race & ethnicity. Line-list live births data for US mothers aged 15–49 years and corresponding fathers aged 15–77 years were retrieved from the NCHS Vital Statistics portal for each year between 1969 to 2021 (https://www.cdc.gov/nchs/data_access/vitalstatsonline.htm) for orphanhood estimation. Standardized race and ethnicity categories were attributed as described in Supplementary Table 7 (colour). Prior to 1990, we assumed the same fertility rates in each year in 1966–1989 as in 1990. For comparison, the black lines show national-level fertility rates, which we calculated directly in 1969–1989, and as weighted average of the standardized race and ethnicity-specific fertility rates in 1990–2021. The dashed vertical line represents 1990. In total, n=127,463,725 live birth records from 1990 were included in national orphanhood estimates.

We developed a broadly applicable approach for inferring incidence and prevalence of orphanhood among children aged 0–17 years from any cause of parental death based on widely reported age- and sex-specific vital statistics on individual live birth and death registrations, along with population size estimates (Extended Data Fig. 1). Our approach, adapted from a previous study^20^, centers on attributing to each deceased woman aged 15–66 years and each deceased man aged 15–94 years the average number of children orphaned using subgroup-specific fertility rates in the previous 0–17 years (Methods). Line-list vital statistics released by the National Center for Health Statistics (NCHS)^33^ in the United States (Extended Data Figs. 2–4) are linked to information on race and ethnicity of decedents and causes of death reported in the ninth or tenth revision of the International Classification of Diseases (ICD-10), which we mapped into one of 53 rankable caregiver-loss cause-of-death categories (or ‘other’ category; Supplementary Table 1). This enables the estimation of orphanhood stratified by age of child, and by age, sex, race and ethnicity, and cause of death of parent, based on population-level administrative registration data rather than sampling-based survey data (Methods). Incidence calculations adjust for double counting in case the opposite-sex parent died previously, and prevalence of orphanhood is calculated by cumulatively summing incidence estimates by 1 year age of child in the current and previous 17 years, excluding children who would have since turned 18. For the United States, we further extended orphanhood estimates to include loss of co-residing primary (both grandparents providing care in the absence of parents and primary grandparent caregivers providing most of their grandchildren’s basic needs) and secondary grandparent caregivers serving as head of household who own or rent the family’s housing based on data from the US Census American Community Survey (ACS)^34^. To avoid double counting of children affected by loss of both a parent and grandparent caregiver, all caregiver-loss totals were de-duplicated (Methods).

### Trends in US orphanhood and grandparent caregiver loss

**Extended Data Fig. 5 |:**
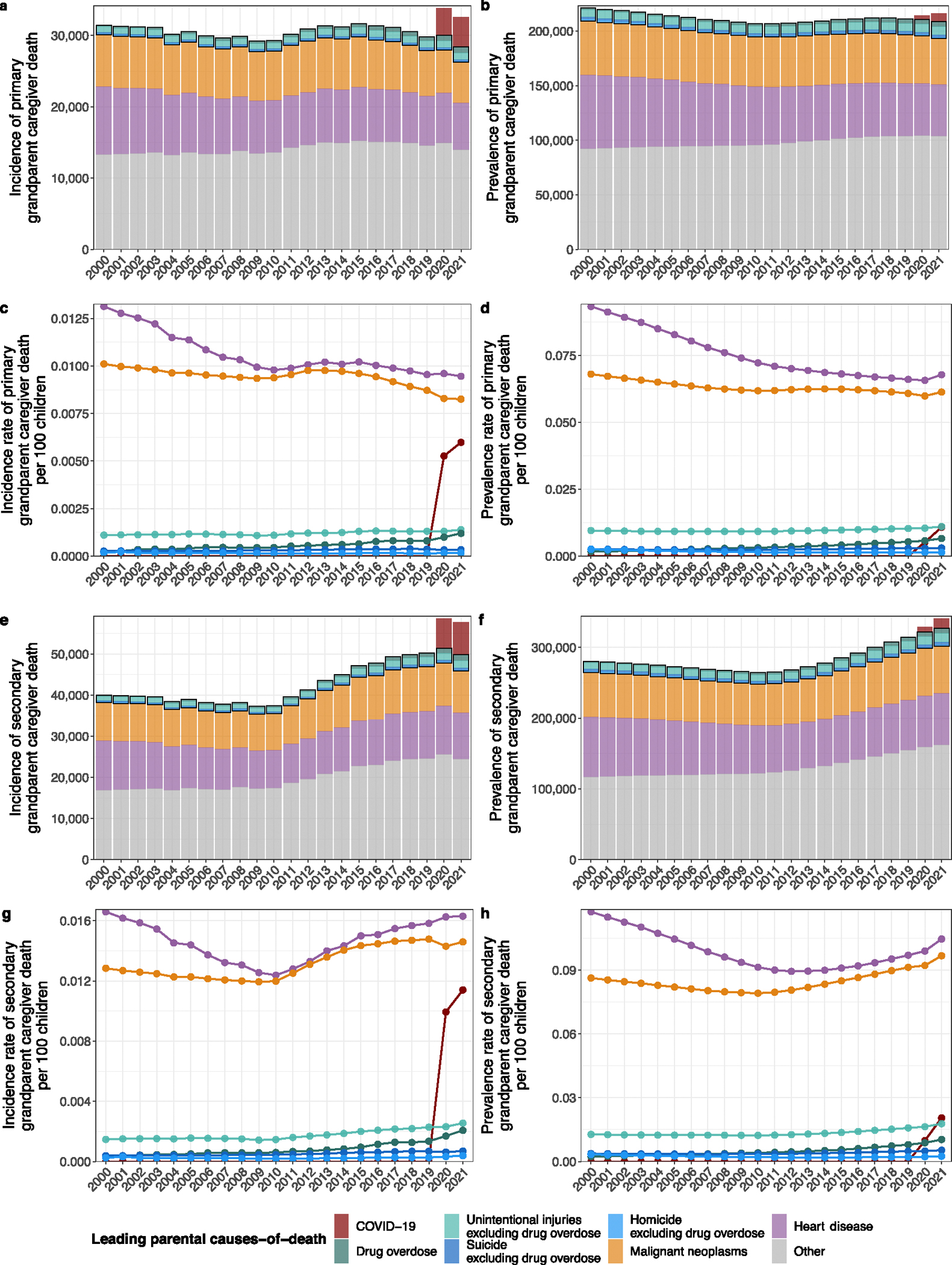
Magnitude of children experiencing primary and secondary grandparent caregiver loss in the US. (**a**) Estimated number of US children newly experiencing primary grandparent caregiver death (incidence) by leading parental causes of death (colour). (**b**) Estimated number of US children experiencing primary grandparent caregiver death in their lifetime (prevalence) by leading parental causes of death (colour). (**c**–**d**) Incidence and prevalence rates of primary grandparent caregiver death by leading parental causes of death among US children. Median estimates are shown (points) with uncertainty ranges in Supplementary Table 18. (**e**–**h**) Corresponding figures for secondary grandparent caregiver loss. Median estimates are shown (points) with uncertainty ranges in Supplementary Table 19. 2021 incidence estimates are based on an average of n=5,791 sex-, race and ethnicity- and cause-specific mortality records, and an average of n=610,229 race and ethnicity-specific natality records.

In 2021, we estimate that 494,036 (95% uncertainty interval (UI): 457,957–533,274) children experienced incident orphanhood or grandparent caregiver death from any cause in the United States corresponding to 0.71% of children (Table 2). Most children (82.5%) lost a parent, while 6.6% lost a primary grandparent caregiver providing care in the absence of parents or providing most of their grandchildren’s basic needs and 11.7% a secondary grandparent caregiver providing housing but not most other basic needs (Extended Data Fig. 5). Incidence of orphanhood and grandparent caregiver death combined increased from 2000 to 2019 by 11.7%, and then rapidly through the COVID-19 pandemic years 2020–2021 by 33.9% (Table 2). Prevalence of orphanhood and grandparent caregiver loss combined decreased slightly by 1.3% from 2000 to 2019, then increased by 9.4% during the pandemic years 2020–2021. In 2021, we estimate that 2,912,817 (95% UI: 2,654,936–3,202,040) children experienced prevalent orphanhood or caregiver death, representing 4.2% of all children, of whom 81.6% lost a parent, 7.5% a primary grandparent caregiver and 11.7% a secondary grandparent caregiver in their lifetime. These estimates are an order of magnitude higher than previous cause-specific reports describing children orphaned by HIV or AIDS (1998)^35^, maternal cancers (2020)^14^, COVID-19 (2020–2022)^13^, drug overdose (2011–2021)^15^ and firearms^16^.

### Leading causes of orphanhood

Increases in caregiver-loss incidence between 2000 and 2021 were highest among children losing one or both parents (~56%; Table 2). We next focused on characterizing the drivers and trends in orphanhood in terms of the leading parental causes of death as of 2021 (Extended Data Fig. 1 and Supplementary Table 2): COVID-19, drug overdose, the remaining top five causes of death overall (heart disease, cancer, unintentional injuries, cerebrovascular diseases, chronic lung disease) and any additional causes of death in the top five for men and women aged 15–44 (suicide and homicide), as these ages include those more likely to be parents. We used a hierarchical approach for classifying cause of death; therefore, we singled out drug overdose from the three categories suicide, homicide and unintentional injuries (for example, motor vehicle crashes) (Methods and Supplementary Table 2).

**Fig. 1 |:**
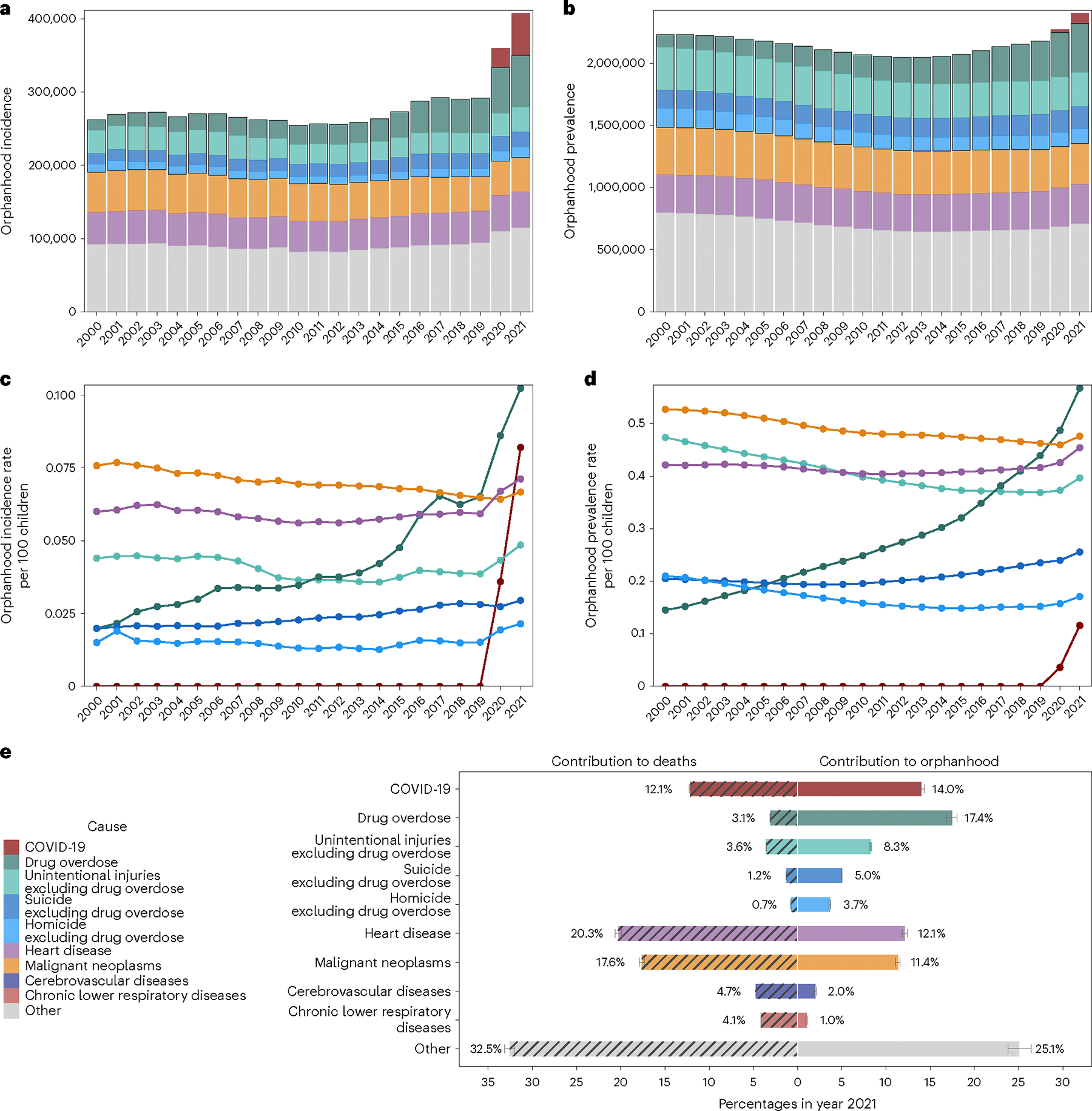
Magnitude of children experiencing orphanhood in the United States. **a**, Estimated number of US children newly experiencing orphanhood by any cause and over time. **b**, Estimated number of US children experiencing orphanhood in their lifetime by any cause and over time. **c**, Incidence rates of orphanhood among US children. **d**, Prevalence rates of orphanhood among US children, calculated by aggregating incidence estimates among children over the previous 17 years and accounting for the aging of children. **e**, Main contributors to orphanhood incidence versus to adult deaths in 2021. Throughout, median estimates are shown (points, bars and values shown in text), and uncertainty ranges are detailed in Supplementary Table 17, except for **e** in which 95% UIs are also shown as error bars. Incidence estimates are based on at least *n* = 2,357,714 mortality records and *n* = 3,610,887 natality records per year. Cause-specific incidence estimates are based on at least *n* = 14,854 mortality records per year.

Since 2020, drug overdose has been the leading cause of orphanhood incidence and prevalence, surpassing COVID-19 (Fig. 1). In 2000, we estimate that only 0.02% of children experienced orphanhood caused by parental drug overdose, increasing to 0.04% in 2012 and then sharply to 0.07% in 2019 and 0.10% in 2021.

More broadly, the incidence and prevalence of orphanhood due to fatal injuries—comprising drug overdose, suicide, homicide and unintentional injuries—exceeded orphanhood due to leading chronic disease (heart disease and cancers) causes of death in parents. Orphanhood due to parental suicide decreased until 2008 and subsequently increased; orphanhood due to parental homicide and unintentional injuries decreased until 2019, then increased until 2021. With the coronavirus pandemic, orphanhood due to COVID-19 emerged in 2020. In comparison, orphanhood due to parental death from malignant neoplasms consistently decreased until 2000; orphanhood due to death from cardiovascular diseases remained relatively unchanged until 2019, then increased in the COVID-19 pandemic. The disproportionate impact of parental fatal injuries on orphanhood was evident in 2021, with drug overdose contributing 17.4% to orphanhood incidence, yet only 3.1% to adult mortality (Fig. 1 and Supplementary Table 3); similarly, unintentional injuries, suicide and homicide in parents contributed 8.3%, 5.0% and 3.7% to orphanhood, versus 3.6%, 1.2% and 0.7%, respectively, to adult deaths. We provide data by maternal and paternal orphanhood, co-residing grandparent caregiver loss and all caregiver loss causes of death in Supplementary Tables 4 and 5.

### Primary factors linked with variations in orphanhood prevalence

**Fig. 2 |:**
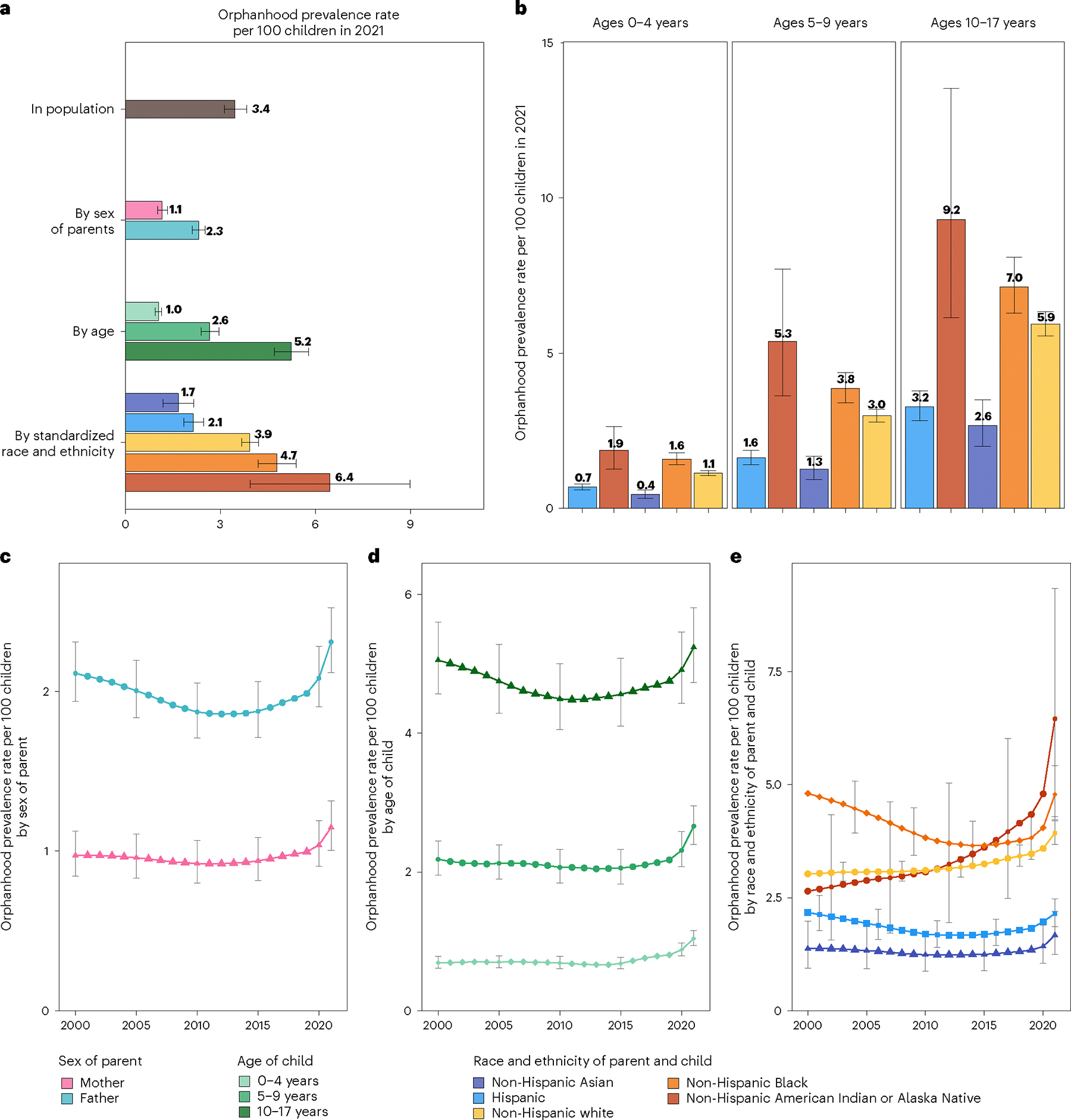
Differences and time trends in orphanhood among US children by age, sex, standardized race and ethnicity, and cause. **a**, Orphanhood prevalence rates of all-cause orphanhood by sex of parent, age of child and standardized race and ethnicity of child and parent in 2021. **b**, Orphanhood prevalence rates by race and ethnicity in each age group among US children. **c**–**e**, Time trends in prevalence rates of maternal orphanhood and parental orphanhood (**c**), age of child (**d**), and race and ethnicity of child and parent (**e**). Throughout, median estimates (points, bars and values shown in text) are shown along with 95% UIs (error bars). Estimates are based on at least *n* = 148,850 group-specific mortality records and *n* = 642,737 group-specific natality records per year.

We expected extensive heterogeneity in orphanhood burden by age and race and ethnicity of child and sex of parent as seen during the COVID-19 pandemic^20^, and differences in the drivers of orphanhood along these strata especially due to different causes of death by sex and race and ethnicity in parent age ranges (Extended Data Figs. 2–4). The race and Hispanic origin of decedents were typically reported by the next of kin and recorded in varying formats over time, which we standardized to five race and ethnicity categories (Supplementary Tables 6–8), and we assumed that the race and ethnicity of the child matched those of the parent. Decedents of more than one race were not coded consistently across the time period required for orphanhood estimation and excluded in this study, corresponding in 2021 to 0.47% of deaths. In 2021, 1,691,918 children aged 10–17 years (5.2% of children) experienced prevalent orphanhood, 5.0 times more likely than children aged 0–4 years (Fig. 2 and Supplementary Table 9). Among the orphaned children, 66.8% lost their father in their lifetime and 33.2% lost their mother (Supplementary Table 10). Disparities were even larger across race and ethnicity. In 2021, orphanhood affected 6.4% of non-Hispanic American Indian or Alaska Native children, 4.7% of non-Hispanic Black children, 3.9% of non-Hispanic white children, 2.1% of Hispanic children and 1.7% of non-Hispanic Asian children (Supplementary Table 11), with additional heterogeneity by age of child (Fig. 2).

### Impact of drug overdose on orphanhood

**Extended Data Fig. 6 |:**
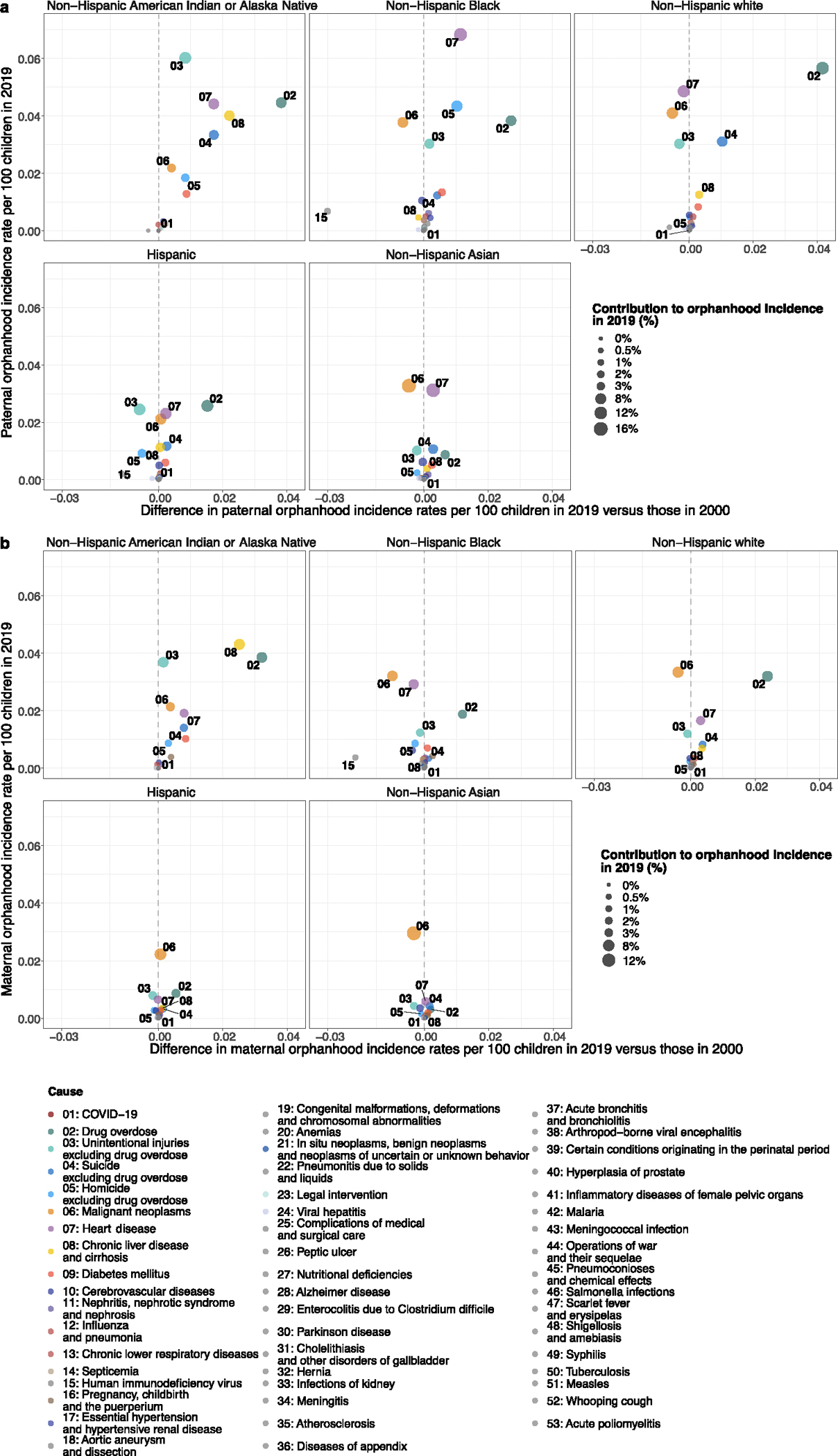
Leading causes of orphanhood incidence among US children in 2019 by race and ethnicity and sex of parent. (**a**) Estimates of 2019 paternal orphanhood incidence (y-axis) by cause-of-death of fathers (point, number, colour) and race and ethnicity (panels) versus differences in incidence rates in 2019 minus those in 2000 (x-axis), with positive differences indicating increasing incidence rates and negative differences indicating decreasing incidence rates. The size of points indicates the contribution of each cause to new cases of orphanhood among US children in 2019. (**b**) The same for 2019 maternal orphanhood incidence by cause-of-death of mothers. Median estimates are shown (points) and uncertainty ranges are detailed in Supplementary Table 13. 2019 incidence estimates are based on an average of n=5,644 sex-, race and ethnicity- and cause-specific mortality records, and an average of n=748,936 race and ethnicity-specific natality records.

**Fig. 3 |:**
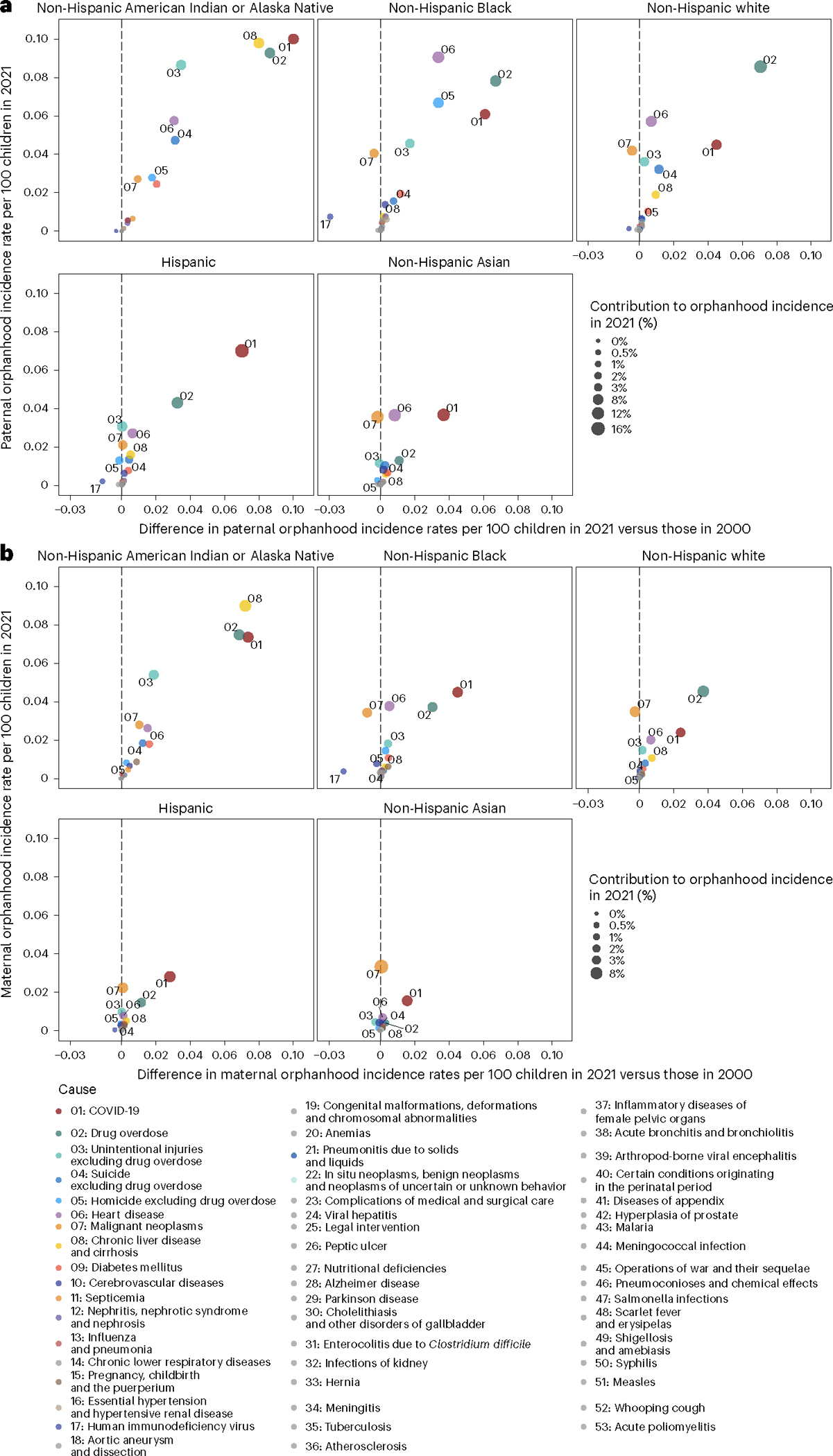
Leading causes of orphanhood incidence among US children in 2021 by race and ethnicity and sex of parent. **a**, Estimates of 2021 paternal orphanhood incidence (*y* axis) by cause of death of fathers (point, number, color) and race and ethnicity (panels) versus differences in incidence rates in 2021 minus those in 2000 (*x* axis), with positive differences indicating increasing incidence rates and negative differences indicating decreasing incidence rates. The size of the points indicates the contribution of each cause to new cases of orphanhood among US children in 2021. **b**, The same for 2021 maternal orphanhood incidence by cause of death of mothers. Median estimates are shown (points) and uncertainty ranges are detailed in Supplementary Table 12. The 2021 incidence estimates are based on an average of *n* = 5,791 sex-, race and ethnicity- and cause-specific mortality records, and an average of *n* = 610,229 race and ethnicity-specific natality records.

The causes underpinning these disparities in orphanhood differed primarily by parental race and ethnicity and sex (Fig. 3, Extended Data Fig. 6 and Supplementary Tables 12 and 13). Although drug overdose was among the top three causes of both maternal and paternal orphanhood for almost all race and ethnicity groups (except Asian mothers and fathers), we found that causes other than drug overdose were the leading cause of orphanhood in every minoritized subgroup. The top cause of orphanhood was COVID-19 in fathers and chronic liver disease in mothers of non-Hispanic American Indian or Alaska Native children, heart disease in fathers and COVID-19 in mothers of non-Hispanic Black children, COVID-19 in fathers and mothers of Hispanic children, and heart disease and COVID-19 (tied) in fathers and cancers in mothers of non-Hispanic Asian children. This suggests that new standards of care and services need to be contextualized for each of the most vulnerable child populations. Our time trend analyses further showed that, except for cancers in mothers of non-Hispanic Asian children, orphanhood incidence increased substantively in all leading causes since 2000 (Fig. 3 and Extended Data Fig. 6).

### Extent of orphanhood across US states

**Extended Data Fig. 7 |:**
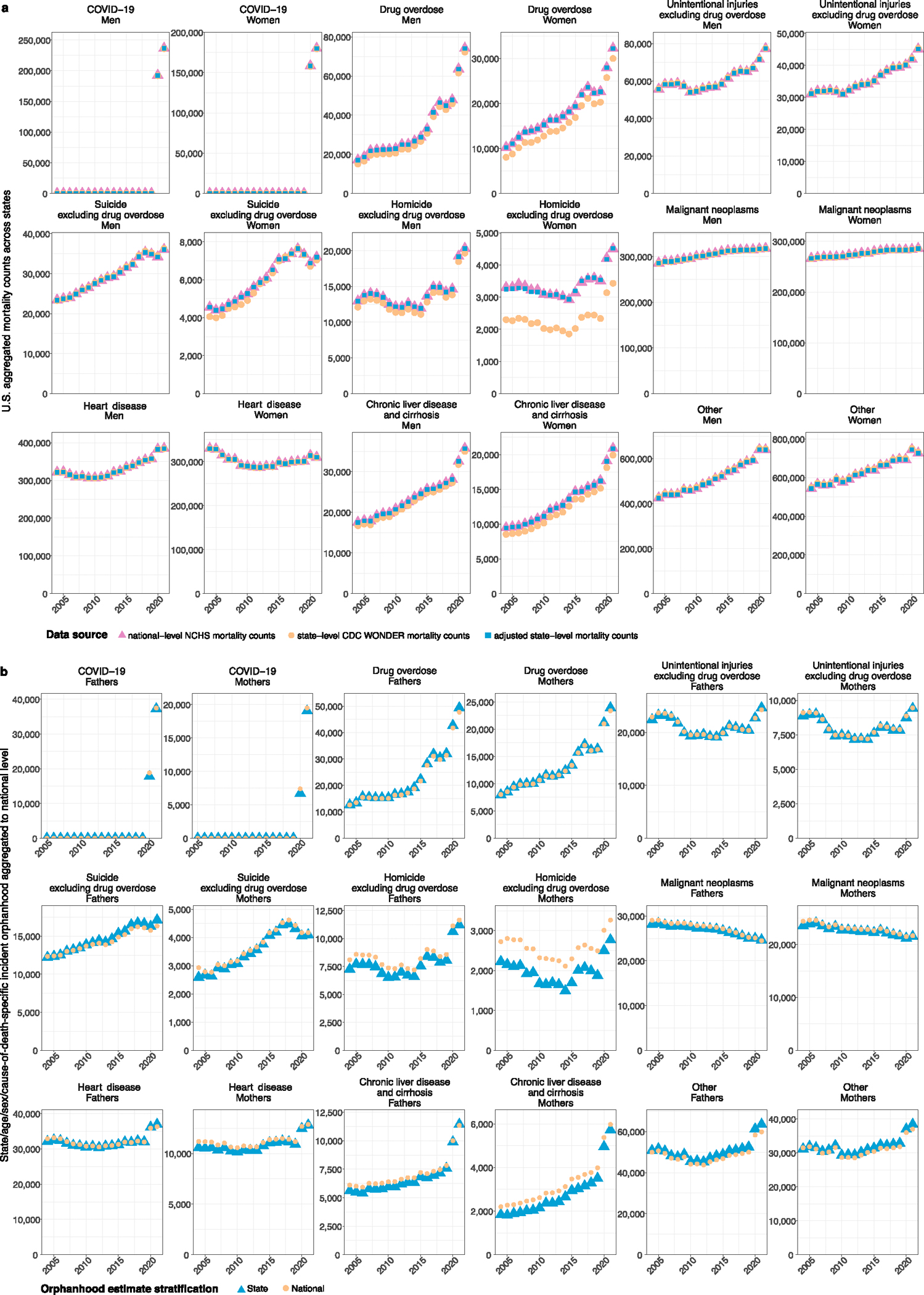
Reliability evaluation of orphanhood incidence estimates by state and leading parental cause of death. (**a**) Comparison of state-, sex-, age- and leading causes-specific mortality counts for 2005–2021 that were extracted from CDC WONDER to the corresponding suppression-adjusted counts, and to the national-level sex-, age- and leading parental causes-specific counts obtained from NCHS (see Methods). (**b**) Comparison of state-level orphanhood incidence estimates to national-level estimates by sex and leading parental causes of death for 2005–2021 (see colour, symbols). Incidence estimates were very similar except for ‘Homicide excluding drug overdose’ in women, ‘Chronic liver disease and cirrhosis’ in women and ‘Other’ causes of death (see Methods). Only median estimates are shown.

**Extended Data Fig. 8 |:**
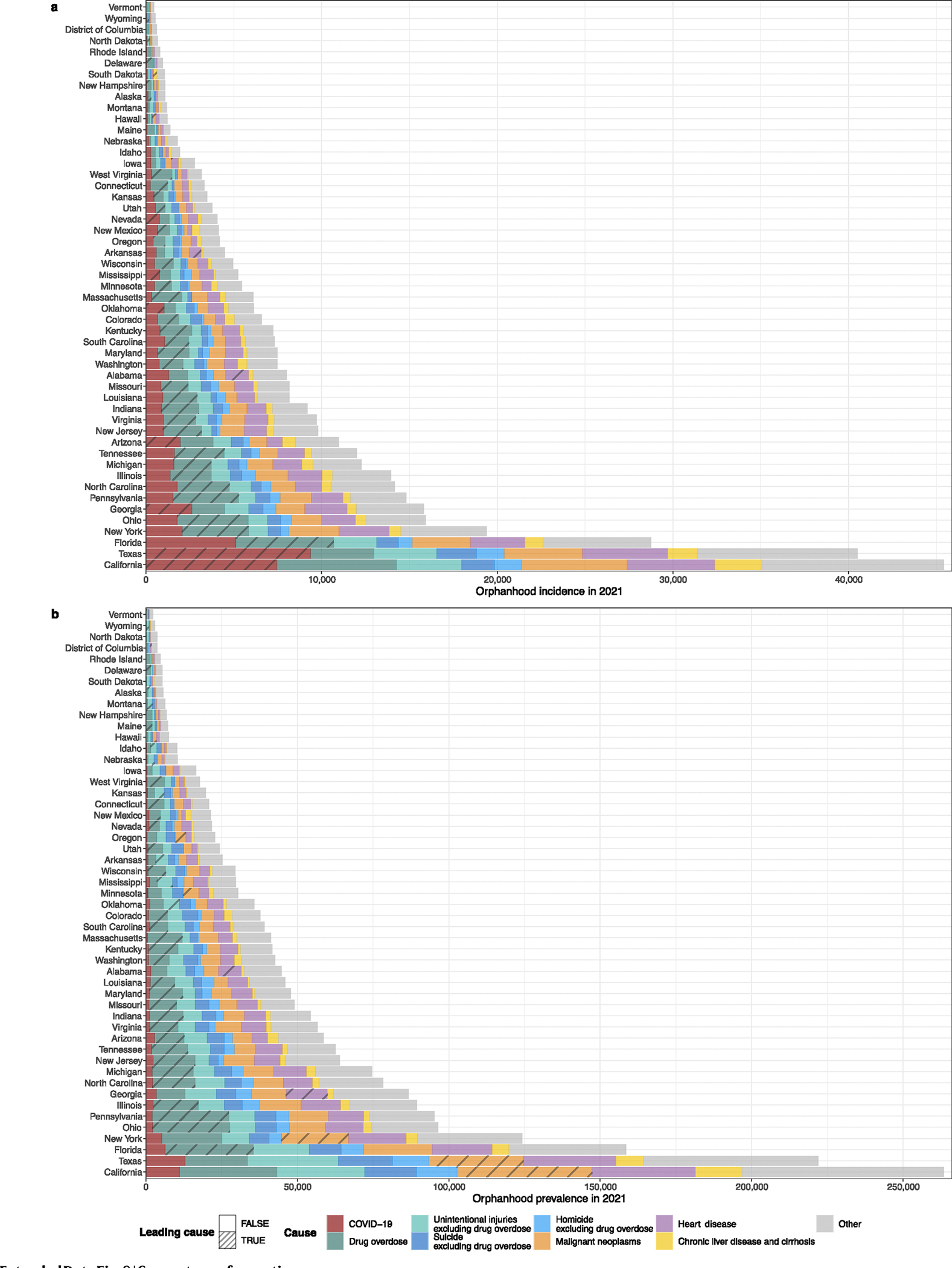
Leading parental causes of orphanhood incidence and prevalence in 2021 by US state. (**a**) Estimated number of US children newly experiencing orphanhood, by state and leading parental causes of death. (**b**) Estimated number of US children experiencing orphanhood in their lifetime (over ages 0–17 years), by state and leading parental causes of death. Throughout, the leading parental cause-of-death in each state is indicated with hatch marks. Throughout, median estimates (bars) are shown and uncertainty intervals are provided in Supplementary Table 14. Incidence estimates for 2021 are based on an average of n=5,071 state- and cause-specific mortality records and n=71,791 state-specific natality records, and prevalence estimates are based on aggregating incidence estimates among children over the previous 17 years while accounting for ageing.

**Extended Data Fig. 9 |:**
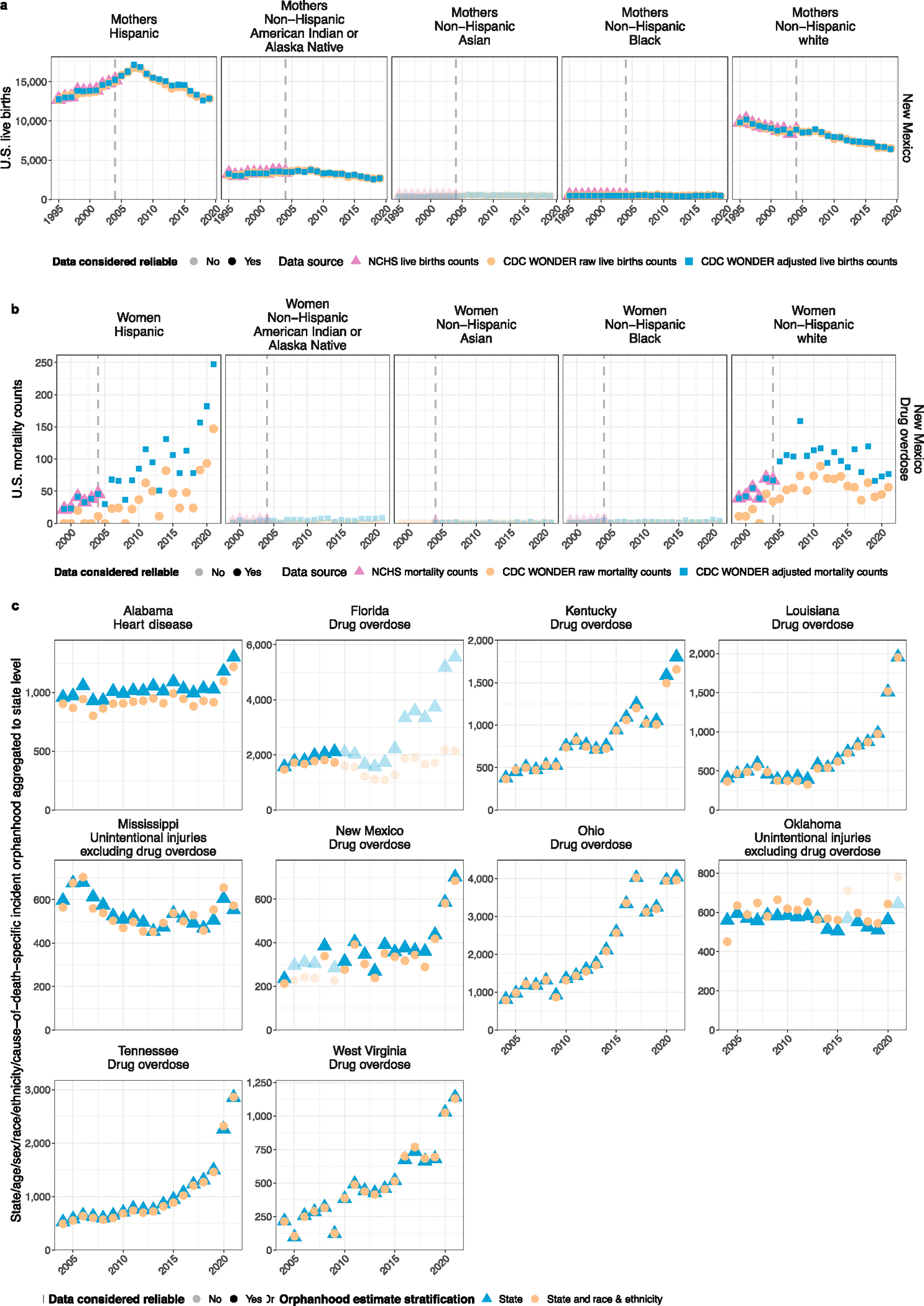
Reliability evaluation of orphanhood incidence estimates by state, race and ethnicity and leading parental cause-of-death. (**a**) Example of completeness evaluation for state-, sex-, age- and race and ethnicity-specific live birth counts in New Mexico (see Methods). (**b**) Example of completeness evaluation for state-, sex-, age-, race and ethnicity- and leading-causes-specific mortality counts in New Mexico (see Methods). Only data shown in bold were retained for further analysis. (**c**) To assess the reliability of state- and race and ethnicity-specific orphanhood estimates, we considered weighted averages of the orphanhood incidence estimates across race and ethnicity, and then compared the weighted averages against state-level estimates (see colour, symbols). Triangles and circles are shown in opaque colour when race and ethnicity-specific estimates disagreed by more than 20% and were considered unreliable. Only median estimates are shown.

**Fig. 4 |:**
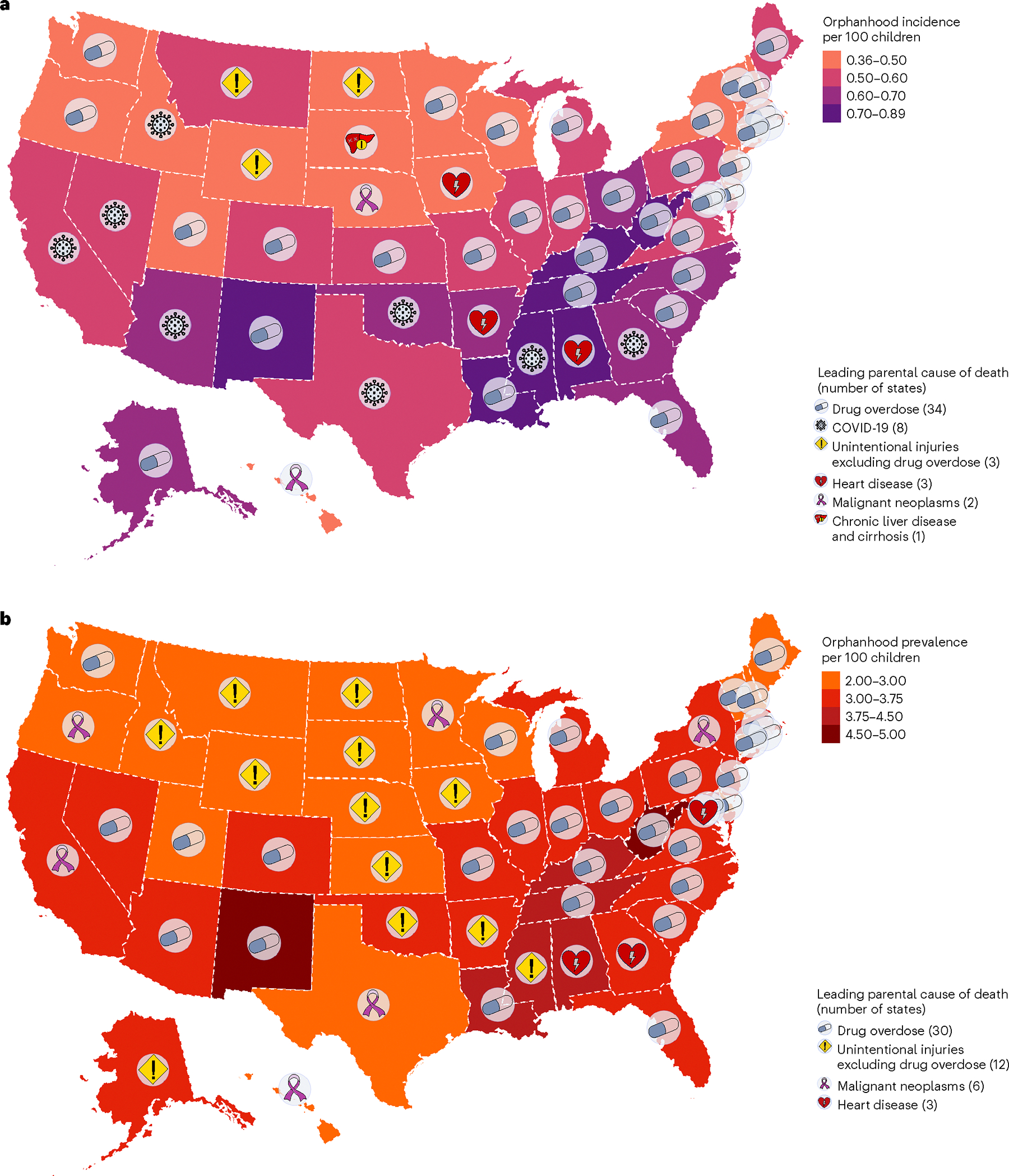
Spatial distribution of US children experiencing orphanhood in 2021. **a**, Map of orphanhood incidence rates per 100 children (color) by state and leading cause (symbol). **b**, Map of orphanhood prevalence rates per 100 children (color) by state and leading cause (symbol). Median estimates are shown (colors), and uncertainty ranges are detailed in Supplementary Table 14. Incidence estimates for 2021 are based on an average of *n* = 5,071 state- and cause-specific mortality records and *n* = 71,792 state-specific natality records, and prevalence estimates are based on aggregating incidence estimates among children over the previous 17 years while accounting for aging. Basemaps from Mapbox and OpenStreetMap were generated by ‘Leaflet’ package version 2.2.0 in R under a Creative Commons license CC BY-SA 2.0.

To provide a reference for policy development at the state level, we next generated state-level estimates of cause-specific incidence and prevalence of orphanhood and caregiver death for 2021. NCHS live births and mortality data did not include information on state of residence from 2005 onward, and we used live births and mortality data stratified by 5 year age bands, sex and state from the Centers for Disease Control and Prevention (CDC) WONDER (https://wonder.cdc.gov/) with counts below 10 suppressed^12,36^, which we accounted for using imputation and correction factors (Methods and Extended Data Fig. 7). In 2021, we estimate that 30 states had >3% of all children experiencing prevalent orphanhood, pervasively covering almost all regions of the United States (Fig. 4 and Supplementary Table 5). California, Texas and Florida had the highest orphanhood incidence and prevalence in 2021. West Virginia and New Mexico had the highest incidence (0.8–0.9%) and prevalence rates (4.5–5.0%) of orphanhood in 2021. Injury-associated parental deaths—including overdose, suicide and/or unintentional injury—were among the top two causes of orphanhood prevalence in 47 states (Extended Data Fig. 8 and Supplementary Table 14). Drug overdose was the leading cause of orphanhood prevalence in 30 states (Fig. 4), with high rates of orphanhood prevalence due to overdose in white children and also minoritized subgroups, where further evaluation by state and race and ethnicity was possible (Extended Data Fig. 9 and Supplementary Table 15).

## Discussion

We estimate that in 2021 over 2.91 million children—4.2% of all children in the US—had lost a parent or a primary or secondary grandparent caregiver in their lifetime. The lives of these children are permanently affected by the loss of their fathers, mothers and co-residing grandparents who provided their homes, needs and care^4,20,31,37^ (Table 2). Populations disproportionately impacted by all-cause orphanhood in 2021 included over 1.69 million adolescents aged 10–17 years (1 of every 20 adolescents) and children of non-Hispanic American Indian or Alaska Native, and non-Hispanic Black, race and ethnicities (approximately 1 of 15 and 1 of 20 children, respectively). We observed the highest orphanhood burden among non-Hispanic American Indian or Alaska Native adolescents—approximately 1 of 10 children—on par with the 9% orphanhood prevalence among children in sub-Saharan Africa across 40 countries early in the HIV pandemic^38^. Five states with the highest rates of orphanhood prevalence—West Virginia, New Mexico, Mississippi, Louisiana and Kentucky (approximately 1 of every 25 children)—also had the highest poverty ranking^34^, indicating the wider implications of poverty to the premature death of parents spawning a hidden generation of orphanhood among their bereaved children^39,40^.

Over the past two decades, the prevalence of all-cause orphanhood and caregiver death decreased slightly until 2012 and then increased from 2013 to 2021 to historic levels, with intersecting crises of the overdose epidemic and COVID-19 pandemics^41,42^. Our data showed that orphanhood incidence rates due to drug overdose escalated during the pandemic, surpassing COVID-19 as the leading parental cause of death. These findings coupled with modeling and evidence suggest that both crises amplified each other syndemically. Modeling data show that the pandemic was associated with an increased drug overdose risk^43,44^, and epidemiologic data demonstrate that substance misuse appeared to increase nonadherence to CDC COVID-19 mitigation guidelines^45^.

We quantified the full scale of orphanhood and grandparent-caregiver-loss burden among children in the United States to inform the scope of action needed for an adequate public health response. Our findings show that all-cause orphanhood is over ten times greater than cause-specific orphanhood due to HIV and AIDS^35^, maternal cancers^14^, COVID-19 (ref. 13), and overdose and firearms^15,16^. A policy framework that sustains short- and long-term responses to orphanhood-linked threats for nearly three million children is necessary. This would require a comprehensive approach addressing needs shared across orphanhood causes, and the disparities affecting population subgroups, geographies and orphanhood causes. Our data suggest specific priorities for evidence-based action, from tackling drug overdose as a leading parental cause of death, addressing disparities by race and ethnicity, and prioritizing support in states with the greatest endemic poverty as they also have the highest orphanhood burden.

Effective services at the federal, state, municipal and community levels are key to supporting population-based approaches that address both all-cause and cause-specific orphanhood, and its consequences that linger over time and vary in impact. Several robust strategies can help guide policy responses: the WHO INSPIRE package for ending violence against children^46,47^ proposes life-course approaches to guide individual, familial, community and societal interventions, including addressing legal strategies, norm changes, safe environments, parenting support, income strengthening, improving response services, and education and life skills^19,20,48,49^. The ‘prevent–prepare–protect’ strategy^49^ seeks to prioritize preventing the death of parents and caregivers by accelerating equitable access to health and social services, preparing families and caregivers to provide safe and nurturing family-based support, and protecting children using evidence-based strategies that address their poverty, childhood adversity and violence risks, and strengthen their recovery. The ‘prevent–prepare–protect’ strategy can strengthen health equity shared across parental death causes by addressing shared disparities such as poverty and racism. It may also address priorities across causes, including access to health and social services, supporting kinship and family-based care, and ensuring that each affected child is protected and has access to safety, nutrition, school and nurturing family care^50^. To guide such priorities, promptly identifying, assessing and referring children experiencing orphanhood to services is paramount. For example, the state of Utah is piloting a program to support children who have lost caregivers, which engages schools to identify bereaved children^6^. Others have called for policies that include a checkbox on the death certificate to identify children living in the home of the deceased, so that bereaved children can be systematically linked in near real time to mutually reinforcing services that provide parenting, economic and education support^51^. Such interventions could also ensure that standards of care are age appropriate, inclusive and nurturing; acknowledge individual, familial, community and structural inequalities; and include children bereaved by the death of their undocumented parents or grandparents.

Disaggregated data on leading causes of orphanhood by age, sex, and racial and ethnic groups, and across geographies, may help tailor policies for affected families and communities^2,20,49^. The evolving nature of drug supply, including the proliferation of illegally made fentanyl and the resurgence of stimulants such as methamphetamine^16^, has affected younger adults still caring for children in almost every state^52,53^. We found that in 2021, fatal injuries—drug overdose, suicide, homicide and unintentional injuries—were among the top two causes of orphanhood incidence and prevalence in 48 states. In the United States, severe injuries cluster in structurally marginalized neighborhoods with higher unemployment, poverty, racial and ethnic minority residents, and lower education and income levels^54^. For communities with high prevalences of non-Hispanic Black fathers for whom homicide was a leading orphanhood cause, contextualized policies to ‘prevent’ homicide-linked deaths might include violence prevention programs, whereas ‘preventing death’ among minoritized populations for whom chronic diseases were a leading cause might involve lifestyle interventions and smoking cessation programs. The need to ‘prepare’ guardianship options for children whose parents may have an elevated risk of premature death, as recognized for parents living with HIV, may be important for other subgroups, such as those who had survived opioid overdose. Finally, addressing child bereavement to ‘protect’ mental health may require more intensive services for losses associated with greater stigma among surviving children such as those whose parents died of homicide, suicide or overdose^55^.

By addressing all-cause orphanhood and grandparent caregiver loss, our findings extend previous reports^15,16^ of the scale of overdose-linked parental loss in the United States. In particular, while overdose remains a leading cause of premature parental death for every race and ethnicity group, the leading cause of both maternal and paternal orphanhood in 2021 for every minority subgroup was not overdose, but included COVID-19, liver diseases and cirrhosis, heart disease and cancers. Our findings also highlight the importance of targeted policies, so that they are responsive to differences between structurally marginalized groups in leading causes of parental death^15^.

**Extended Data Fig. 10 |:**
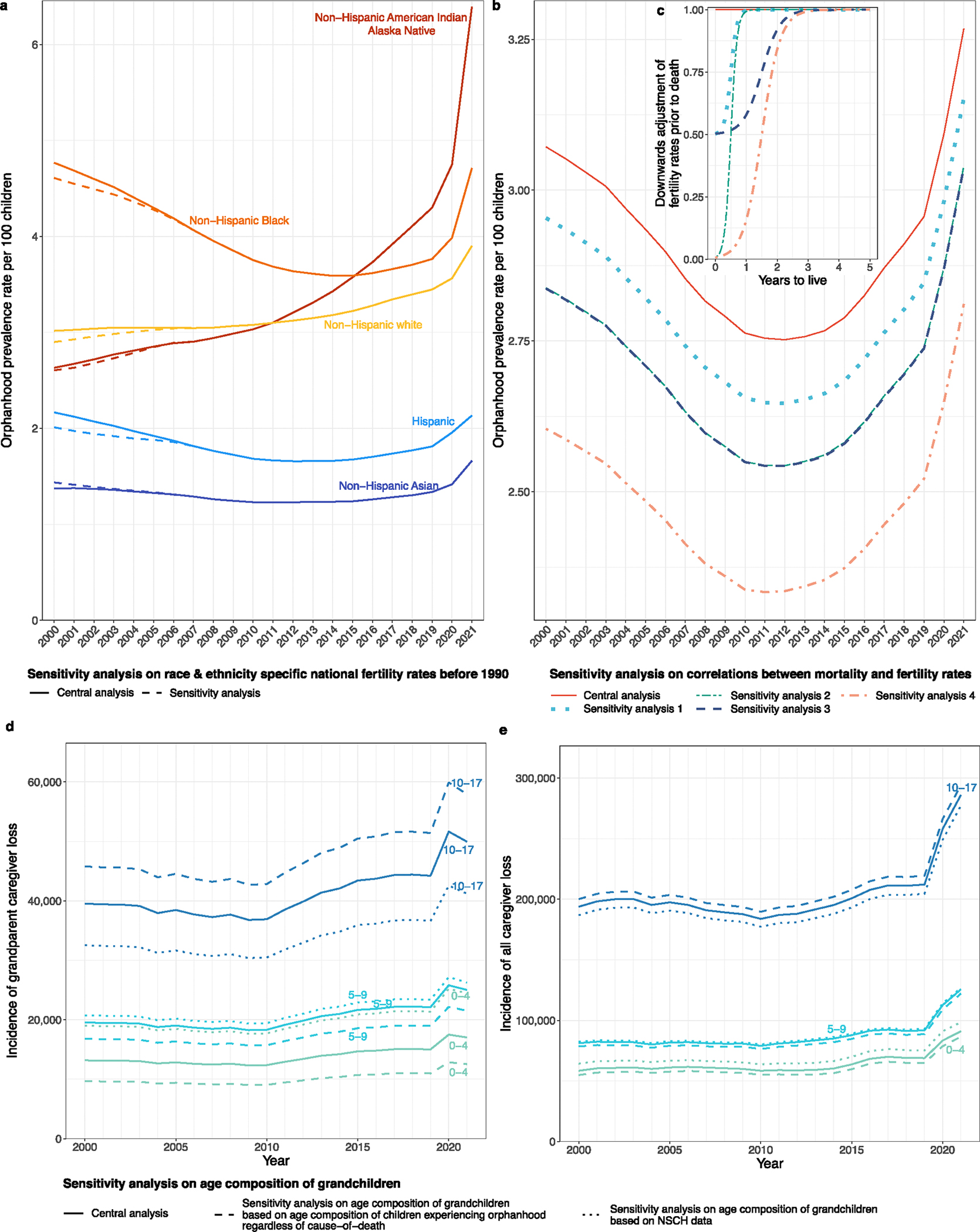
Sensitivity in national-level orphanhood and grandparent caregiver loss estimates. (**a**) Estimated national-level orphanhood prevalence rates by standardized race and ethnicity (colours) in the central analysis (solid line) and the sensitivity analysis (dashed) in which we relaxed our assumption on constant fertility rates before 1990 (Methods). We identified minor sensitivities to national orphanhood prevalence estimates up to 2007, with no impact on incidence and prevalence estimates for recent years. (**b**) Estimated national-level orphanhood prevalence rates in the central analysis (solid line in red) and in sensitivity analyses during which the impact of lower fertility rates preceding a death event were explored (dashed, dotted, dotdash and twodash lines in colour, corresponding to different multiplication factors used to model lower fertility rates prior to death events as shown in the inset (**c**). Throughout, only median estimates are shown. We found negative correlations in fertility and mortality rates could result in notably lower orphanhood prevalence estimates, whereas positive correlations could result in notably higher orphanhood prevalence estimates (not shown); note the y-axis range is between 2.3 and 3.5. (**d**) Estimated national-level loss of grandparent caregivers by age of child (colors) in the central analysis (solid line) and the two sensitivity analyses (dashed, dotted line) in which first the age composition of children experiencing grandparent caregiver loss is the same regardless of cause-of-death, and second in which the age composition was based on NCHS data (see Methods). (**e**) Estimated nationallevel loss of caregivers by age of child (colours) in the central analysis (solid line) and the same two sensitivity analyses (dashed, dotted line) as in (**d**). In (**d**) and (**e**), labels in colour represent the age of child. We identified minor sensitivities to overall incidence of caregiver loss.

Our study has several limitations. First, cause-specific estimates of children experiencing orphanhood and grandparent caregiver deaths are derived from cause-specific mortality statistics and may be underestimates^56^ for causes associated with erroneous or incomplete reporting, uncertainty in the chain of events preceding death or coding limitations—such as for COVID-19, drug overdose or suicide^57^. Furthermore, we attributed only one child per primary or secondary grandparent caregiver loss. The total numbers of children affected by caregiver loss are underestimated, particularly given our approach to de-duplicating numbers of children affected by both parent and grandparent caregiver loss. We also assumed that current mortality is unrelated to historic fertility for years before 1990, and sensitivity analyses suggest this may^15^ lead to inaccuracies in orphanhood prevalence estimates until 2007 (Extended Data Fig. 10). Similarly, we assumed no correlations between reported sex and race and ethnicity grandparent caregiver characteristics, and this may have added to inaccuracies. Publicly available state-specific data were also partly suppressed owing to small counts; therefore, we cannot exclude bias in state-specific estimates. We did not account for any of those 4.4 million US citizen–children living with an undocumented parent who died, leading to further underestimation of caregiver loss^58^. Finally, to characterize caregiver-loss prevalence in 2000–2021, we had to consider vital statistics since 1983 and consolidate changes in cause of death and race and ethnicity coding. Our sensitivity analyses (Methods and Extended Data Fig. 10), including detailed comparisons of NCHS with CDC WONDER vital statistics for each of the 21 years’ calculations (Supplementary Table 16), suggest that our incidence and prevalence estimates are robust minimum estimates of orphanhood and primary or secondary grandparent caregiver loss.

In conclusion, we estimate that at least 2.91 million children and adolescents in the United States have experienced orphanhood or the loss of a primary or secondary grandparent caregiver. These children require evidence-based responses that ensure housing stability and provide healing and support. Given unprecedented rates of drug overdose and the lasting mental health and economic impacts of the COVID-19 pandemic, it is essential to prepare for the possibility that children living with a parent negatively affected by substance misuse may experience the loss of that parent^47,59^. It is also essential to recognize that causes other than overdose resulted in the highest rates of orphanhood among all minoritized subgroups, highlighting the need for prevention strategies addressing disparities. The burden and policy relevance of orphanhood and caregiver loss has global ramifications, such as in Africa, where approximately 10% of all children had been orphaned by all-cause orphanhood in 2021, or in Latin America, where orphanhood linked to COVID-19 has been disproportionately high^2,60^. The ‘prevent–prepare–protect’ policy framework is relevant for any setting in which structural inequalities modulate health outcomes of specific subgroups. Given the scales of US national and global burden of orphanhood and grandparent caregiver loss among children, implementing policies to build their recovery and resilience is a public health and moral imperative.

## Methods

To estimate the magnitude, time trends and inequities in all-cause orphanhood and co-residing grandparent-caregiver-loss incidence and prevalence among US children, we extended a modeling methodology of COVID-19-associated orphanhood and caregiver death^20,48^ according to the Guidelines for Accurate and Transparent Health Estimates Reporting. The following sections summarize our methods.

### Study populations

The United Nations Children’s Fund defines orphanhood as children experiencing the death of one or both parents^1,2^. As previously^20^ we considered mothers of ages 15–66 years and fathers of ages 15–94 years, so the maximum ages of parents at the birth of a child were, respectively, 49 and 77years. Mortality data were recorded among US residents, and for this reason, orphanhood estimates are restricted to children of US residents. Grandparents play indispensable roles as caregivers for children^31,37,61–64^; therefore, we include as previously^20^ minimum estimates of children who lost a primary grandparent caregiver, defined as a co-residing, custodial grandparent aged 30 years or older and providing care in the absence of a parent, or providing for most of their basic needs in the presence of a parent, and children who lost a secondary grandparent caregiver defined as a co-residing grandparent aged 30 years or older serving as head of household who owns or rents the family’s housing and provides for some but not most of the basic needs of their grandchildren^32,64^. Mortality data were recorded among US residents, and so grandparent caregiver death estimates are also restricted to children of US-resident grandparent caregivers.

### National-level NCHS mortality data by rankable causes of death, 1983–2021

We obtained line-list mortality data on US residents from the NCHS Vital Statistics portal for each year from 1983 to 2021 (https://www.cdc.gov/nchs/data_access/vitalstatsonline.htm). Data were collected from 1983 onward because the corresponding children who lost a caregiver in 1983 at age 0 were of age 17 in 2000, and so entered our estimation of orphanhood prevalence in 2000. For each mortality record, we retained year of death, the corresponding codes of the underlying cause of death (ICD-9 code and 282 cause recode before 1999; ICD-10 code and 113 cause recode after 1999) and demographic data of the decedent including sex, age at death and information on race and Hispanic origin (https://www.cdc.gov/nchs/nvss/mortality_public_use_data.htm). Data on Hispanic origin were not available for 1983. Individuals of other races in 1984–1991 and individuals of more than one race in 2021 were not coded consistently and not included in this study (less than 0.019% (17,454) of line-list records were removed).

Information about the race and Hispanic origin of decedents in death certificates is typically self-reported by the surviving next of kin, or on the basis of observation in the absence of an informant^65^. Race and Hispanic origin were reported in different formats across the study period^66^, which we mapped to standardized race and Hispanic origin categories as described in Supplementary Table 6. Specifically, we grouped individuals of Hispanic origin and all individuals of non-Hispanic origin by their race, that is, ‘Hispanic’, ‘non-Hispanic American Indian or Alaska Native’, ‘non-Hispanic Asian or Pacific Islander’, ‘non-Hispanic Black’ and ‘non-Hispanic white’, and we refer to the resulting categories as ‘standardized race and ethnicity’ for simplicity. This approach to harmonizing race reporting over the study period did not necessarily make the primary data fully comparable. Earlier research shows inaccuracies are limited^67^, which indicates that the incremental implementation of multiple race reporting in the United States is unlikely to have introduced notable bias in orphanhood estimates.

The underlying cause of death is defined by the WHO as “the disease or injury which initiated the train of events leading directly to death, or the circumstances of the accident or violence which produced the fatal injury”^68^. For 1983–1998, underlying causes of death were in the data classified with the ICD-9 (ref. 69) and grouped further into 282 selected causes of death, termed ‘282 cause recode’^70^. For 1999–2021, underlying causes of death were in the data classified with the ICD-10 and grouped further into ‘113 Selected Causes of Death’^71^. We defined 53 non-overlapping rankable underlying causes of death that we termed ‘caregiver-loss causes of death’ and that apart from the following small modifications are identical to the 52 rankable underlying causes of death from the NCHS 113 Selected Causes of Death list^71,72^ (Supplementary Table 1). Specifically, we re-categorized drug-induced causes of death into ‘drug overdose’, joining the ICD-9 and ICD-10 causes of death ‘intentional self-poisoning by and exposure to nonopioid analgesics, antipyretics and antirheumatics’, ‘intentional self-poisoning by and exposure to antiepileptic, sedative–hypnotic, antiparkinsonism and psychotropic drugs, not elsewhere classified’, ‘intentional self-poisoning by and exposure to narcotics and psychodysleptics (hallucinogens), not elsewhere classified’, ‘intentional self-poisoning by and exposure to other drugs acting on the autonomic nervous system’ and ‘intentional self-poisoning by and exposure to other and unspecified drugs, medicaments and biological substances’ (E950.0–E950.5; X60–X64); ‘assault by drugs, medicaments and biological substances’ (E962.0; X85); ‘accidental poisoning by and exposure to nonopioid analgesics, antipyretics and antirheumatics’, ‘accidental poisoning by and exposure to antiepileptic, sedative–hypnotic, antiparkinsonism and psychotropic drugs, not elsewhere classified’, ‘accidental poisoning by and exposure to narcotics and psychodysleptics (hallucinogens), not elsewhere classified’, ‘accidental poisoning by and exposure to other drugs acting on the autonomic nervous system’ and ‘accidental poisoning by and exposure to other and unspecified drugs, medicaments and biological substances’ (E850–E858; X40–X44); and ‘poisoning by and exposure to nonopioid analgesics, antipyretics and antirheumatics, undetermined intent’, ‘poisoning by and exposure to antiepileptic, sedative–hypnotic, antiparkinsonism and psychotropic drugs, not elsewhere classified, undetermined intent’, ‘poisoning by and exposure to narcotics and psychodysleptics (hallucinogens), not elsewhere classified, undetermined intent’, ‘poisoning by and exposure to other drugs acting on the autonomic nervous system, undetermined intent’ and ‘poisoning by and exposure to other and unspecified drugs, medicaments and biological substances, undetermined intent’ (E980.0–E980.5; Y10–Y14). We retained these four drug overdose causes of death sub-categories as separate drug overdose subgroups for the purpose of data harmonization (see below). Correspondingly, we removed the related three drug overdose causes of death sub-categories, respectively, from ‘intentional self-harm’, ‘assault’ and ‘accidents’. We renamed these resulting three categories as ‘suicide excluding drug overdose’, ‘homicide excluding drug overdose’ and ‘unintentional injuries excluding drug overdose’. Supplementary Table 2 summarizes our aggregation of the 53 rankable causes of death into the leading parental cause-of-death groups and ‘other’ parental causes of death that we refer to in the main text and are shown in Fig. 1. The ‘other’ parental causes of death comprise the remaining 46 rankable caregiver-loss causes of death and any other causes of death that are not included in the NCHS 52 rankable causes of death.

We then mapped and aggregated line-list mortality records to the 53 rankable caregiver-loss causes of death. This was done by mapping the 1983–1998 line-list data using the 282 recodes to the 52 NCHS rankable causes of death as described in ref. 73. For drug-induced causes, we used Table 2 in ref. 74. For 1999–2021, we mapped the ICD-10 113 cause recodes to the NCHS 52 rankable causes of death based on Table A in ref. 75, and subsequently mapped the NCHS 52 rankable causes of death to the 53 rankable caregiver-loss causes of death based on the descriptions in Supplementary Table 2.

Next, we aggregated line-list death records to annualized death counts by the 53 rankable caregiver-loss causes of death for each year in 1983–2021, as well as by sex, age band (15–19, 20–24, 25–29, 30–34, 35–39, 40–44, 45–49, 50–54, 55–59, 60–64, 65–69, 70–74, 75–79, 80–84 and 85+ years) and standardized race and ethnicity of the decedent. Data on race and ethnicity were not available for 1983, and for this year, we attributed mortality counts to standardized race categories according to the age-, sex- and cause-of-death-specific standardized race compositions in 1984. To harmonize cause-of-death data from 1983 to 1999 to the ICD-10 cause-of-death classifications and avoid discontinuities in our orphanhood estimates from 1998 to 1999, we used where available the comparability ratios in Table 1 of ref. 72. Thirteen comparability ratios of rankable causes of death were not provided when underlying estimations were considered imprecise^72^, and in these cases, we set the comparability ratios to 1.

Extended Data Fig. 2 illustrates the aggregated annual mortality data among US residents by standardized race categories and Extended Data Fig. 3 by leading parental causes of death as relevant for caregiver loss and orphanhood.

### National-level live birth data, 1969–2021

We required live birth data before 1983 to calculate fertility rates and attribute children experiencing orphanhood to descendants between 1983 and 2021. This is because the children who lost a caregiver in 1983 at age 1–17 years were born between 1966 and 1982. However, due to limitations in publicly available population size data, we considered only live birth data from 1990 in the central analysis and assumed constant fertility rates before 1990. We investigated the sensitivity of our orphanhood estimates to this assumption using live birth records since 1980 together with corresponding population size estimates, and found that orphanhood prevalence estimates since 2008 were not affected by our assumptions on historic fertility, while in the sensitivity analysis, orphanhood prevalence estimates for 2000–2007 were slightly lower due to overall lower fertility rates in the 1980s (Extended Data Fig. 10a). Line-list live births with demographic data on both mothers and fathers were available and downloaded from the NCHS Vital Statistics portal (https://www.cdc.gov/nchs/data_access/vitalstatsonline.htm) for each year between 1969 and 2021. We considered only live birth records to US-resident mothers for consistency with the mortality data available. Information on residency status of fathers was not available, and we assumed fathers were also US residents in the retained birth records associated with US-resident mothers. For each live birth, we retained the year of birth, the age of mothers and fathers, and information on race and Hispanic ethnicity (https://www.cdc.gov/nchs/data_access/vitalstatsonline.htm). The age of mothers and fathers was reported by single year of age. Following ref. 20, we considered live births to women aged 15–49 years and men aged 15–77 years, to match the mortality data of women aged 15–66 years and men aged 15–94 years. In total, less than 0.012% (19,367) of line-list records were removed from further analysis because parents were outside of these age ranges or demographic information was unreported or not stated. Age information was mapped to 5 year age bands 15–19 years, …, 45–49 years for mothers and age bands 15–19 years, …, 50–54 years and 55–77 years for fathers. Information about the race and Hispanic origin of mothers and fathers was between 1969 and 2021 self-reported in different standards due to the revision of the US certificates of live births on race in 1989 and 2003^76^. Information on the race of mothers and fathers has been publicly available since 1969, and information on their ethnicity since 1978. For 1978–2021, we mapped available information on race and ethnicity to the same standardized race and ethnicity categories used to stratify the mortality data, according to Supplementary Table 7. Individuals of multiple races were excluded (less than 0.23% (398,424) of line-list natality records were removed).

Next, we aggregated line-list live birth records to annualized live birth counts to mothers (in age bands 15–19, …, 45–49 years) and fathers (in age bands 15–19, …, 55–77 years) for each year in 1969–1977, and stratified further by standardized race and ethnicity categories for each year in 1978–2021. Before 1985, data reporting was incomplete for some US states and we used NCHS sampling weights reported in each year (see Appendix A of ref. 77 to extrapolate reported live birth counts to state populations.

### National-level population size data, 1990–2021

We further required population size data of US residents in the age, sex and standardized race and ethnicity categories of the live birth data to calculate fertility rates. We obtained CDC WONDER Vintage bridged-race postcensal and ethnicity population size estimates for each year in 1990–1999 and 2000–2020 (https://wonder.cdc.gov/wonder/help/bridged-race.html#About%201990-2020) and single race population size estimates for 2021 (https://wonder.cdc.gov/wonder/help/single-race.html#About%202020-2021). Data were extracted by 5 year age bands (15–19 years, …, 85+ years) and single years of age from 75 to 77 years, and data for individuals aged 55–77 years were summed into a single age band as required for the purposes of our analyses. Information about race and Hispanic origin was self-reported during the US Census. Race-specific population size estimates were aggregated to the standardized race and ethnicity categories described in Supplementary Table 8.

For sensitivity analyses (see below), we also obtained national-level population size estimates by 5 year age bands, sex and US states without race and ethnicity stratification for each year in 1969–1989 from the US National Cancer Institute Surveillance, Epidemiology, and End Results Program (https://population.un.org/wpp/Download/Standard/Mortality/).

### Statistical analysis

#### Estimating national-level fertility rates, 1990–2021.

We calculated age-, sex- and standardized race and ethnicity-specific fertility rates in each year in 1990–2021 for women in one of the age bands a∈{15–19,…,45–49}, years and men in one of the age bands a∈{15-19,…,55-77} years according to

(1) $${FR}_{y,a,s,r}=\frac{B_{y,a,s,r}}{P_{y,a,s,r}},$$

where the number of live births and population sizes in each strata are denoted by $B_{y,a,s,r}$ and $P_{y,a,s,r}$, respectively. Calculations were done for each of the five standardized race categories ‘Hispanic’, ‘non-Hispanic American Indian or Alaska Native’, ‘non-Hispanic Asian or Pacific Islander’, ‘non-Hispanic Black’ and ‘non-Hispanic white’. In the central analysis, we assumed the same fertility rates in each year in 1966–1989 as in 1990 as shown in Extended Data Fig. 4, and considered alternative assumptions in several sensitivity analyses (see below). We calculated mortality rates in analogy to equation (1) and found correlations by standardized race and ethnicity between fertility and mortality rates that changed primarily by age of mothers and fathers, and less so over calendar years. These correlations prompted us to estimate national-level incidence and prevalence of orphanhood by standardized race and ethnicity and then sum the standardized race and ethnicity-specific estimates to obtain national-level estimates.

#### Estimating national-level orphanhood, 1983–2021.

We estimated the number of children who newly experienced orphanhood in year y,y=1983,…,2021, from the population-level mortality records of US residents in year y and the number of children each decedent was expected to leave behind. We obtained the expected number of children per US resident of age a years, sex s and standardized race and ethnicity r in year y who are of age b=0,1,…,17 years (denoted by $C_{y,a,s,r,b}$) by multiplying the corresponding fertility rates of equation (1) with pediatric survival probabilities of children born in year y-b and surviving until age b+1 (denoted with $p_{y-b,b+1}^{\text{survive}}$). Specifically

(2) $$C_{y,a,s,r,b}={FR}_{y-b,a-b,s,r}\times p_{y-b,b+1}^{\text{survive}},$$

where y ranges from 1983 to 2021, the single year of age of mothers ranges from a∈{15,…,66} years, the single year of age of fathers ranges from a∈{15,…,94} years, the standardized race and ethnicity categories are as described before, and b=0,1,…,17 years. We obtained the pediatric survival probabilities from child mortality data (https://population.un.org/wpp/Download/Standard/Mortality/) and use in equation (2) the fertility rates of the age band that includes age a-b. We then estimated the number of children aged b who newly experienced in year y the death of a parent s of age specified in 5 year age bands $a^{\prime }\in 𝒜=\{15-19,\ldots ,80-84,85+years\}$, and standardized race and ethnicity r who died of caregiver-loss cause of death c by

(3) $$O_{y,a^{\prime },s,r,b,c}^{\text{death}\;\text{of}\;\text{parent}}=C_{y,a^{\prime },s,r,b}\times D_{y,a^{\prime },s,r,c},$$

assuming that parents and their children have the same standardized race and ethnicity and where the expected number of children of parents in age bracket $a^{\prime }$, $C_{y,a^{\prime },s,r,b}$, is calculated as the mean over the expected number of children of parents aged $a\in a^{\prime }$ in equation (2). Due to the correlations between standardized race and ethnicity-specific fertility and mortality rates, we calculated equation (3) for each standardized race and ethnicity. In equation (3), we assumed that population-level fertility rates are not correlated with population-level mortality rates, which may lead to upward or downward bias in orphanhood estimates that we explored in several sensitivity analyses (see below and Extended Data Fig. 10a–c).

As orphanhood considers children who experienced the death of their mother, father or both, we are interested in the sum of equation (3) for both mothers and fathers, but need to subtract children who lost their other parent in the previous b-1 years, or who lost their other parent in the same year y. We assumed that the other parent 1-s is in the same age band a′ and of the same standardized race and ethnicity as parent s. The probability that the other biological parent of the children in equation (3) died in the same year y is based on standard life table calculations^78^, specifically the probability that an individual of sex 1-s and standardized race and ethnicity r died in year y between (continuous) age a and a+n conditional on survival up to age a, where n=5 corresponds to the width of the 5 year age bands considered. This mortality hazard is approximated using midpoints x=(a+a+5)/2 in each age interval, through

(4a) hnx=fnxS(x)=1nqnaS(a)12(S(a)+S(a+n)),

(4b) =1nqnaS(a)12S(a)+1-nqaS(a)=1n2nqa2-nqa=1nDnaPna,

where for ease of readability we have suppressed y, s and r, and Pna and Dna are respectively the estimated population sizes and observed death counts by the end of the corresponding calendar year in each 5 year age band $a^{\prime }=[a,a+5)$. The intermediate quantities in equation (4) are the approximated (unknown) mortality probability density function fnx at age midpoint x and (unknown) survival function S(a) up to age a, which can be expressed in terms of the age-specific mortality rate qna defined as the proportion of individuals alive at age a and who die before reaching age a+n in the corresponding calendar year. It is standard to estimate qna with (nDa)/(nPa+12Dna), from which equation (4) follows^78^. Using equation (4), we estimated the number of children aged b who newly experienced in year y the death of one parent due to cause-of-death c and the death of the other parent due to any cause with

(5) $$O_{y,a^{\prime },s,r,b,c}^{\text{new}\;\text{double}}{=}_{5}{\;h}_{y,x,1-s,r}\times C_{y,a^{\prime },s,r,b}\times D_{y,a^{\prime },s,r,c},$$

where x is the midpoint age in age band a′. In equation (5), we assumed that deaths among parents occurred independently of each other and ignored correlations of deaths among parents by the same cause of death such as COVID-19 (ref. 20), as well as correlations of deaths among parents who died of different causes of death. Following the same rationale, we estimated the number of children aged b who newly experienced in year y the death of one parent s due to cause-of-death c and the death of their other parent 1-s due to any cause in any of the previous i=1,…,b-1 years with

(6) Oy,a′,s,r,b,cprevious=15∑x-i∈a′ ∑x=1b-1 h5y−i,x−i,1−s,r×Cy,a′,s,r,b×Dy,a′,s,r,c.

With these considerations, we estimated the number of children aged b who newly experienced orphanhood in year y=1983,…,2021, by the death of one or both parents of age a′ and standardized race and ethnicity r who died of cause-of-death c with

(7a) $$O_{y,a^{\prime },r,b,c}^{\text{new}}=O_{y,a^{\prime },s,r,b,c}^{\text{death}\;\text{of}\;\text{parent}}+O_{y,a^{\prime },1-s,r,b,c}^{\text{death}\;\text{of}\;\text{parent}}$$

(7b) $$-\left(O_{y,a^{\prime },s,r,b,c}^{\text{new}\;\text{double}}+O_{y,a^{\prime },1-s,r,b,c}^{\text{new}\;\text{double}}\right)/2$$

(7c) $$-O_{y,a^{\prime },s,r,b,c}^{\text{previous}}-O_{y,a^{\prime },1-s,r,b,c}^{\text{previous}}.$$

Equation (7b) subtracts the children who lost in year y a parent owing to cause c and the other parent owing to any cause, which are counted twice in equation (7a). Without line-list family data and working from individual-level live birth and death statistics, the two terms $O_{y,a^{\prime },s,r,b,c}^{\text{new}\;double}$ and $O_{y,a^{\prime },1-s,r,b,c}^{\text{new}\;\text{double}}$ are not identical, and for this reason, we subtracted the average of both. Equation (7c) subtracts the children who already lost the other parent in previous years. In previous studies on COVID-19-associated orphanhood^20^, we did not consider the possibility of reinfection with COVID-19 and for this reason did not subtract children who already lost the other parent owing to COVID-19 in previous years in these studies. Further arguments show that across ages, standardized race and ethnic groups, and caregiver-loss causes of death, the values in equations (7b) and (7c) are approximately equal to orphanhood prevalence divided by four, and in the US context remain below 1% of the average values in equation (7a).

To estimate the prevalence of orphanhood in year y=2000,…,2021, we accrued the number of children who newly experienced orphanhood in the previous 17 years and current year y, while accounting for aging. Specifically, for each calendar year since 2000, we estimated the total number of children aged b=0,…,17 years in calendar year y and of race and ethnicity r who experienced orphanhood and survived in their lifetime by cause-of-death c in one or both parents with

(8) $$O_{y,r,b,c}^{\text{lifetime}}=\sum_{i=0}^{b}\;\left(\sum_{a^{\prime }}\;O_{y-i,a^{\prime },r,b-i,c}^{\text{new}}\times \prod_{j=1}^{i}\;\left(1{-}_{1}h_{y-j,r,b-j}\right)\right),$$

which sums over children who newly experienced orphanhood at younger ages in previous years conditional on survival up to the time point y+1, where hy,r,b1 is as described in equation (4). In equation (8), j does not start at 0 because we already conditioned on survival up to the current year in the incidence calculations via equation (2). For 2021, the downward adjustments in equation (8) accounting for survival amounted to less than 1.5% of the prevalence count.

Following equations (7) and (8), we derived additional key quantities such as the number of children aged b in calendar year y=1983,…,2021 and standardized race and ethnicity r who newly experienced orphanhood in year y by parental sex s and cause-of-death c

(9) $$O_{y,s,r,b,c}^{new}=\sum_{a^{\prime }}\;O_{y,a^{\prime },s,r,b,c}^{\text{death}\;\text{of}\;\text{parent}}-O_{y,a^{\prime },s,r,b,c}^{\text{previous}};$$

so maternal and paternal orphanhood incidence estimates each include children who experience the death of both parents^20,79^. Furthermore, we derived the number of children aged b in calendar year y=2000,…,2021 and standardized race and ethnicity r who experienced orphanhood in their lifetime by parent sex s and cause-of-death c by

(10) $$O_{y,s,r,b,c}^{lifetime}=\sum_{i=0}^{b}\;\left(O_{y-i,s,r,b-i,c}^{new}\times \prod_{j=1}^{i}\;\;\left({1-}_{1}h_{y-j,r,b-j}\right)\right);$$

the number of children of standardized race and ethnicity r who experienced orphanhood in calendar year y=2000,…,2021 in their lifetime by cause-of-death c in one or both parents by

(11) Oy,r,clifetime=∑a′ ∑i=017 ∑b=017-i Oy-i,a′,r,b,cnew×∏j=1i  1−h1y−j,r,b−j.

All other total numbers reported in this paper are aggregations of equations (7)–(11).

#### Estimating national-level grandparent caregiver loss, 1983–2021.

We estimated the number of children who newly experienced grandparent caregiver death in year y,y=1983,…,2021, from the population-level mortality records in year y of US residents aged 30 years and above (30+). Starting from 2010, ACS^32^ collected data on the proportion $\gamma_{y}^{\text{co-reside}}$ of adults aged 30+ years living with their grandchildren of age 17 or under in the United States, and proportions of these by sex, and separately by bridged-race and Hispanic origin (https://data.census.gov/cedsci/table?tid=ACSST5Y2019.S1002). Information about the bridged-race and Hispanic origin was self-reported. In addition, the ACS derive data on the proportion $p_{y}^{\text{most}\;\text{responsible}}$ of those who provide most of the care to any of their grandchildren through the question, ‘Is this grandparent currently responsible for providing most of the basic needs of any children under the age of 18 years and living in this house or apartment?’, and further derive information on the proportion $q_{y}^{\text{skip}\;\text{gen}}$ of those who are responsible for children in the absence of parents through column ‘Householder or spouse responsible for grandchildren with no parent of grandchildren present’ in Table S1002. From these data, we estimated the sex- and race and ethnicity-specific proportions of adults aged 30+ years who respectively are most responsible for the basic needs of grandchildren in the absence of a parent, who are most responsible for the basic needs of grandchildren in the presence of a parent and, finally, who serve as head of household who own or rent the family’s housing and provide for some but not most of the basic needs of their grandchildren^31,32^ by

(12a) $$\gamma_{y,s,r}^{\text{skip}\;\text{gen}}=\gamma_{y,s}^{\text{co-reside}}\times p_{y,r}^{\text{co-reside}}\times p_{y}^{\text{most}\;\text{responsible}}\times q_{y}^{\text{skip}\;\text{gen}}$$

(12b) $$\gamma_{y,s,r}^{\text{most}\;\text{responsible}\;\text{not}\;\text{sg}}=\gamma_{y,s}^{\text{co-reside}}\times p_{y,r}^{\text{co-reside}}\times p_{y}^{\text{most}\;\text{responsible}}\times \left(1-q_{y}^{\text{skip}\;\text{gen}}\right)$$

(12c) $$\gamma_{y,s,r}^{\text{co-reside}\;\text{not}\;\text{mr}}=\gamma_{y,s}^{\text{co-reside}}\times p_{y,r}^{\text{co-reside}}\times \left(1-p_{y}^{\text{most}\;\text{responsible}}\right).$$

From 2010 to 2021, the proportions of grandparent caregivers providing for most of the basic needs of their grandchildren with or without a parent present (respectively $\gamma_{y,s,r}^{\text{skip}\;\text{gen}}$ and $\gamma_{y,s,r}^{\text{most}\;\text{responsible}\;\text{not}\;\text{sg}}$) declined over time, whereas the proportions of grandparent caregivers providing housing and for some but not most of the needs of their grandchildren ($\gamma_{y,s,r}^{\text{co}-\text{reside}\;\mathrm{mr}}$) increased, and so we expected different trends in grandparent caregiver loss across these categories. We then assumed that each grandparent caregiver leaves upon death a minimum of one child behind (corresponding to equation (2)) and estimated the minimum number of grandchildren who newly experienced grandparent caregiver death in year y=1983,…,2021, with a US-resident grandparent caregiver aged 30+ years, sex s and standardized race and ethnicity category r who died of leading cause c by

(13) $$G_{y,s,r,c}^{x}=1\times \gamma_{y,s,r}^{x}\times \sum_{a^{\prime }\geq 30}\;D_{y,a^{\prime },s,r,c},$$

where x represents the three types of grandparent caregivers in equation (12), and assuming that $\gamma_{y,s,r}^{x}$ is for y=1983,…,2009 the same as in 2010. As illustrated in Extended Data Fig. 1, we then estimated the number of grandchildren who newly experienced the death of respectively a primary or secondary grandparent caregiver of sex s and race and ethnicity r due to caregiver-loss cause-of-death c in year y=1983,…,2021 by

(14a) $$G_{y,s,r,c}^{\text{primary}}=G_{y,s,r,c}^{\text{skip}\;\text{gen}}+G_{y,s,r,c}^{\text{most}\;\text{responsible}\;\text{not}\;\text{sg}}$$

(14b) $$G_{y,s,r,c}^{\text{secondary}}=G_{y,s,r,c}^{\text{co-reside}\;\text{not}\;\text{mr}}.$$

ACS did not collect data on whether both grandparents are alive and live with their grandchildren of age 17 or under, and for this reason, we did not adjust equation (14) further for loss of other grandparents. We investigated in sensitivity analyses our assumption that $\gamma_{y,s,r}=\sum_{x}\;\gamma_{y,s,r}^{x}$ was approximately constant from 1983 to 2010 using longitudinal United Nations Population Division data on Households and Living Arrangements of Older Persons for the United States, which suggested that the proportion of older persons who live with children or who are the primary caregivers of children has in the United States remained fairly constant since 1990 (see below). To estimate the minimum number of children who experienced grandparent caregiver death in their lifetime, we additionally need disaggregations of equation (14) by single year of age b=0,…,17 years. For the central analysis, we assumed that the age composition of $G_{y,s,r,c}^{x}$ is the same as the age composition of children who lost parents older than 30 years

(15) $$G_{y,s,r,b,c}^{x}=G_{y,s,r,c}^{x}\times \frac{\sum_{a^{\prime }\geq 30}\;O_{a^{\prime },s,r,b,c}^{\text{new}}}{\sum_{b=0}^{17}\;\sum_{a^{\prime }\geq 30}\;O_{a^{\prime },s,r,b,c}^{\text{new}}},$$

where x represents primary or secondary grandparent caregiver loss and $O_{a^{\prime },s,r,b,c}^{\text{new}}$ are obtained from equation (7) by summing over the years y=2000,…,2021, and c is one of the leading parental causes of death; these age compositions differ across causes of death while they are relatively more stable across standardized race and ethnicity. It is plausible that the age composition of $G_{y,s,r,c}^{x}$ may differ from the age composition of children who lost parents older than 30 years, and we explored alternative approaches to equation (15) in sensitivity analyses; none of these had a considerable impact on our overall estimates.

#### Estimating national-level caregiver loss, 1983–2021.

To estimate the total number of children experiencing caregiver loss defined as either orphanhood or grandparent caregiver loss, we finally sought to subtract from equation (14) those grandchildren who previously experienced orphanhood or who experienced the death of their mother or father in the same year. For the proportion $p^{\text{both}\;\text{parents}\;\text{present}}$ of grandchildren who co-resided with their grandparent and both parents at the start of year y, we assumed that either or both of the parents may have died in the remainder of the year after the ACS survey. For the latter, we considered age-, sex- and race and ethnicity-specific mortality rates and aggregated these using parent age compositions as weights to obtain the mortality rate $h_{y,s,r}^{\text{parent}}$ of a parent of sex s and race and ethnicity r in year y. Then, we subtracted from grandchildren who co-resided with their grandparent and both parents at the start of year y the proportion $(h_{y,M,r}^{\text{parent}}+h_{y,F,r}^{\text{parent}}-h_{y,M,r}^{\text{parent}}h_{y,F,r}^{\text{parent}})\times 6/12$ that we expected to additionally experience the loss of one or both of their parents in the 6 months on average after the ACS survey. For the proportion $1-p^{\text{both}\;\text{parents}\;\text{present}}$ of grandchildren who co-resided with their grandparent and one parent at the start of year y, we assumed that in a proportion $p^{\text{other}\;\text{parent}\;\text{died}}$ the other parent had died previously and removed these children experiencing grandparent caregiver loss from the caregiver loss count. In the remaining proportion, we assumed again that either or both of the parents may have died in the remainder of the year after the ACS survey. Finally, for grandchildren who lived in skip generation households at the start of year y, we assumed a proportion $p^{\text{skip}\;\text{gen}\;\text{parent}\;\text{died}}$ previously lost either their mother, their father or both, and removed these children experiencing grandparent caregiver loss from the caregiver loss count. In the remaining proportion, we assumed again that either or both of the parents may have died in the remainder of the year after the ACS survey. We thus obtained for each year y=1983,…,2021 the estimated number of US children aged 0–17 years newly experiencing the loss of a caregiver of sex s and race and ethnicity r due to caregiver-loss cause-of-death c through

(16a) $$L_{y,s,r,c}^{\text{new}}=O_{y,s,r,c}^{\text{new}}+G_{y,s,r,c}^{\text{de-dup}}$$

(16b) $$G_{y,s,r,c}^{\text{de-dup}}=G_{y,s,r,c}^{\text{skip}\;\text{gen}}\times (1-p^{\text{skip}\;\text{gen}\;\text{parent}\;\text{died}})\times$$

(16c) $$\left(1-\left(h_{y,M,r}^{\text{parent}}+h_{y,F,r}^{\text{parent}}-h_{y,M,r}^{\text{parent}}h_{y,F,r}^{\text{parent}}\right)\frac{6}{12}\right)$$

(16d) $$+\left[G_{y,s,r,c}^{\text{most}\;\text{responsible}\;\text{not}\;\text{sg}}+G_{y,s,r,c}^{\text{co-reside}\;\text{not}\;\text{mr}}\right]\times$$

(16e) $$\left(p^{\text{both}\;\text{parents}\;\text{present}}+\left(1-p^{\text{both}\;\text{parents}\;\text{present}}\right)\left(1-p^{\text{other}\;\text{parent}\;\text{died}}\right)\right)\times$$

(16f) $$\left(1-\left(h_{y,M,r}^{\text{parent}}+h_{y,F,r}^{\text{parent}}-h_{y,M,r}^{\text{parent}}h_{y,F,r}^{\text{parent}}\right)\frac{6}{12}\right).$$

In equation (16), we specified $p^{\text{skip}\;\text{gen}\;\text{parent}\;\text{died}}=11\%$ and $p^{\text{other}\;\text{parent}\;\text{died}}=11\%$ based on US data indicating that the large majority of grandparent caregivers provide care owing to child maltreatment, and/or parents experiencing substance misuse or incarceration^31,37^. We set $p^{\text{both}\;\text{parents}\;\text{present}}=70\%$ based on UN data on US household composition and living arrangements of persons aged 60 or over (https://www.un.org/development/desa/pd/data/living-arrangements-older-persons), and further supported by ref. 31.

To estimate caregiver-loss prevalence, we noted that the grandparent caregiver-loss estimates are derived from cross-sectional data, and we therefore summed the orphanhood prevalence estimates in equation (8) and the de-duplicated annual grandparent-caregiver-loss contributions in equation (16d)–(16f) conditional of survival of the children

(17) $$L_{y,s,r,b,c}^{\text{lifetime}}=O_{y,s,r,b,c}^{\text{lifetime}}+\sum_{i=0}^{b}\;\left(G_{y-i,s,r,b-i,c}^{\text{de-dup}}\times \prod_{j=0}^{i}\;\;\left({1-}_{1}h_{y-j,r,b-j}\right)\right),$$

where the age-specific, de-duplicated annual grandparent-caregiver-loss contributions are calculated analogously to equation (15).

#### Uncertainty quantification in national estimates.

To capture uncertainty, we followed a similar phenomenological approach as in ref. 20 and added Poisson noise around mortality, natality and population size data for all year, sex, age, standardized race and ethnicity, and cause-of-death strata that was co-monotonized across years to maximize uncertainty due to temporal autocorrelations^80^. We generated 1,000 Poisson noise random variables around each live birth, death and population size count by year, sex, age, race and ethnicity and then ranked the Poisson counts by size. For uncertainty in mortality data, we additionally considered uncertainty related to the harmonization of cause-of-death counts from 1983 to 1999 to the ICD-10 classification to avoid discontinuities in orphanhood estimates between 1998 and 1999. Specifically, we resampled the comparability ratios 1,000 times, assuming these were normally distributed with the standard deviations reported in Table 1 of ref. 72 where available, and multiplied these ratios with the Poisson noise mortality data. Then, we repeated orphanhood incidence and prevalence estimates using the randomized live birth, deaths and population size data, and calculated 2.5% and 97.5% quantiles to generate 95% UIs around median estimates.

For grandparent caregiver loss, we accounted for uncertainty in the estimated number of adults aged 30+ years living with grandchildren and in the attribution to sex, and standardized race and ethnicity groups using uncertainty ranges published by ACS. ACS provide for each of their published estimates margins of error that correspond to 90% confidence intervals. We converted the 90% margins of error into standard deviations and bootstrap re-sampled the available totals and separate proportions 1,000 times assuming normal distributions around their median estimates and the corresponding standard deviations, multiplied the bootstrap resampled totals and proportions, and then divided by the corresponding population sizes. Across years, we found that these sources of uncertainty amounted on average to 95% bootstrap intervals on the order of ±0.81% of the median estimate of the number of Hispanic women aged 30+ years who live with grandchildren, ±8.23% among non-Hispanic American Indian or Alaska Native women, ±1.92% among non-Hispanic Asian women, ±0.95% among non-Hispanic Black women and ±0.68% among non-Hispanic white women, and similarly for men. We similarly generated 1,000 resampled data sets, repeated grandparent-caregiver-loss incidence and prevalence estimates on each data set, and then calculated 2.5% and 97.5% quantiles to generate 95% uncertainty intervals around median estimates.

#### Estimating state-level orphanhood, 2021.

To guide policy at the state level, we further estimated orphanhood and grandparent caregiver loss in each of the 50 US states and District of Columbia. We focused on estimating orphanhood incidence and prevalence in each state in 2021, by the leading parental causes of death only (COVID-19, heart disease, drug overdose, homicide excluding drug overdose, malignant neoplasms, suicide excluding drug overdose, and unintentional injuries excluding drug overdose (Supplementary Table 2) and one leading cause of orphanhood in South Dakota: chronic liver disease and cirrhosis). To obtain state-level prevalence estimates for 2021, we had as before to estimate state-level incidence from 2004, for which state-level fertility rates were required from 1987. Overall, we proceeded analogously as for the national estimations, but obtained data from CDC WONDER (https://wonder.cdc.gov/Deaths-by-Underlying-Cause.html) as state-level mortality data were not publicly available from NCHS from 2005 onward (https://www.cdc.gov/nchs/data_access/vitalstatsonline.htm). Counts below 10 were suppressed in CDC WONDER, and for this reason, we performed all estimations without stratification by standardized race and ethnicity. We adjusted for data suppression and known biases in this approach that are due to correlations in mortality and fertility rates across standardized race and ethnicity. We note these are modeled adjustments and we cannot exclude that this resulted in bias in state-specific orphanhood estimates.

We extracted annual death counts by state, sex, age bands (15–19 years, …, 95–99 years, ≥100 years) and cause of death (ICD-10 113 Selected Causes of Death) from the CDC WONDER mortality portal (https://wonder.cdc.gov/Deaths-by-Underlying-Cause.html) from 2005 to 2021, and combined these data with the previously described, cause-specific NCHS mortality data sets from 2004 for which information on the state of residence of the decedent was publicly available. For the leading parental causes of death, the average discrepancy in the state-, sex-, age- and cause-of-death-specific data from CDC WONDER was, when aggregated to the national level and compared with the corresponding NCHS mortality data, 22.9% among women and 13.5% among men. As a first step, we imputed suppressed entries with a value of 2, which reduced average discrepancies to 6.80% among women and 2.53% among men. To account for these discrepancies, as a second step, we adjusted the state-level mortality counts according to

(18a) $$D_{y,a^{\prime },s,l,c}^{*}=\eta_{y,a^{\prime },s,c}\times D_{y,a^{\prime },s,l,c}$$

(18b) $$\eta_{y,a^{\prime },s,c}=D_{y,a^{\prime },s,c}/\sum_{l}\;D_{y,a^{\prime },s,l,c},$$

where y is the period 2005 to 2021 and a′ denotes the age band of the parent, s the sex of the parent, c the leading parental cause-of-death of the parent, $D_{y,a^{\prime },s,c}$ the national-level deaths derived from the line-list NCHS data in equation (3), and $D_{y,a^{\prime },s,l,c}$ the state-specific deaths derived from CDC WONDER for each of the 50 US states and District of Columbia, indexed by l. The suppression-adjusted, cause-specific, state-level mortality counts matched the cause-specific, national-level mortality counts well, except when all deaths $D_{y,a^{\prime },s,c,l}$ were entirely suppressed across all states, which was the case for deaths due to homicide in women aged 55 years or older (Extended Data Fig. 7). Overall, these suppression adjustments did not noticeably change the contribution of causes of death to mortality, because the majority of death counts were not suppressed when the data were aggregated to leading parental causes of death.

We next extracted live birth counts for mothers by state and age bands (15–19, …, 44–49 years) from the CDC WONDER natality portal (https://wonder.cdc.gov/natality.html) from 2005 to 2021, and combined these data with the previously described NCHS live birth data sets from 1987 to 2004 for which information on the state of residence of mothers was available. We proceeded analogously for fathers using the age bands 15–19, …, 44–49, 50–54, 55+ years from 2016 to 2021, as natality records were not available by demographic characteristics of fathers from 2005 to 2015. Data suppression was not an issue for the stratifications required to estimate orphanhood: for 2005–2021, the average discrepancy in the CDC WONDER and NCHS natality data sets was 0.026% for mothers and 0.032% for fathers, compared with male NCHS live birth data with ages up to 77 years. To compute fertility rates at the state level, we further extracted age- and sex-stratified annual population size estimates for each state from 1987 to 1989 from the National Cancer Institute Surveillance, Epidemiology, and End Results database (https://population.un.org/wpp/Download/Standard/Mortality/), and CDC WONDER from 1990 to 2021 (https://wonder.cdc.gov/wonder/help/bridged-race.html#About%201990-2020, https://wonder.cdc.gov/single-race-population.html). State-level fertility rates were calculated as in equation (1) when live birth counts were available, but without stratification by race and ethnicity. For mothers, live birth counts were not publicly available for women aged 44–49 years in several years and 8 states (Alaska, Delaware, Montana, North Dakota, South Dakota, Vermont, West Virginia and Wyoming), and in these cases, we interpolated state-specific fertility rates with locally estimated scatterplot smoothing as implemented in the R **stat** package version 4.2.3 with span argument 0.85. For Wyoming, no data were publicly available after 2019, and so interpolation was not possible and we used 2019 values. For fathers, live birth counts were not publicly available from 2005 to 2015, and for these years, we estimated state-specific fertility rates with locally estimated scatterplot smoothing with span argument 0.85, and using NCHS data from 2000 to 2004 and CDC WONDER data from 2016 to 2020.

We then estimated state-specific incidence and prevalence of orphanhood from the suppression-adjusted state-level, cause-specific mortality counts and partially imputed fertility rates as outlined in equations (7)–(11), with one modification. As described above, the state-level mortality and fertility data were not disaggregated by race and ethnicity. To account for potential bias arising from correlations in mortality and fertility rates across race and ethnicity, we compared the national-level estimates of the number of children who newly experienced orphanhood in equation (9) to the state-specific estimates with the correction factors

(19) $$v_{y,s,c}=\sum_{r,a^{\prime },b}\;O_{y,a^{\prime },s,r,b,c}^{\text{new}}/\sum_{l,a^{\prime },b}\;O_{y,a^{\prime },s,l,b,c}^{\text{new}}.$$

Overall, the correction factors $v_{y,s,c}$ tended to be close to one, except for ‘homicide excluding drug overdose’ in women and ‘other’ caregiver-loss causes of death (Extended Data Fig. 7). We then adjusted the state-specific estimates as follows:

(20) $$O_{y,a^{\prime },s,l,b,c}^{\mathrm{new*}}=v_{y,s,c}\times O_{y,a^{\prime },s,l,b,c}^{\mathrm{new}},$$

and used equation (20) to calculate the state-specific analogs to equations (8)–(11). The resulting state-level orphanhood incidence estimates matched the national-level estimates with discrepancies of up to 0.5%. Exactly matching estimates could have been obtained with age-specific correction factors, but we felt this would result in a false sense of accuracy in the state-level orphanhood incidence estimates. Our correction factors ensure only that our state-level estimates sum to a total close to our national-level estimates, and it is possible that the true state-level orphanhood counts could differ from our estimates.

#### Estimating state-level grandparent caregiver loss, 2021.

We estimated the number of children who newly experienced grandparent caregiver death in year y,y=2004,…,2021 from the state-level mortality records in year y of US residents aged 30 years and above (30+), and assuming that each decedent who is estimated to have been a grandparent caregiver in each state leaves a minimum of one grandchild behind (corresponding to equation (2)). Overall, we proceeded as for the national-level estimation of grandparent caregiver loss, as ACS also published state-specific data on adults aged 30+ years who live with grandchildren of age 17 or under (https://data.census.gov/cedsci/table?tid=ACSST5Y2019.S1002). For each year y=2010,…,2021, we calculated the expected number of adults aged 30+ years who live with their grandchildren of age 17 or under by multiplying the corresponding total co-resident numbers with the sex-specific proportions for each state, and then divided these with corresponding population sizes to obtain the proportions $\gamma_{y,s,l}^{x}$ corresponding to equation (12). Overall, these proportions were considerably more uncertain than in the national-level analysis owing to smaller sample sizes. For y=2004,…,2009, we assumed that $\gamma_{y,s,l}^{x}$ is the same as in 2010. We estimated the minimum number of grandchildren who newly experienced primary or secondary grandparent caregiver death in year y=2004,…,2021, with a US-resident grandparent caregiver aged 30+ years, sex s and state category l who died of leading parental cause-of-death c by

(21a) $$G_{y,s,l,c}^{\text{primary}}=G_{y,s,l,c}^{\text{skip}\;\text{gen}}+G_{y,s,l,c}^{\text{most}\;\text{responsible}\;\text{not}\;\text{sg}}$$

(21b) $$G_{y,s,l,c}^{\text{secondary}}=G_{y,s,l,c}^{\text{co-reside}\;\text{not}\;\text{mr}}.$$

To estimate the minimum number of children who experienced grandparent caregiver death in their lifetime in each state, we additionally need disaggregations of equation (21) by single year of age b=0,…,17 years. For the central analysis, we assumed that the age composition of $G_{y,s,l,c}^{x}$ is the same as the age composition of children who lost parents older than 30 years in the same state

(22) $$G_{y,s,l,b,c}^{x}=G_{y,s,l,c}^{x}\times \frac{\sum_{a^{\prime }\geq 30}\;\;O_{a^{\prime },s,l,b,c}^{\mathrm{new*}}}{\sum_{b=0}^{17}\;\;\sum_{a^{\prime }\geq 30}\;\;O_{a^{\prime },s,l,b,c}^{\mathrm{new*}}},$$

where $O_{a^{\prime },s,l,b,c}^{\mathrm{new*}}$ are obtained from equation (20) by summing over the years y=2004,…,2021, and c is one of the leading parental causes of death.

#### Uncertainty quantification in state-level estimates.

As for uncertainty quantification at the national level, we added co-monotonized Poisson noise around state-specific mortality, natality and population size data for all year, sex, age and cause-of-death strata. We generated 1,000 Poisson noise random variables around each live birth, death and population size count by year, state, sex, age, race and ethnicity and then ranked the Poisson counts by size. We then repeated orphanhood incidence and prevalence estimates using the randomized data, and then calculated 2.5% and 97.5% quantiles to generate 95% uncertainty intervals around median estimates.

To estimate grandparent caregiver loss, we accounted for uncertainty in the estimated number of adults aged 30+ years living with grandchildren by US state using uncertainty ranges published by ACS and as described above for national-level uncertainty quantification. As expected from smaller sample sizes at the state level across years, we found that uncertainty in the proportions $\gamma_{y,s,l}=\sum_{x}\;\gamma_{y,s,l}^{x}$ was in some states considerably larger than for the national analysis, with 95% bootstrap intervals of up to ±13.6% of the median estimates. We similarly generated 1,000 resampled data sets, repeated grandparent-caregiver-loss incidence and prevalence estimates on each data set, and then calculated 2.5% and 97.5% quantiles to generate 95% uncertainty intervals around median estimates.

#### Estimating racial and ethnic groups impacted by leading causes of caregiver loss in US states, 2021.

Characterizing the racial and ethnic groups of children that are impacted by orphanhood and grandparent caregiver loss at the state level is challenging owing to limits in publicly available data. We focused on estimating and comparing 2021 orphanhood and grandparent-caregiver -loss prevalence rates by standardized race and ethnicity in the 10 US states with the highest orphanhood prevalence rates and for the primary (first-ranked) parental cause of death only: West Virginia (‘drug overdose’), New Mexico (‘drug overdose’), Mississippi (‘unintentional injuries excluding drug overdose’), Louisiana (‘drug overdose’), Kentucky (‘drug overdose’), Tennessee (‘drug overdose’), Alabama (‘heart disease’), Oklahoma (‘unintentional injuries excluding drug overdose’), Ohio (‘drug overdose’) and Florida (‘drug overdose’). Throughout, we obtained publicly available data from CDC WONDER (https://wonder.cdc.gov/Deaths-by-Underlying-Cause.html) from 2005 onward (https://www.cdc.gov/nchs/data_access/vitalstatsonline.htm). We adjusted for data suppression, but note that these are modeled adjustments and we cannot exclude that this resulted in bias in state-, race- and ethnicity-specific orphanhood estimates.

Annualized live birth counts were extracted as described for the state-level analysis for 2005 to 2019, but now also stratified by bridged-race and Hispanic origins, and then combined with NCHS live birth data from 1987 to 2004. For 2020–2021, live birth counts to women were not available by bridged-race at the state level. Analogously, annual death counts for the leading parental causes of death were compiled as described above for 2005 to 2021, stratified into the standardized race and ethnicity categories in Supplementary Table 6, and then combined with the NCHS mortality data sets from 2004. Suppressed values were imputed by 1 and adjusted further for suppression as in equation (18). We followed previous criteria on data reliability and excluded from consideration for each state those race and ethnicity groups that had more than two age bands with fewer than 20 live birth counts^81^. Supplementary Table 15 lists the corresponding strata as ‘small populations’ and also describes the average completeness of live birth records for the years 1995–2004 when directly comparable line-list live birth records were also available from NCHS. For the year, state, sex, race and ethnicity strata that were not excluded, we considered the corresponding mortality data for the leading caregiver-loss cause of death in each state. Again, we excluded from consideration those standardized race and ethnicity groups that had more than 2 age bands with fewer than 20 death counts^81^. Supplementary Table 15 lists the corresponding strata as ‘small death counts’ and also describes the average completeness of death records for 1999–2004 when directly comparable line-list death records were also available from NCHS. Extended Data Fig. 9a,b illustrates these data completeness evaluations on data from New Mexico for women. Population size estimates for each state were obtained from the CDC WONDER population size portal for 1990 to 2021 (https://wonder.cdc.gov/Bridged-Race-v2020.HTML, https://wonder.cdc.gov/single-race-population.html) by bridged race and converted to the standardized race and ethnicity categories described in Supplementary Table 8. We then estimated state-, race- and ethnicity-specific incidence and prevalence of orphanhood as outlined in equations (7)–(11), except that female state-, race- and ethnicity-specific fertility rates in 2020–2021 were assumed to be as in 2019 owing to limitations in publicly available data. To investigate estimation accuracy, we compared the resulting sum of the state-, race- and ethnicity-specific orphanhood incidence estimates to the previous state-specific orphanhood incidence estimates (Extended Data Fig. 9c). For Alaska and Oklahoma, the sum of the race and ethnicity- and state-specific orphanhood incidence estimates was more than 20% below the state-specific orphanhood incidence estimates attributable to the leading parental cause of death; Supplementary Table 15 lists these states as ‘large discrepancy in estimates’. The table then reports, for all remaining states, 2021 state-, race- and ethnicity-specific orphanhood prevalence rate estimates that are attributable to the leading parental cause of death.

### Sensitivity analyses

#### Sensitivity in mortality counts to mortality data processing.

In the central analysis, we derived mortality counts by year, sex, age band, standardized race and ethnicity, and caregiver-loss causes of death from NCHS line-list mortality records from 1983 to 2021. Aggregate mortality counts are also available from CDC WONDER (https://wonder.cdc.gov/Deaths-by-Underlying-Cause.html) from 1999 to 2021. We extracted data from the NCHS Vital Statistics portal because this allowed us to use the same data source across all years, bypass data suppression and incorporate uncertainties in standardized race and ethnicity reporting. The CDC WONDER mortality counts allowed us to check our in-house data aggregations, if we obtain data from CDC WONDER at coarser population strata. For this purpose, we extracted annual death counts by sex and age bands (15–19 years, …, 95–99 years, ≥100 years) from the CDC WONDER mortality portal (https://wonder.cdc.gov/Deaths-by-Underlying-Cause.html) from 2000 to 2021. The overall mortality counts that we aggregated from the NCHS line-list data were identical to the CDC WONDER mortality data without cause-of-death stratification. Secondly, we extracted annual death counts by sex, age bands (15–19 years, …, 95–99 years, ≥100 years) and cause of death (ICD-10 113 Selected Causes of Death) from the CDC WONDER mortality portal (https://wonder.cdc.gov/Deaths-by-Underlying-Cause.html) from 2000 to 2021, without stratification by race and ethnicity. We then compared the mortality counts from the two data sources for each year and the leading parental causes of death. We found that across years, the maximum discrepancy in the CDC WONDER counts relative to the NCHS mortality counts was 0.0068% in women and 0.0057% in men, which reflected data suppression especially due to homicide excluding drug overdose in women, and overall indicated consistency of our data with that from CDC WONDER (Supplementary Table 16).

#### Sensitivity in live birth counts to live birth data processing.

In the central analysis, we derived live birth counts by year, sex, age band, and standardized race and ethnicity from NCHS line-list natality records from 1969 to 2021. Aggregate live birth counts are also available from CDC WONDER (https://wonder.cdc.gov/natality.html) from 1995 to 2021. We chose to extract data from the NCHS Vital Statistics portal because this allowed us to use the same data source across all years. The CDC WONDER live birth counts allowed us to check our in-house data aggregations. For this purpose, we extracted annual live birth counts from the CDC WONDER natality portal for women by age bands 15–19 years, …, 45–49 years from 2000 to 2021, and separately for men by age bands 15–19 years, …, 55+ years from 2016 to 2021 without stratification by race and ethnicity, which avoids data suppression. We then compared the live birth counts from the two data sources for each year and found that both data sets matched across all years.

#### Sensitivity in national-level orphanhood estimates to assumptions on historic fertility rates.

In the central analysis, we assumed that male and female fertility rates by age and standardized race and ethnicity were constant in 1966–1989 and in 1990 (Extended Data Fig. 4). This assumption was made because population size data stratified by more than three race categories were not publicly available before 1990. Considering that age- and sex-specific live birth data were available by race and ethnicity since 1978, we estimated in this sensitivity analysis the historic composition of population sizes by race and ethnicity in 1980–1989, and then updated the corresponding fertility rates and national-level orphanhood incidence and prevalence estimates. More specifically, we considered time trends in the proportion of each race and ethnicity in each 5 year age band and both sexes in the US intercensal population size estimates in 1990 to 1995, and extrapolated these with linear models backward in time to 1980–1989. We do not think that this estimation approach resulted in accurate estimates of population sizes, but rather that this approach conveys possible sensitivities in our orphanhood estimates. We identified minor sensitivities to national orphanhood prevalence estimates up to 2007, with no impact on incidence and prevalence estimates for recent years (Extended Data Fig. 10a).

#### Sensitivity in national-level orphanhood estimates to potentially correlated fertility rates.

In the central analysis, we assumed that population-level fertility rates were not correlated with population-level mortality rates. We considered in sensitivity analyses possible deviations from this assumption that might lead to upward bias in our central estimates. For example, a person who died in year y may have experienced poor health in the preceding years y-1,y-2, … and in this case may also have been less likely to mother or father a child in years y,y-1,y-2, …. We modeled this possibility through four scenarios of dampened fertility rates close to individual death events. Specifically, we considered cumulative logistic adjustment factors that dampened fertility rates to either zero or half of the corresponding population-level-, year-, age-, sex- and standardized race and ethnicity-specific fertility rates in the year of death y. We also considered sensitivity analyses so that the onset of lower fertility rates preceded the year of death y by 1 or 3 years (Extended Data Fig. 10a–c). We found that orphanhood incidence estimates were up to 8.4% lower than in the central analysis, and orphanhood prevalence estimates were up to 15% lower than in the central analysis (Extended Data Fig. 10a–c). It is also possible that our central orphanhood estimates might be biased downward. For example, women experiencing premature death are more likely economically disadvantaged and in turn are more likely to have had reduced access to contraceptives and corresponding higher fertility rates^82^, or earlier sexual debut resulting in higher cumulative fertility until death. It is also possible that overall, at the population level, there is no strong correlation between fertility and mortality rates especially as the lag between birth and death events is typically relatively large, and in the absence of data, we have opted for this middle approach in the central analysis.

#### Sensitivity in national-level grandparent-caregiver-loss estimates to assumptions on the age of children experiencing loss of a grandparent caregiver.

In the central analysis, we considered the age composition of children experiencing orphanhood in equation (15), as a proxy to the age composition of children experiencing grandparent caregiver loss. Calculations were done independently for each leading parental cause of death. We performed two analyses to characterize the sensitivity of this approach to national-level grandparent-caregiver-loss estimates. First, we repeated calculations using as proxy the age composition of children experiencing orphanhood across all causes of caregiver loss, that is

(23) $$G_{y,s,r,b,c}^{\mathrm{new}}=G_{y,s,r,c}^{\mathrm{new}}\times \frac{\sum_{c,a^{\prime }\geq 30}\;\;O_{a^{\prime },s,r,b,c}^{\mathrm{new*}}}{\sum_{c,b=0}^{17}\;\;\sum_{a^{\prime }\geq 30}\;\;O_{a^{\prime },s,r,b,c}^{\mathrm{new*}}}.$$

Second, we used data from the ‘Topical survey’ of the National Survey of Children’s Health (https://www.census.gov/data/tables/time-series/demo/families/children.html), filtered to respondents reporting to be grandparents and living with at least one child in the household. Respondents were asked to report on demographic characteristics including the age and race and ethnicity of one randomly selected child. We pooled these data across 6 years, from 2016 to 2021, to characterize the age composition of children who live with grandparents due to small sample sizes in each survey after filtering (approximately 2,500 grandparents per survey round). In the sensitivity analyses, we found that age-specific grandparent caregiver incidence estimates deviated by up to ±26.8% and ±44.4% of the central estimate across years; however, as parental death contributes more to caregiver loss, age-specific caregiver-loss incidence estimates deviated by up to ±6.3% and ±10.0% of the central estimate across years (Extended Data Fig. 10d–e).

#### Sensitivity in national-level caregiver-loss estimates to assumptions on historic numbers of grandparent caregivers.

In the central analysis, we assumed that the proportion of adults aged 30+ years who live with their grandchildren of age 17 or under (denoted $\gamma_{y,s,r}$) was the same in the years y=1983,…,2009 as in 2010. This assumption was made because data were not available before 2010. To investigate the implications of this assumption, we considered longitudinal United Nations Population Division data on Households and Living Arrangements of Older Persons for the United States since 1960 (https://www.un.org/development/desa/pd/data/living-arrangements-older-persons). These data indicate that the proportion of older persons who live with children or who are the primary caregivers of children has in the United States remained fairly constant since 1980 and, for this reason, suggests that our assumptions on the historic number of grandparent caregivers are unlikely to have a substantive impact on caregiver-loss estimates.

## Extended Data

## Supplementary Material

Supplementary material to main manuscript

Published research brief

## Figures and Tables

**Table 1 |:** Policy summary

**Background**	Deaths of parents and grandparent caregivers threaten child well-being owing to losses of financial support, housing, safety, family stability and care globally, but are rarely recognized as a public health crisis. For some important causes, orphanhood is preventable—for example, prescription drug overdose due to depression, as recent reports highlight. For all causes, evidence shows that long-term negative impacts of orphanhood and co-residing caregiver loss are preventable: with strong positive parenting, and economic and educational support for surviving caregivers, children's outcomes can be equal to those of non-orphaned children. Little is known about the full burden, trends and disparities in all-cause and cause-specific orphanhood and caregiver loss, beyond estimates from select causes.

**Main findings and limitations**	In the United States, incidence and prevalence trends in orphanhood and co-residing primary and secondary grandparent caregiver loss increased in total by 49.5% and 7.9%, respectively, from 2000 to 2021. In 2021, 2.38 million children (3.4% of children) had in their lifetime experienced prevalent orphanhood, 217,000 (0.3%) primary grandparent caregiver loss (providing most basic needs) and 342,000 (0.5%) secondary grandparent caregiver loss (providing housing but not most other basic needs). Evidence shows that co-residing grandparents also provide salient nurturing and practical care. We found that 66.8% of orphaned children lost their father in their lifetime, and 33.2% lost their mother. Populations disproportionately affected by orphanhood included 5.2% of all adolescents and 6.4% and 4.7%, respectively, of non-Hispanic American Indian or Alaska Native and non-Hispanic Black children. In 2021, prevalent orphanhood of 3% or greater was pervasive across states and highest (approximately 5%) in West Virginia, New Mexico, Mississippi, Louisiana and Kentucky. Since 2021, parental death due to drug overdose increased to historic levels, surpassing COVID-19, as the leading cause of incident and prevalent orphanhood nationally during the COVID-19 pandemic. However, the highest cause of orphanhood in 2021 for every minoritized subgroup was not drug overdose but varied from heart disease to COVID-19 for paternally orphaned non-Hispanic American Indian or Alaska Native, non-Hispanic Black, Hispanic and non-Hispanic Asian children. Variations in leading causes of maternal orphanhood included cirrhosis, COVID-19 and cancer in these minoritized subgroups. Our state-specific analyses showed that orphanhood due to fatal injuries—drug overdose, suicide, homicide and unintentional injuries—exceeded those linked to leading chronic diseases, with fatal injuries and chronic diseases among the top two causes of orphanhood in 48 states. A key limitation is our possible underestimation of the number of children affected by orphanhood and caregiver death associated with erroneous or incomplete reporting; underreporting of caregiver loss is also likely owing to the unavailability of data on the numbers of children in the care of co-resident grandparents.

**Policy implications**	Policies and programs that provide healing and support for three million children in the United States who have experienced orphanhood and caregiver loss may contribute to reducing acute and long-term negative effects of this adverse childhood experience. Globally, caregiver-loss prevalences are probably above 4% among children in countries where fertility rates and parental death rates are higher than in the United States. Evidence highlights three essential components of orphanhood prevention and response that effectively promote their recovery and resilience and can guide policy investments for both all-cause and cause-specific orphanhood and caregiver loss: (1) prevent death of parents and caregivers through timely prevention and treatment of leading causes of death and ensure access to health and mental health care for all, (2) prepare families to provide safe and nurturing alternative care and (3) protect children affected by orphanhood and vulnerabilities, through grief and mental health counseling, and parenting, economic and educational support. Given the scope of orphanhood and caregiver loss and associated threats to mental and physical health, and lifelong well-being of children, these strategies can be contextualized and prioritized.

**Table 2 |:** Trends in all-cause orphanhood and grandparent caregiver (primary and secondary) loss from 2000 to 2021, before and during the COVID-19 pandemic

	Before the COVID-19 pandemic			Since the COVID-19 pandemic			
	2000(number of children)	2019(number of children)	2000–2019 changes	2020(number of children)	2021(number of children)	2019–2021 changes	2000–2021 changes
**Incidence (*n* (95% UI))**							
Total	330,413	368,892	+11.7%	448,894	494,036	+33.9%	+49.5%
	(302,093, 362,310)	(338,918, 402,024)	(+11.0%, +12.3%)	(414,154, 487,052)	(457,957, 533,274)	(+32.6%, +35.0%)	(+47.2%, +51.5%)
Orphanhood	262,036	292,118	+11.5%	360,192	407,377	+39.4%	+55.5%
	(237,122, 290,407)	(265,553, 321,908)	(+10.9%, +12.0%)	(329,378, 394,370)	(374,905, 442,874)	(+37.6%, +41.2%)	(+52.5%, +58.1%)
Primary grandparent caregiver loss	31,574	29,976	−5.1%	34,014	32,738	+9.2%	+3.7%
	(30,112, 33,202)	(28,741, 31,292)	(−6.3%, −3.8%)	(32,652, 35,388)	(31,419, 34,111)	(+7.9%, +10.7%)	(+2.3%, +5.2%)
Secondary grandparent caregiver loss	40,106	50,404	+25.6%	58,938	57,914	+14.9%	+44.4%
	(38,123, 42,349)	(48,142, 52,813)	(+24.1%, +27.1%)	(56,441, 61,500)	(55,404, 60,587)	(+14.1%, +15.9%)	(+42.5%, +46.1%)
**Incidence rate per 100 children (rate (95% UI))**							
Total	0.46	0.50	+10.6%	0.62	0.71	+41.0%	+56.0%
	(0.42, 0.50)	(0.46, 0.55)	(+9.9%, +11.2%)	(0.57, 0.67)	(0.66, 0.77)	(+39.6%, +42.2%)	(+53.5%, +58.0%)
Orphanhood	0.36	0.40	+10.4%	0.49	0.59	+46.9%	+62.1%
	(0.33, 0.40)	(0.36, 0.44)	(+9.8%, +10.9%)	(0.45, 0.54)	(0.54, 0.64)	(+44.9%, +48.7%)	(+59.1%, +64.9%)
Primary grandparent caregiver loss	0.04	0.04	−6.0%	0.05	0.05	+15.0%	+8.2%
	(0.04, 0.05)	(0.04, 0.04)	(−7.2%, −4.7%)	(0.04, 0.05)	(0.05, 0.05)	(+13.6%, +16.6%)	(+6.7%, +9.7%)
Secondary grandparent caregiver loss	0.06	0.07	+24.3%	0.08	0.08	+21.1%	+50.6%
	(0.05, 0.06)	(0.07, 0.07)	(+22.8%, +25.9%)	(0.08, 0.08)	(0.08, 0.09)	(+20.2%, +22.0%)	(+48.7%, +52.4%)
**Prevalence (*n* (95% UI))**							
Total	2,700,285	2,663,581	−1.3%	2,772,616	2,912,817	+9.4%	+7.9%
	(2,450,266, 2,983,377)	(2,419,032, 2,940,459)	(−1.5%, −1.3%)	(2,521,580, 3,055,449)	(2,654,936, 3,202,040)	(+8.9%, +9.8%)	(+7.3%, +8.4%)
Orphanhood	2,220,606	2,159,537	−2.7%	2,251,322	2,378,250	+10.1%	+7.1%
	(2,001,053, 2,472,064)	(1,941,498, 2,408,054)	(−3.0%, −2.6%)	(2,027,683, 2,505,227)	(2,148,223, 2,638,221)	(+9.6%, +10.6%)	(+6.7%, +7.4%)
Primary grandparent caregiver loss	221,982	212,330	−4.3%	215,695	217,560	+2.4%	−2.0%
	(208,916, 235,830)	(201,542, 223,777)	(−5.2%, −3.5%)	(204,853, 227,214)	(206,708, 228,924)	(+2.2%, +2.7%)	(−3.1%, −1.0%)
Secondary grandparent caregiver loss	281,227	315,613	+12.2%	330,258	342,214	+8.4%	+21.7%
	(262,509, 300,825)	(298,427, 333,736)	(+10.8%, +13.7%)	(312,383, 348,923)	(323,917, 361,420)	(+8.2%, +8.6%)	(+19.9%, +23.4%)
**Prevalence rate per 100 children (rate (95% UI))**							
Total	3.73	3.64	−2.3%	3.81	4.20	+15.2%	+12.5%
	(3.39, 4.12)	(3.31, 4.02)	(−2.4%, −2.2%)	(3.46, 4.20)	(3.83, 4.61)	(+14.7%, +15.6%)	(+11.9%, +13.0%)
Orphanhood	3.07	2.95	−3.7%	3.09	3.43	+16.0%	+11.7%
	(2.76, 3.42)	(2.66, 3.29)	(−3.9%, −3.5%)	(2.78, 3.44)	(3.10, 3.80)	(+15.4%, +16.5%)	(+11.3%, +12.0%)
Primary grandparent caregiver loss	0.31	0.29	−5.3%	0.30	0.31	+7.9%	+2.2%
	(0.29, 0.33)	(0.28, 0.31)	(−6.2%, −4.4%)	(0.28, 0.31)	(0.30, 0.33)	(+7.6%, +8.1%)	(+1.1%, +3.2%)
Secondary grandparent caregiver loss	0.39	0.43	+11.1%	0.45	0.49	+14.2%	+26.9%
	(0.36, 0.42)	(0.41, 0.46)	(+9.7%, +12.6%)	(0.43, 0.48)	(0.47, 0.52)	(+14.0%, +14.4%)	(+25.1%, +28.7%)

## Data Availability

All data used to calculate estimates of mortality and orphanhood are publicly available via Zenodo at https://doi.org/10.5281/zenodo.11423744 (ref. 83). Mortality and natality data were sourced from the NCHS (1983–2021 and 1968–2021, respectively) (https://www.cdc.gov/nchs/data_access/vitalstatsonline.htm) with the utils (of version 4.2.3) package in R. Mortality and natality data after 2005 at the state level were sourced from CDC WONDER (mortality data 2005–2021: https://wonder.cdc.gov/Deaths-by-Underlying-Cause.html; natality data 2005–2021: https://wonder.cdc.gov/natality.html). Population data from 1969 to 1989 were sourced from https://seer.cancer.gov/popdata/singleages.html. Population data from 1990 to 2020 and 2021 were sourced from CDC WONDER (https://wonder.cdc.gov/bridged-race-population.html; https://wonder.cdc.gov/single-race-population.html, respectively). Child mortality data were sourced from United Nations (https://population.un.org/wpp/Download/Standard/Mortality/). Household data from 2010 to 2021 were sourced from American Community Survey (for example, year 2019: https://data.census.gov/table/ACSST5Y2019.S1002).
