# Supplementary material for: Orphanhood and caregiver death among children in the United States by all-cause mortality, 2000–2021
Source: Nat Med. 2025 Jan 10;31(2):672–83. doi: 10.1038/s41591-024-03343-6 (PMC11835524; doi:10.1038/s41591-024-03343-6)
Supplement: Supplementary file 1 — Supplementary Tables 1–19. [file 41591_2024_3343_MOESM1_ESM.pdf]

nature medicine

Supplementary information https://doi.org/10.1038/s41591-024-03343-6

Orphanhood and caregiver death among children in the United States by all-cause mortality, 2000–2021

In the format provided by the authors and unedited

List of Tables

1 Definitions of caregiver loss causes of death used in this analysis relative to the 52 rankable causes of death included in the NCHS 113 Selected Causes of Death. . . . S2
2 Aggregation of 53 rankable caregiver loss causes of death into leading parental causes of death groups. . . . S4
3 Children newly experiencing orphanhood in the US in 2021, by all caregiver loss causes of death. . . . S7
4 Maternal and paternal orphanood, orphanhood, grandparent caregiver loss and all caregiver loss incidence among US children in 2021 by US state. . . . S8
5 Maternal and paternal orphanood, orphanhood, grandparent caregiver loss and all caregiver loss prevalence among US children in 2021 by US state. . . . S11
6 Mapping of single-race mortality data to standardized race categories and Hispanic origin. . . . S14
7 Mapping of single-race live birth data to standardized race categories and Hispanic origin. . . . S15
8 Mapping of single-race population size data to standardized race categories and Hispanic origin. . . . S16
9 Age groups of children newly experiencing orphanhood in the US from 2000 to 2021. . . . S17
10 Sex of parent of children newly experiencing orphanhood in the US from 2000 to 2021. . . . S18
11 Racial and ethnicity of children newly experiencing orphanhood in the US from 2000 to 2021. . . . S19
12 Leading causes of orphanhood among US children in 2021 by race and ethnicity and sex of parent. . . . S20
13 Leading causes of orphanhood among US children in 2019 by race and ethnicity and sex of parent. . . . S23
14 Leading causes of orphanhood among US children in 2021 by US state. S26
15 Race and ethnicity groups most affected by the leading parental cause of death in the ten US states with highest orphanhood prevalence rates in 2021. . . . S31
16 Comparison of aggregated line-list US mortality data from NCHS to mortality counts from CDC WONDER by leading parental cause of death. . . . S33
17 Leading causes of orphanhood among US children from 2000 to 2021. S35
18 Leading causes of primary grandparent caregiver loss among US children from 2000 to 2021. . . . S36
19 Leading causes of secondary grandparent caregiver loss among US children from 2000 to 2021. . . . S37

S1 Supplementary Tables

ID	Caregiver loss causes of death	NCHS 52 rankable underlying causes of death†	Further modifications‡	
			ICD-9	ICD-10
1	Acute bronchitis and bronchiolitis	Acute bronchitis and bronchiolitis	-	-
2	Acute poliomyelitis	Acute poliomyelitis	-	-
3	Alzheimer disease	Alzheimer disease	-	-
4	Anemias	Anemias	-	-
5	Aortic aneurysm and dissection	Aortic aneurysm and dissection	-	-
6	Arthropod-borne viral encephalitis	Arthropod-borne viral encephalitis	-	-
7	Atherosclerosis	Atherosclerosis	-	-
8	Cerebrovascular diseases	Cerebrovascular diseases	-	-
9	Certain conditions originating in the perinatal period	Certain conditions originating in the perinatal period	-	-
10	Cholelithiasis and other disorders of gallbladder	Cholelithiasis and other disorders of gallbladder	-	-
11	Chronic liver disease and cirrhosis	Chronic liver disease and cirrhosis	-	-
12	Chronic lower respiratory diseases	Chronic lower respiratory diseases	-	-
13	Complications of medical and surgical care	Complications of medical and surgical care	-	-
14	Congenital malformations, deformations and chromosomal abnormalities	Congenital malformations, deformations and chromosomal abnormalities	-<br>-	-
15	COVID-19	COVID-19	-	-
16	Diabetes mellitus	Diabetes mellitus	-	-
17	Diseases of appendix	Diseases of appendix	-	-
18	Heart disease	Diseases of heart	-	-
19	Drug overdose		Adding:	Adding:
	... Assault by drugs		E962.0	X85
	... Intentional self-poisoning		E950.0-E950.5	X60-X64
	... Poisoning with undetermined intent		E980.0-E980.5	Y10-Y14
	... Unintentional self-poisoning		E850-E858	X40-X44
20	Enterocolitis due to Clostridium difficile	Enterocolitis due to Clostridium difficile	-	-
21	Essential hypertension and hypertensive renal disease	Essential hypertension and hypertensive renal disease	-	-
22	Hernia	Hernia	-	-
23	Homicide, excluding drug overdose	Assault	Removing E962.0 from (E960-E969)	Removing X85 from (*U01 - *U02,X85-Y09,Y87.1)
24	Human immunodeficiency virus	Human immunodeficiency virus	-	-
25	Hyperplasia of prostate	Hyperplasia of prostate	-	-
26	In situ neoplasms, benign neoplasms and neoplasms of uncertain or unknown behavior	In situ neoplasms, benign neoplasms and neoplasms of uncertain or unknown behavior	-<br>-	-
27	Infections of kidney	Infections of kidney	-	-

See footnotes at the end of table.

Supplementary Table 1: Definitions of caregiver loss causes of death used in this analysis relative to the 52 rankable causes of death included in the NCHS 113 Selected Causes of Death.

ID	Caregiver loss causes of death	NCHS 52 rankable underlying causes of death†	Further modifications‡ ICD-9	ICD-10
28	Inflammatory diseases of female pelvic organs	Inflammatory diseases of female pelvic organs	-	-
29	Influenza and pneumonia	Influenza and pneumonia	-	-
30	Legal intervention	Legal intervention	-	-
31	Malaria	Malaria	-	-
32	Malignant neoplasms	Malignant neoplasms	-	-
33	Measles	Measles	-	-
34	Meningitis	Meningitis	-	-
35	Meningococcal infection	Meningococcal infection	-	-
36	Nephritis, nephrotic syndrome and nephrosis	Nephritis, nephrotic syndrome and nephrosis	-	-
37	Nutritional deficiencies	Nutritional deficiencies	-	-
38	Operations of war and their sequelae	Operations of war and their sequelae	-	-
39	Parkinson disease	Parkinson disease	-	-
40	Peptic ulcer	Peptic ulcer	-	-
41	Pneumoconioses and chemical effects	Pneumoconioses and chemical effects	-	-
42	Pneumonitis due to solids and liquids	Pneumonitis due to solids and liquids	-	-
43	Pregnancy, childbirth and the puerperium	Pregnancy, childbirth and the puerperium	-	-
44	Salmonella infections	Salmonella infections	-	-
45	Scarlet fever and erysipelas	Scarlet fever and erysipelas	-	-
46	Septicemia	Septicemia	-	-
47	Shigellosis and amebiasis	Shigellosis and amebiasis	-	-
48	Suicide, excluding drug overdose	Intentional self-harm	Removing E950.0-E950.5 from E950-E959	Removing X60-X64 from (*U03, X60-X84, Y87.0)
49	Syphilis	Syphilis	-	-
50	Tuberculosis	Tuberculosis	-	-
51	Unintentional injuries, excluding drug overdose	Accidents	Removing E850-E858 from (E800-E869, E880-E929)	Removing X40-X44 from (V01-X59, Y85-Y86)
52	Viral hepatitis	Viral hepatitis	-	-
53	Whooping cough	Whooping cough	-	-
	Other	Other	-	-

†: The subset of the NCHS 113 Selected cause list with the reported cause names. Empty cells represent that the corresponding causes of death are not in the NCHS 113 Selected cause list. ‡: '-' denotes there were no modifications from the NCHS 52 rankable causes of death to caregiver loss causes of death.

Supplementary Table 1: Definitions of caregiver loss causes of death used in this analysis relative to the 52 rankable causes of death included in the NCHS 113 Selected Causes of Death (continued).

LaTeX template

ID	Caregiver loss causes of death†	ICD-9 code range	ICD-10 code range	Aggregation into leading parental causes-of-death groups
1	Acute bronchitis and bronchiolitis	(466)	(J20-J21)	Other
2	Acute poliomyelitis	(045)	(A80)	Other
3	Alzheimer disease	(331.0)	(G30)	Other
4	Anemias	(280-285)	(D50-D64)	Other
5	Aortic aneurysm and dissection	(441)	(I71)	Other
6	Arthropod-borne viral encephalitis	(062-064)	(A83-A84,A85.2)	Other
7	Atherosclerosis	(440)	(I70)	Other
8	Cerebrovascular diseases	(430-434,436-438)	(I60-I69)	Other
9	Certain conditions originating in the perinatal period	(760-771.2,771.4-779)	(P00-P96)	Other
10	Cholelithiasis and other disorders of gallbladder	(574-575)	(K80-K82)	Other
11	Chronic liver disease and cirrhosis	(571)	(K70,K73-K74)	Other
12	Chronic lower respiratory diseases	(490-494,496)	(J40-J47)	Other
13	Complications of medical and surgical care	(E870-E879,E930-E949)	(Y40-Y84,Y88)	Other
14	Congenital malformations, deformations and chromosomal abnormalities	(740-759)	(Q00-Q99)	Other
15	COVID-19	–	(U07.1)	COVID-19
16	Diabetes mellitus	(250)	(E10-E14)	Other
17	Diseases of appendix	(540-543)	(K35-K38)	Other
18	Heart disease	(390-398,402,404,410-429)	(I00-I09,I11,I13,I20-I51)	Heart disease
19	Drug overdose			Drug overdose
	... Assault by drugs	(E962.0)	(X85)	
	... Intentional self-poisoning	(E950.0-E950.5)	(X60-X64)	
	... Poisoning with undetermined intent	(E980.0-E980.5)	(Y10-Y14)	
	... Unintentional self-poisoning	(E850-E858)	(X40-X44)	
20	Enterocolitis due to Clostridium difficile	–	(A04.7)	Other
21	Essential hypertension and hypertensive renal disease	(401,403)	(I10,I12,I15)	Other
22	Hernia	(550-553)	(K40-K46)	Other

See footnotes at the end of table.

Supplementary Table 2: Aggregation of 53 rankable caregiver loss causes of death into leading parental causes of death groups.

ID	Caregiver loss causes of death†	ICD-9 code range	ICD-10 code range	Aggregation into leading parental causes-of-death groups
23	Homicide excluding drug overdose	(E960-E961.9, E962.1-E969)	(*U01 - *U02, X86-Y09, Y87.1)	Homicide excluding drug overdose
24	Human immunodeficiency virus	(042-044)	(B20-B24)	Other
25	Hyperplasia of prostate	(600)	(N40)	Other
26	In situ neoplasms, benign neoplasms and neoplasms of uncertain or unknown behavior	(210-239)	(D00-D48)	Other
27	Infections of kidney	(590)	(N10-N12,N13.6,N15.1)	Other
28	Inflammatory diseases of female pelvic organs	(614-616)	(N70-N76)	Other
29	Influenza and pneumonia	(480-487)	(J09-J18)	Other
30	Legal intervention	(E970-E978)	(Y35,Y89.0)	Other
31	Malaria	(084)	(B50-B54)	Other
32	Malignant neoplasms	(140-208)	(C00-C97)	Malignant neoplasms
33	Measles	(055)	(B05)	Other
34	Meningitis	(320-322)	(G00,G03)	Other
35	Meningococcal infection	(036)	(A39)	Other
36	Nephritis, nephrotic syndrome and nephrosis	(580-589)	(N00-N07,N17-N19,N25-N27)	Other
37	Nutritional deficiencies	(260-269)	(E40-E64)	Other
38	Operations of war and their sequelae	(E990-E999)	(Y36,Y89.1)	Other
39	Parkinson disease	(332)	(G20-G21)	Other
40	Peptic ulcer	(531-534)	(K25-K28)	Other
41	Pneumoconioses and chemical effects	(500-506)	(J60-J66,J68,U07.0)	Other
42	Pneumonitis due to solids and liquids	(507)	(J69)	Other
43	Pregnancy, childbirth and the puerperium	(630-676)	(O00-O99)	Other
44	Salmonella infections	(002-003)	(A01-A02)	Other
45	Scarlet fever and erysipelas	(034.1-035)	(A38,A46)	Other
46	Septicemia	(038)	(A40-A41)	Other

See footnotes at the end of table.

Supplementary Table 2: Aggregation of 53 rankable caregiver loss causes of death into leading parental causes-of-death groups.

LaTeX template

ID	Caregiver loss causes of death†	ICD-9 code range	ICD-10 code range	Aggregation into leading parental causes-of-death groups
47	Shigellosis and amebiasis	(004,006)	(A03,A06)	Other
48	Suicide excluding drug overdose	(E950.6-E959)	(*U03, X65-X84, Y87.0)	Suicide excluding drug overdose
49	Syphilis	(090-097)	(A50-A53)	Other
50	Tuberculosis	(010-018)	(A16-A19)	Other
51	Unintentional injuries excluding drug overdose	(E800-E849, E859-E869, E880-E929)	(V01-X39, X45-59, Y85-Y86)	Unintentional injuries excluding drug overdose
52	Viral hepatitis	(070)	(B15-B19)	Other
53	Whooping cough	(033)	(A37)	Other
	Other	*	*	Other

†: To account for all mortality data, we grouped death counts into the 'Other' category if the corresponding cause of death was not among the 53 caregiver loss causes of death. Drug overdose subcategories are listed separately for clarity on our mappings. '–' denotes there was no corresponding ICD-9 cause of death. '*' denotes any other ICD-9 codes or ICD-10 codes not listed above.

Supplementary Table 2: Aggregation of 53 rankable caregiver loss causes of death into leading parental causes-of-death groups (continued).

Caregiver loss cause of death	Children newly experiencing orphanhood (rank)	(n)	(proportion)	Adult deaths (rank)	(n)	(proportion)	Ratio
Drug overdose	#1	71,065	17.44%	#8	105,121	3.08%	0.68
COVID-19	#2	56,956	13.98%	#3	414,764	12.14%	0.14
Heart disease	#3	49,429	12.13%	#1	692,140	20.25%	0.07
Malignant neoplasms	#4	46,327	11.37%	#2	601,350	17.59%	0.08
Unintentional injuries excluding drug overdose	#5	33,709	8.27%	#6	121,587	3.56%	0.28
Suicide excluding drug overdose	#6	20,487	5.03%	#12	42,667	1.25%	0.48
Chronic liver disease and cirrhosis	#7	17,329	4.25%	#10	56,176	1.64%	0.31
Homicide excluding drug overdose	#8	14,906	3.66%	#17	24,534	0.72%	0.61
Diabetes mellitus	#9	11,223	2.75%	#9	102,626	3.00%	0.11
Cerebrovascular diseases	#10	8,255	2.03%	#4	161,918	4.74%	0.05
Chronic lower respiratory diseases	#11	4,166	1.02%	#5	141,630	4.14%	0.03
Septicemia	#12	3,668	0.90%	#15	40,915	1.20%	0.09
Nephritis, nephrotic syndrome and nephrosis	#13	3,660	0.90%	#11	54,059	1.58%	0.07
Influenza and pneumonia	#14	2,830	0.69%	#14	41,503	1.21%	0.07
Essential hypertension and hypertensive renal disease	#15	2,523	0.62%	#13	42,599	1.25%	0.06
Human immunodeficiency virus	#16	2,183	0.54%	#24	4,931	0.14%	0.44
Pregnancy, childbirth and the puerperium	#17	1,997	0.49%	#32	1,657	0.05%	1.21
Congenital malformations, deformations and chromosomal abnormalities	#18	1,577	0.39%	#25	4,899	0.14%	0.32
Aortic aneurysm and dissection	#19	1,108	0.27%	#21	9,958	0.29%	0.11
Pneumonitis due to solids and liquids	#20	942	0.23%	#18	19,870	0.58%	0.05
In situ neoplasms, benign neoplasms and neoplasms of uncertain or unknown behavior	#21	775	0.19%	#20	16,051	0.47%	0.05
Complications of medical and surgical care	#22	724	0.18%	#22	5,964	0.17%	0.12
Anemias	#23	632	0.16%	#23	5,920	0.17%	0.11
Viral hepatitis	#24	559	0.14%	#30	3,550	0.10%	0.16
Legal intervention	#25	411	0.10%	#35	650	0.02%	0.63
Peptic ulcer	#26	327	0.08%	#29	3,971	0.12%	0.08
Nutritional deficiencies	#27	318	0.08%	#19	17,422	0.51%	0.02
Alzheimer disease	#28	200	0.05%	#7	119,084	3.48%	0.00
Parkinson disease	#29	150	0.04%	#16	38,440	1.12%	0.00
Cholelithiasis and other disorders of gallbladder	#30	122	0.03%	#26	4,473	0.13%	0.03
Enterocolitis due to Clostridium difficile	#31	110	0.03%	#28	4,089	0.12%	0.03
Hernia	#32	85	0.02%	#31	2,452	0.07%	0.03
Infections of kidney	#33	84	0.02%	#33	1,230	0.04%	0.07
Meningitis	#34	43	0.01%	#38	454	0.01%	0.09
Atherosclerosis	#35	40	0.01%	#27	4,185	0.12%	0.01
Tuberculosis	#36	25	0.01%	#36	595	0.02%	0.04
Inflammatory diseases of female pelvic organs	#37	21	0.01%	#40	212	0.01%	0.10
Acute bronchitis and bronchiolitis	#38	0	0.00%	#41	84	0.00%	0.00
Arthropod-borne viral encephalitis	#39	0	0.00%	#47	6	0.00%	0.00
Certain conditions originating in the perinatal period	#40	0	0.00%	#43	40	0.00%	0.00
Diseases of appendix	#41	0	0.00%	#39	426	0.01%	0.00
Hyperplasia of prostate	#42	0	0.00%	#34	709	0.02%	0.00
Malaria	#43	0	0.00%	#48	6	0.00%	0.00
Meningococcal infection	#44	0	0.00%	#45	18	0.00%	0.00
Operations of war and their sequelae	#45	0	0.00%	#46	9	0.00%	0.00
Pneumoconioses and chemical effects	#46	0	0.00%	#37	561	0.02%	0.00
Salmonella infections	#47	0	0.00%	#42	58	0.00%	0.00
Shigellosis and amebiasis	#48	0	0.00%	#49	6	0.00%	0.00
Syphilis	#49	0	0.00%	#44	38	0.00%	0.00
Whooping cough	#50	0	0.00%	#50	2	0.00%	0.00
Acute poliomyelitis	#51	0	0.00%	#51	0	0.00%	0.00
Measles	#52	0	0.00%	#52	0	0.00%	0.00
Scarlet fever and erysipelas	#53	0	0.00%	#53	0	0.00%	0.00
Other	-	48,411	11.88%	-	502,181	14.69%	0.10

Supplementary Table 3: Children newly experiencing orphanhood in the US in 2021, by all caregiver loss causes of death.

Incidence in 2021

US state[‖]	Maternal Orphanhood[†] (n, 95% UI)	Maternal Orphanhood[†] rate per 100 children (rate, 95% UI)	Paternal Orphanhood[‡] (n, 95% UI)	Paternal Orphanhood[‡] rate per 100 children (rate, 95% UI)	Orphanhood[*] (n, 95% UI)	Orphanhood[*] rate per 100 children (rate, 95% UI)	Grandparent Caregiver Loss (n, 95% UI)	Grandparent Caregiver Loss rate per 100 children (rate, 95% UI)	All Caregiver Loss (n, 95% UI)	All Caregiver Loss rate per 100 children (rate, 95% UI)
United States	137,202	0.20	270,188	0.39	407,377	0.59	90,635	0.13	494,036	0.71
	(123,350, 152,612)	(0.18, 0.22)	(251,565, 290,278)	(0.36, 0.42)	(374,905, 442,874)	(0.54, 0.64)	(86,851, 94,735)	(0.13, 0.14)	(457,957, 533,274)	(0.66 , 0.77)
Alabama	2,936	0.26	5,070	0.45	8,006	0.71	2,036	0.18	9,928	0.88
	(2,228, 3,768)	(0.20, 0.34)	(4,084, 6,117)	(0.36, 0.55)	(6,312, 9,886)	(0.56, 0.88)	(1,873,2,226)	(0.17,0.20)	(8,091, 11,991)	(0.72, 1.07)
Alaska	403	0.22	700	0.39	1,103	0.61	175	0.10	1,274	0.71
	(210, 836)	(0.12, 0.47)	(400, 1,096)	(0.22, 0.61)	(610, 1,932)	(0.34, 1.08)	(137,244)	(0.08,0.14)	(750, 2,169)	(0.42, 1.21)
Arizona	3,741	0.23	7,237	0.45	10,978	0.68	2,327	0.14	13,213	0.82
	(2,883, 4,744)	(0.18, 0.29)	(6,065, 8,563)	(0.38, 0.53)	(8,948, 13,308)	(0.55, 0.82)	(2,138,2,530)	(0.13,0.16)	(11,001, 15,785)	(0.68, 0.98)
Arkansas	1,687	0.24	2,787	0.40	4,474	0.64	1,187	0.17	5,608	0.80
	(1,156, 2,373)	(0.16, 0.34)	(2,103, 3,585)	(0.30, 0.51)	(3,259, 5,958)	(0.46, 0.85)	(1,047,1,336)	(0.15,0.19)	(4,252, 7,239)	(0.60, 1.03)
California	13,303	0.15	32,118	0.37	45,422	0.52	11,892	0.14	56,843	0.65
	(11,700, 14,989)	(0.13, 0.17)	(29,555, 34,696)	(0.34, 0.40)	(41,255, 49,685)	(0.47, 0.57)	(11,449,12,428)	(0.13,0.14)	(52,282, 61,719)	(0.60, 0.70)
Colorado	2,207	0.18	4,394	0.35	6,600	0.53	1,032	0.08	7,596	0.61
	(1,594, 2,948)	(0.13, 0.24)	(3,519, 5,397)	(0.28, 0.43)	(5,113, 8,345)	(0.41, 0.67)	(943,1,152)	(0.08,0.09)	(6,046, 9,459)	(0.49, 0.76)
Connecticut	1,001	0.14	2,339	0.32	3,340	0.46	724	0.10	4,046	0.55
	(642, 1,417)	(0.09, 0.19)	(1,739, 3,023)	(0.24, 0.41)	(2,381, 4,440)	(0.33, 0.61)	(648,801)	(0.09,0.11)	(3,011, 5,221)	(0.41, 0.72)
Delaware	361	0.17	602	0.29	963	0.46	269	0.13	1,226	0.59
	(197, 687)	(0.09, 0.33)	(338, 886)	(0.16, 0.43)	(535, 1,573)	(0.26, 0.76)	(224,351)	(0.11,0.17)	(761, 1,918)	(0.37, 0.92)
District of Columbia	192	0.15	432	0.34	624	0.50	89	0.07	710	0.56
	(95, 316)	(0.08, 0.25)	(242, 689)	(0.19, 0.55)	(337, 1,005)	(0.27, 0.80)	(59,118)	(0.05,0.09)	(403, 1,121)	(0.32, 0.89)
Florida	9,680	0.23	19,078	0.44	28,758	0.67	6,864	0.16	35,314	0.82
	(8,316, 11,114)	(0.19, 0.26)	(17,217, 21,034)	(0.40, 0.49)	(25,532, 32,149)	(0.60, 0.75)	(6,560,7,243)	(0.15,0.17)	(31,865, 39,078)	(0.74, 0.91)
Georgia	5,794	0.23	10,014	0.40	15,808	0.63	3,437	0.14	19,094	0.76
	(4,720, 6,982)	(0.19, 0.28)	(8,605, 11,544)	(0.34, 0.46)	(13,325, 18,526)	(0.53, 0.73)	(3,195,3,683)	(0.13,0.15)	(16,376, 22,083)	(0.65, 0.87)
Hawaii	336	0.11	882	0.29	1,218	0.40	575	0.19	1,776	0.58
	(196, 651)	(0.06, 0.21)	(548, 1,287)	(0.18, 0.42)	(744, 1,938)	(0.24, 0.64)	(463,684)	(0.15,0.22)	(1,195, 2,604)	(0.39, 0.86)
Idaho	626	0.13	1,319	0.28	1,945	0.41	412	0.09	2,349	0.50
	(339, 1,057)	(0.07, 0.23)	(847, 1,906)	(0.18, 0.41)	(1,186, 2,963)	(0.25, 0.63)	(338,479)	(0.07,0.10)	(1,527, 3,450)	(0.33, 0.74)
Illinois	4,486	0.16	9,474	0.34	13,960	0.50	3,036	0.11	16,875	0.60
	(3,580, 5,465)	(0.13, 0.19)	(8,098, 10,944)	(0.29, 0.39)	(11,678, 16,409)	(0.42, 0.59)	(2,854,3,247)	(0.10,0.12)	(14,439, 19,524)	(0.52, 0.70)
Indiana	3,250	0.20	5,953	0.38	9,204	0.58	1,796	0.11	10,914	0.69
	(2,428, 4,123)	(0.15, 0.26)	(4,891, 7,120)	(0.31, 0.45)	(7,319, 11,243)	(0.46, 0.71)	(1,663,1,950)	(0.10,0.12)	(8,914, 13,097)	(0.56, 0.83)
Iowa	939	0.13	1,836	0.25	2,776	0.38	513	0.07	3,262	0.44
	(586, 1,447)	(0.08, 0.20)	(1,312, 2,471)	(0.18, 0.34)	(1,898, 3,918)	(0.26, 0.53)	(450,574)	(0.06,0.08)	(2,335, 4,471)	(0.32, 0.61)
Kansas	1,193	0.17	2,295	0.33	3,488	0.50	650	0.09	4,114	0.59
	(757, 1,771)	(0.11, 0.25)	(1,614, 3,053)	(0.23, 0.43)	(2,371, 4,824)	(0.34, 0.69)	(583,755)	(0.08,0.11)	(2,935, 5,546)	(0.42, 0.79)
Kentucky	2,628	0.26	4,610	0.45	7,238	0.71	1,731	0.17	8,878	0.87
	(1,977, 3,404)	(0.19, 0.34)	(3,737, 5,590)	(0.37, 0.55)	(5,714, 8,994)	(0.56, 0.89)	(1,591,1,893)	(0.16,0.19)	(7,236, 10,783)	(0.71, 1.06)

See footnotes at the end of table.

Supplementary Table 4: Maternal and paternal orphanood, orphanhood, grandparent caregiver loss and all caregiver loss incidence among US children in 2021 by US state.

Incidence in 2021										
**US state**[‖]	**Maternal Orphanhood**[†]		**Paternal Orphanhood**[‡]		**Orphanhood**[*]		**Grandparent Caregiver Loss**		**All Caregiver Loss**	
	**(n, 95% UI)**	**rate per 100 children (rate, 95% UI)**	**(n, 95% UI)**	**rate per 100 children (rate, 95% UI)**	**(n, 95% UI)**	**rate per 100 children (rate, 95% UI)**	**(n, 95% UI)**	**rate per 100 children (rate, 95% UI)**	**(n, 95% UI)**	**rate per 100 children (rate, 95% UI)**
Louisiana	2,776	0.26	5,396	0.50	8,172	0.75	1,665	0.15	9,766	0.90
	(2,092, 3,583)	(0.19, 0.33)	(4,382, 6,475)	(0.40, 0.60)	(6,474, 10,058)	(0.60, 0.93)	(1,500,1,837)	(0.14,0.17)	(7,903, 11,804)	(0.73, 1.09)
Maine	384	0.15	995	0.39	1,379	0.55	239	0.09	1,610	0.64
	(208, 642)	(0.08, 0.25)	(636, 1,387)	(0.25, 0.55)	(844, 2,029)	(0.33, 0.81)	(200,289)	(0.08,0.11)	(1,054, 2,305)	(0.42, 0.92)
Maryland	2,427	0.18	5,045	0.37	7,472	0.55	1,654	0.12	9,078	0.67
	(1,808, 3,120)	(0.13, 0.23)	(4,085, 6,105)	(0.30, 0.45)	(5,893, 9,225)	(0.43, 0.68)	(1,502,1,838)	(0.11,0.13)	(7,363, 10,990)	(0.54, 0.81)
Massachusetts	1,895	0.14	4,213	0.31	6,108	0.45	1,394	0.10	7,442	0.55
	(1,404, 2,477)	(0.10, 0.18)	(3,411, 5,094)	(0.25, 0.37)	(4,815, 7,571)	(0.35, 0.56)	(1,286,1,531)	(0.09,0.11)	(6,081, 9,031)	(0.45, 0.66)
Michigan	4,148	0.19	8,108	0.38	12,256	0.57	2,398	0.11	14,558	0.68
	(3,294, 5,098)	(0.15, 0.24)	(6,893, 9,491)	(0.32, 0.44)	(10,186, 14,589)	(0.47, 0.68)	(2,259,2,579)	(0.10,0.12)	(12,367, 17,064)	(0.57, 0.79)
Minnesota	1,739	0.13	3,724	0.28	5,463	0.41	787	0.06	6,232	0.47
	(1,180, 2,374)	(0.09, 0.18)	(2,870, 4,665)	(0.22, 0.35)	(4,050, 7,039)	(0.31, 0.53)	(703,875)	(0.05,0.07)	(4,721, 7,888)	(0.36, 0.60)
Mississippi	1,925	0.28	3,314	0.48	5,239	0.76	1,398	0.20	6,574	0.95
	(1,384, 2,646)	(0.20, 0.38)	(2,521, 4,197)	(0.36, 0.61)	(3,905, 6,843)	(0.56, 0.99)	(1,225,1,572)	(0.18,0.23)	(5,085, 8,329)	(0.73, 1.20)
Missouri	2,777	0.20	5,384	0.39	8,162	0.59	1,668	0.12	9,757	0.70
	(2,084, 3,583)	(0.15, 0.26)	(4,417, 6,470)	(0.32, 0.47)	(6,501, 10,053)	(0.47, 0.73)	(1,538,1,834)	(0.11,0.13)	(7,958, 11,810)	(0.57, 0.85)
Montana	369	0.16	841	0.36	1,210	0.51	204	0.09	1,406	0.60
	(198, 732)	(0.08, 0.31)	(499, 1,283)	(0.21, 0.55)	(697, 2,015)	(0.30, 0.86)	(172,261)	(0.07,0.11)	(866, 2,278)	(0.37, 0.97)
Nebraska	620	0.13	1,185	0.25	1,805	0.37	313	0.06	2,109	0.44
	(354, 1,064)	(0.07, 0.22)	(732, 1,746)	(0.15, 0.36)	(1,086, 2,810)	(0.22, 0.58)	(266,378)	(0.06,0.08)	(1,364, 3,166)	(0.28, 0.66)
Nevada	1,443	0.21	2,611	0.37	4,054	0.58	988	0.14	5,006	0.72
	(948, 2,002)	(0.14, 0.29)	(1,965, 3,397)	(0.28, 0.49)	(2,913, 5,399)	(0.42, 0.77)	(863,1,123)	(0.12,0.16)	(3,766, 6,481)	(0.54, 0.93)
New Hampshire	324	0.13	756	0.30	1,080	0.42	266	0.10	1,349	0.53
	(190, 577)	(0.07, 0.23)	(444, 1,117)	(0.17, 0.44)	(634, 1,694)	(0.25, 0.66)	(210,320)	(0.08,0.12)	(844, 2,013)	(0.33, 0.79)
New Jersey	3,012	0.15	6,782	0.34	9,794	0.48	2,163	0.11	11,892	0.59
	(2,315, 3,786)	(0.11, 0.19)	(5,643, 8,031)	(0.28, 0.40)	(7,958, 11,817)	(0.39, 0.58)	(1,989,2,318)	(0.10,0.11)	(9,874, 14,049)	(0.49, 0.69)
New Mexico	1,451	0.31	2,703	0.57	4,154	0.88	748	0.16	4,871	1.03
	(962, 2,045)	(0.20, 0.43)	(1,989, 3,482)	(0.42, 0.74)	(2,951, 5,527)	(0.62, 1.17)	(639,874)	(0.14,0.18)	(3,570, 6,347)	(0.75, 1.34)
New York	5,963	0.14	13,421	0.33	19,384	0.47	4,810	0.12	24,012	0.58
	(4,956, 7,041)	(0.12, 0.17)	(11,873, 15,074)	(0.29, 0.37)	(16,829, 22,115)	(0.41, 0.54)	(4,566,5,085)	(0.11,0.12)	(21,272, 27,008)	(0.52, 0.66)
North Carolina	5,082	0.22	9,085	0.39	14,166	0.62	2,934	0.13	16,965	0.74
	(4,103, 6,163)	(0.18, 0.27)	(7,815, 10,496)	(0.34, 0.46)	(11,917, 16,660)	(0.52, 0.72)	(2,727,3,156)	(0.12,0.14)	(14,557, 19,659)	(0.63, 0.85)
North Dakota	212	0.11	459	0.25	671	0.36	96	0.05	766	0.41
	(97, 530)	(0.05, 0.29)	(243, 836)	(0.13, 0.45)	(340, 1,366)	(0.18, 0.74)	(67,118)	(0.04,0.06)	(402, 1,479)	(0.22, 0.80)
Ohio	5,597	0.21	10,314	0.40	15,912	0.61	3,218	0.12	18,968	0.73
	(4,590, 6,731)	(0.18, 0.26)	(8,969, 11,793)	(0.34, 0.45)	(13,558, 18,525)	(0.52, 0.71)	(3,031,3,436)	(0.12,0.13)	(16,435, 21,789)	(0.63, 0.84)

See footnotes at the end of table.

Supplementary Table 4: Maternal and paternal orphanood, orphanhood, grandparent caregiver loss and all caregiver loss incidence among US children in 2021 by US state.

LaTeX template

US state[‖]	Maternal Orphanhood[†]		Paternal Orphanhood[‡]		Orphanhood[*]		Grandparent Caregiver Loss		All Caregiver Loss	
	(n, 95% UI)	rate per 100 children (rate, 95% UI)	(n, 95% UI)	rate per 100 children (rate, 95% UI)	(n, 95% UI)	rate per 100 children (rate, 95% UI)	(n, 95% UI)	rate per 100 children (rate, 95% UI)	(n, 95% UI)	rate per 100 children (rate, 95% UI)
	**Incidence in 2021**									
Oklahoma	2,176	0.23	3,966	0.41	6,142	0.64	1,400	0.15	7,473	0.78
	(1,570, 2,942)	(0.16, 0.31)	(3,114, 4,938)	(0.32, 0.51)	(4,684, 7,880)	(0.49, 0.82)	(1,277,1,544)	(0.13,0.16)	(5,886, 9,339)	(0.61, 0.97)
Oregon	1,368	0.16	2,825	0.33	4,193	0.49	932	0.11	5,088	0.59
	(900, 1,866)	(0.10, 0.22)	(2,127, 3,591)	(0.25, 0.42)	(3,027, 5,457)	(0.35, 0.63)	(835,1,024)	(0.10,0.12)	(3,839, 6,454)	(0.45, 0.75)
Pennsylvania	4,896	0.18	9,925	0.37	14,822	0.55	3,404	0.13	18,086	0.68
	(3,990, 5,900)	(0.15, 0.22)	(8,530, 11,358)	(0.32, 0.42)	(12,520, 17,258)	(0.47, 0.65)	(3,210,3,645)	(0.12,0.14)	(15,606, 20,721)	(0.58, 0.77)
Rhode Island	238	0.11	553	0.26	791	0.38	244	0.12	1,035	0.50
	(132, 419)	(0.06, 0.20)	(310, 827)	(0.15, 0.40)	(442, 1,246)	(0.21, 0.60)	(203,307)	(0.10,0.15)	(652, 1,550)	(0.31, 0.74)
South Carolina	2,798	0.25	4,522	0.40	7,321	0.66	1,802	0.16	9,040	0.81
	(2,100, 3,610)	(0.19, 0.32)	(3,649, 5,467)	(0.33, 0.49)	(5,749, 9,077)	(0.51, 0.81)	(1,646,1,980)	(0.15,0.18)	(7,332, 10,996)	(0.66, 0.98)
South Dakota	347	0.16	727	0.33	1,074	0.49	150	0.07	1,221	0.55
	(194, 726)	(0.09, 0.33)	(394, 1,237)	(0.18, 0.56)	(588, 1,963)	(0.27, 0.89)	(121,183)	(0.05,0.08)	(710, 2,150)	(0.32, 0.98)
Tennessee	4,236	0.27	7,770	0.50	12,006	0.78	2,576	0.17	14,448	0.94
	(3,356, 5,246)	(0.22, 0.34)	(6,541, 9,035)	(0.42, 0.59)	(9,896, 14,281)	(0.64, 0.93)	(2,360,2,798)	(0.15,0.18)	(12,152, 16,928)	(0.79, 1.10)
Texas	13,517	0.18	27,002	0.36	40,518	0.54	9,012	0.12	49,154	0.66
	(11,811, 15,339)	(0.16, 0.21)	(24,592, 29,492)	(0.33, 0.39)	(36,402, 44,832)	(0.49, 0.60)	(8,626,9,484)	(0.12,0.13)	(44,680, 53,910)	(0.60, 0.72)
Utah	1,328	0.14	2,443	0.26	3,771	0.40	666	0.07	4,420	0.47
	(843, 1,984)	(0.09, 0.21)	(1,742, 3,287)	(0.18, 0.35)	(2,585, 5,271)	(0.27, 0.56)	(554,760)	(0.06,0.08)	(3,122, 6,021)	(0.33, 0.64)
Vermont	104	0.09	366	0.31	470	0.40	92	0.08	560	0.48
	(35, 243)	(0.03, 0.21)	(194, 581)	(0.17, 0.50)	(229, 824)	(0.20, 0.70)	(63,123)	(0.05,0.11)	(292, 945)	(0.25, 0.81)
Virginia	3,334	0.18	6,379	0.34	9,712	0.52	2,221	0.12	11,844	0.63
	(2,599, 4,189)	(0.14, 0.22)	(5,303, 7,593)	(0.28, 0.40)	(7,902, 11,782)	(0.42, 0.63)	(2,064,2,423)	(0.11,0.13)	(9,904, 14,087)	(0.53, 0.75)
Washington	2,502	0.15	4,989	0.30	7,492	0.45	1,563	0.09	8,985	0.54
	(1,829, 3,264)	(0.11, 0.19)	(4,023, 6,025)	(0.24, 0.36)	(5,852, 9,289)	(0.35, 0.55)	(1,443,1,699)	(0.09,0.10)	(7,252, 10,924)	(0.43, 0.65)
West Virginia	1,042	0.29	2,134	0.59	3,176	0.88	831	0.23	3,958	1.10
	(695, 1,514)	(0.19, 0.42)	(1,558, 2,738)	(0.43, 0.76)	(2,253, 4,252)	(0.63, 1.18)	(732,952)	(0.20,0.27)	(2,946, 5,153)	(0.82, 1.44)
Wisconsin	1,910	0.15	3,036	0.24	4,946	0.39	894	0.07	5,805	0.46
	(1,386, 2,586)	(0.11, 0.20)	(2,393, 3,747)	(0.19, 0.29)	(3,779, 6,333)	(0.30, 0.50)	(821,985)	(0.06,0.08)	(4,593, 7,277)	(0.36, 0.57)
Wyoming	155	0.12	387	0.29	542	0.41	116	0.09	655	0.49
	(74, 432)	(0.06, 0.33)	(176, 664)	(0.13, 0.50)	(250, 1,096)	(0.19, 0.83)	(87,151)	(0.07,0.11)	(336, 1,249)	(0.25, 0.94)

[†]: Maternal orphanhood – "Children experiencing the death of their mother, including children experiencing the death of both their mother and father; Eq.(9) "

[‡]: Paternal orphanhood – "Children experiencing the death of their father, including children experiencing the death of both their father and mother; Eq.(9) "

[*]: Orphanhood – "Children experiencing the death of one or both parents; Eq.(7) "

[‖]: Discrepancies between the sum of US state-level estimates and the national-level estimates in Table 2 are due to working from partially suppressed counts from CDC WONDER (see Methods).

Supplementary Table 4: Maternal and paternal orphanood, orphanhood, grandparent caregiver loss and all caregiver loss incidence among US children in 2021 by US state (continued).

Prevalence in 2021										
**US state**[‖]	**Maternal Orphanhood**[†]		**Paternal Orphanhood**[‡]		**Orphanhood***		**Grandparent Caregiver Loss**		**All Caregiver Loss**	
	**(n, 95% UI)**	**rate per 100 children (rate, 95% UI)**	**(n, 95% UI)**	**rate per 100 children (rate, 95% UI)**	**(n, 95% UI)**	**rate per 100 children (rate, 95% UI)**	**(n, 95% UI)**	**rate per 100 children (rate, 95% UI)**	**(n, 95% UI)**	**rate per 100 children (rate, 95% UI)**
United States	789,423	1.14	1,588,843	2.29	2,378,250	3.43	559,712	0.81	2,912,817	4.20
	(691,479, 903,061)	(1.00, 1.30)	(1,456,757, 1,735,178)	(2.10, 2.50)	(2,148,223, 2,638,221)	(3.10, 3.80)	(530,597, 590,353)	(0.76, 0.85)	(2,654,936, 3,202,040)	(3.83 , 4.61)
Alabama	15,947	1.42	28,894	2.57	44,842	4.00	11,846	1.06	56,128	5.00
	(11,333, 21,851)	(1.01, 1.95)	(22,416, 36,189)	(2.00, 3.22)	(33,750, 58,038)	(3.01, 5.17)	(10,644,13,148)	(0.95,1.17)	(43,885, 70,708)	(3.91, 6.30)
Alaska	1,934	1.08	3,853	2.15	5,785	3.23	839	0.47	6,622	3.69
	(905, 5,220)	(0.50, 2.91)	(2,003, 6,886)	(1.12, 3.84)	(2,906, 12,106)	(1.62, 6.75)	(584,1,222)	(0.33,0.68)	(3,498, 13,313)	(1.95, 7.42)
Arizona	19,584	1.21	39,012	2.42	58,592	3.63	12,992	0.80	71,124	4.41
	(13,986, 26,609)	(0.87, 1.65)	(30,890, 48,074)	(1.91, 2.98)	(44,874, 74,683)	(2.78, 4.63)	(11,719,14,524)	(0.73,0.90)	(56,156, 88,700)	(3.48, 5.50)
Arkansas	8,886	1.26	16,361	2.33	25,246	3.59	6,834	0.97	31,776	4.52
	(5,681, 13,512)	(0.81, 1.92)	(11,613, 21,889)	(1.65, 3.11)	(17,292, 35,399)	(2.46, 5.03)	(5,959,7,819)	(0.85,1.11)	(22,955, 42,794)	(3.26, 6.08)
California	79,256	0.90	184,216	2.10	263,472	3.00	72,999	0.83	333,656	3.80
	(67,698, 91,717)	(0.77, 1.05)	(166,097, 203,079)	(1.89, 2.31)	(233,794, 294,797)	(2.67, 3.36)	(69,603,76,932)	(0.79,0.88)	(300,750, 368,940)	(3.43, 4.21)
Colorado	12,357	0.99	25,378	2.04	37,738	3.03	6,312	0.51	43,846	3.53
	(8,321, 17,681)	(0.67, 1.42)	(19,078, 32,397)	(1.53, 2.61)	(27,398, 50,079)	(2.20, 4.03)	(5,491,7,108)	(0.44,0.57)	(32,714, 56,996)	(2.63, 4.58)
Connecticut	6,144	0.84	14,717	2.02	20,860	2.86	4,892	0.67	25,628	3.51
	(3,753, 9,390)	(0.51, 1.29)	(10,398, 19,677)	(1.42, 2.70)	(14,148, 29,067)	(1.94, 3.98)	(4,185,5,598)	(0.57,0.77)	(18,227, 34,553)	(2.50, 4.74)
Delaware	1,906	0.92	3,454	1.66	5,361	2.57	1,667	0.80	7,018	3.37
	(856, 4,448)	(0.41, 2.14)	(1,775, 5,463)	(0.85, 2.62)	(2,630, 9,911)	(1.26, 4.76)	(1,280,2,217)	(0.61,1.06)	(3,937, 12,109)	(1.89, 5.81)
District of Columbia	1,119	0.89	2,686	2.13	3,806	3.02	667	0.53	4,466	3.55
	(463, 2,109)	(0.37, 1.68)	(1,284, 4,354)	(1.02, 3.46)	(1,747, 6,465)	(1.39, 5.14)	(424,875)	(0.34,0.70)	(2,161, 7,340)	(1.72, 5.83)
Florida	52,749	1.23	105,821	2.47	158,570	3.70	40,348	0.94	197,252	4.60
	(43,588, 62,906)	(1.02, 1.47)	(93,139, 119,397)	(2.17, 2.78)	(136,729, 182,303)	(3.19, 4.25)	(38,174,42,874)	(0.89,1.00)	(173,299, 223,548)	(4.04, 5.21)
Georgia	30,438	1.21	56,203	2.23	86,644	3.43	20,024	0.79	105,780	4.19
	(23,472, 38,437)	(0.93, 1.52)	(46,435, 66,784)	(1.84, 2.65)	(69,907, 105,222)	(2.77, 4.17)	(18,320,21,859)	(0.73,0.87)	(87,369, 125,999)	(3.46, 4.99)
Hawaii	1,952	0.64	5,620	1.85	7,572	2.49	3,651	1.20	11,146	3.66
	(986, 4,656)	(0.32, 1.53)	(3,068, 8,638)	(1.01, 2.84)	(4,053, 13,296)	(1.33, 4.37)	(2,792,4,565)	(0.92,1.50)	(6,808, 17,791)	(2.24, 5.84)
Idaho	3,360	0.72	7,013	1.50	10,372	2.21	2,042	0.44	12,397	2.64
	(1,580, 6,760)	(0.34, 1.44)	(4,063, 11,379)	(0.87, 2.43)	(5,642, 18,139)	(1.20, 3.87)	(1,617,2,633)	(0.34,0.56)	(7,253, 20,762)	(1.55, 4.43)
Illinois	28,210	1.01	61,226	2.18	89,434	3.19	20,680	0.74	109,292	3.90
	(21,669, 35,742)	(0.77, 1.28)	(50,877, 72,293)	(1.81, 2.58)	(72,544, 108,037)	(2.59, 3.85)	(19,110,22,402)	(0.68,0.80)	(90,958, 129,649)	(3.24, 4.62)
Indiana	18,472	1.16	35,882	2.26	54,354	3.42	11,218	0.71	65,076	4.10
	(13,241, 24,866)	(0.83, 1.57)	(28,316, 44,462)	(1.78, 2.80)	(41,559, 69,327)	(2.62, 4.37)	(10,180,12,327)	(0.64,0.78)	(51,197, 81,109)	(3.23, 5.11)
Iowa	5,398	0.73	11,092	1.51	16,491	2.24	3,366	0.46	19,770	2.68
	(3,081, 9,182)	(0.42, 1.25)	(7,443, 15,819)	(1.01, 2.15)	(10,524, 25,000)	(1.43, 3.39)	(2,878,3,856)	(0.39,0.52)	(13,333, 28,766)	(1.81, 3.91)
Kansas	6,446	0.92	13,260	1.89	19,704	2.80	4,028	0.57	23,618	3.36
	(3,779, 10,761)	(0.54, 1.53)	(8,931, 18,662)	(1.27, 2.65)	(12,710, 29,423)	(1.81, 4.18)	(3,501,4,683)	(0.50,0.67)	(16,120, 33,957)	(2.29, 4.83)
Kentucky	14,737	1.45	26,919	2.65	41,655	4.10	10,063	0.99	51,258	5.05
	(10,494, 20,368)	(1.03, 2.00)	(20,856, 33,781)	(2.05, 3.33)	(31,353, 54,148)	(3.09, 5.33)	(9,005,11,161)	(0.89,1.10)	(39,924, 64,686)	(3.93, 6.37)

See footnotes at the end of table.

Supplementary Table 5: Maternal and paternal orphanood, orphanhood, grandparent caregiver loss and all caregiver loss prevalence among US children in 2021 by US state.

LaTeX template

US state[‖]	Maternal Orphanhood[†] (n, 95% UI)	Maternal Orphanhood[†] rate per 100 children (rate, 95% UI)	Paternal Orphanhood[‡] (n, 95% UI)	Paternal Orphanhood[‡] rate per 100 children (rate, 95% UI)	Orphanhood[*] (n, 95% UI)	Orphanhood[*] rate per 100 children (rate, 95% UI)	Grandparent Caregiver Loss (n, 95% UI)	Grandparent Caregiver Loss rate per 100 children (rate, 95% UI)	All Caregiver Loss (n, 95% UI)	All Caregiver Loss rate per 100 children (rate, 95% UI)
	**Prevalence in 2021**									
Louisiana	15,368	1.42	30,588	2.82	45,957	4.24	10,518	0.97	55,997	5.17
	(10,871, 21,081)	(1.00, 1.95)	(23,711, 38,433)	(2.19, 3.55)	(34,581, 59,512)	(3.19, 5.50)	(9,264,11,890)	(0.86,1.10)	(43,471, 70,889)	(4.01, 6.55)
Maine	1,922	0.76	5,314	2.11	7,236	2.87	1,482	0.59	8,712	3.46
	(863, 3,963)	(0.34, 1.57)	(2,923, 8,017)	(1.16, 3.18)	(3,784, 11,981)	(1.50, 4.76)	(1,181,1,844)	(0.47,0.73)	(4,977, 13,833)	(1.98, 5.49)
Maryland	15,060	1.10	32,801	2.41	47,860	3.51	10,806	0.79	58,285	4.28
	(10,799, 20,345)	(0.79, 1.49)	(25,616, 40,714)	(1.88, 2.99)	(36,414, 61,059)	(2.67, 4.48)	(9,652,12,172)	(0.71,0.89)	(45,738, 72,785)	(3.35, 5.34)
Massachusetts	12,462	0.91	28,872	2.12	41,334	3.03	9,500	0.70	50,488	3.71
	(8,681, 16,975)	(0.64, 1.25)	(22,676, 35,833)	(1.66, 2.63)	(31,355, 52,807)	(2.30, 3.88)	(8,556,10,517)	(0.63,0.77)	(39,617, 62,955)	(2.91, 4.62)
Michigan	25,144	1.17	49,459	2.30	74,604	3.46	15,224	0.71	89,224	4.14
	(19,053, 32,316)	(0.88, 1.50)	(40,438, 59,275)	(1.88, 2.75)	(59,491, 91,589)	(2.76, 4.25)	(14,016,16,602)	(0.65,0.77)	(72,960, 107,546)	(3.39, 4.99)
Minnesota	10,066	0.76	20,334	1.54	30,401	2.31	4,937	0.37	35,214	2.67
	(6,454, 14,899)	(0.49, 1.13)	(14,920, 26,790)	(1.13, 2.03)	(21,374, 41,688)	(1.62, 3.16)	(4,407,5,478)	(0.33,0.42)	(25,650, 47,018)	(1.95, 3.57)
Mississippi	10,194	1.47	19,420	2.80	29,612	4.27	8,584	1.24	37,814	5.46
	(6,917, 15,039)	(1.00, 2.17)	(14,133, 25,513)	(2.04, 3.68)	(21,050, 40,550)	(3.04, 5.85)	(7,497,9,863)	(1.08,1.42)	(28,217, 49,960)	(4.07, 7.21)
Missouri	16,653	1.20	32,402	2.34	49,053	3.54	10,280	0.74	58,895	4.25
	(11,741, 22,659)	(0.85, 1.64)	(25,448, 40,304)	(1.84, 2.91)	(37,188, 62,962)	(2.69, 4.55)	(9,287,11,420)	(0.67,0.82)	(46,101, 73,891)	(3.33, 5.34)
Montana	1,929	0.82	4,457	1.90	6,387	2.72	1,113	0.47	7,498	3.19
	(857, 4,778)	(0.36, 2.03)	(2,380, 7,767)	(1.01, 3.30)	(3,236, 12,544)	(1.38, 5.34)	(890,1,572)	(0.38,0.67)	(4,122, 14,111)	(1.75, 6.00)
Nebraska	3,367	0.70	7,150	1.48	10,511	2.18	2,028	0.42	12,508	2.59
	(1,756, 6,708)	(0.36, 1.39)	(4,284, 11,413)	(0.89, 2.36)	(6,037, 18,121)	(1.25, 3.75)	(1,693,2,582)	(0.35,0.53)	(7,710, 20,668)	(1.60, 4.28)
Nevada	7,464	1.07	14,281	2.04	21,742	3.11	5,360	0.77	26,966	3.86
	(4,445, 11,726)	(0.64, 1.68)	(9,972, 19,326)	(1.43, 2.77)	(14,418, 31,050)	(2.06, 4.44)	(4,488,6,333)	(0.64,0.91)	(18,820, 37,175)	(2.69, 5.32)
New Hampshire	1,943	0.76	4,850	1.89	6,792	2.65	1,506	0.59	8,298	3.24
	(967, 4,101)	(0.38, 1.60)	(2,618, 7,621)	(1.02, 2.97)	(3,587, 11,720)	(1.40, 4.57)	(1,171,1,977)	(0.46,0.77)	(4,749, 13,687)	(1.85, 5.34)
New Jersey	19,178	0.95	44,828	2.22	64,008	3.16	14,960	0.74	78,438	3.88
	(14,240, 25,187)	(0.70, 1.24)	(36,086, 54,278)	(1.78, 2.68)	(50,326, 79,466)	(2.49, 3.93)	(13,579,16,304)	(0.67,0.81)	(63,424, 95,340)	(3.13, 4.71)
New Mexico	7,154	1.51	14,263	3.01	21,418	4.53	4,240	0.90	25,534	5.40
	(4,353, 11,443)	(0.92, 2.42)	(9,641, 19,565)	(2.04, 4.13)	(13,994, 31,009)	(2.96, 6.55)	(3,373,5,171)	(0.71,1.09)	(17,233, 36,013)	(3.64, 7.61)
New York	37,510	0.91	86,696	2.11	124,204	3.02	32,214	0.78	155,214	3.77
	(30,358, 45,651)	(0.74, 1.11)	(75,244, 98,973)	(1.83, 2.41)	(105,602, 144,622)	(2.57, 3.52)	(30,298,34,463)	(0.74,0.84)	(134,708, 177,788)	(3.27, 4.32)
North Carolina	27,526	1.20	50,738	2.20	78,268	3.40	17,484	0.76	94,938	4.13
	(21,050, 35,034)	(0.91, 1.52)	(41,836, 60,537)	(1.82, 2.63)	(62,890, 95,571)	(2.73, 4.15)	(16,105,19,114)	(0.70,0.83)	(78,291, 113,859)	(3.40, 4.95)
North Dakota	1,077	0.58	2,670	1.44	3,750	2.02	523	0.28	4,270	2.30
	(422, 3,245)	(0.23, 1.75)	(1,340, 5,547)	(0.72, 2.99)	(1,760, 8,793)	(0.95, 4.74)	(358,787)	(0.19,0.42)	(2,125, 9,557)	(1.14, 5.15)
Ohio	33,654	1.29	62,800	2.41	96,450	3.70	20,090	0.77	115,586	4.44
	(26,510, 41,790)	(1.02, 1.60)	(52,961, 73,594)	(2.03, 2.82)	(79,472, 115,385)	(3.05, 4.43)	(18,668,21,690)	(0.72,0.83)	(97,305, 136,206)	(3.73, 5.23)

See footnotes at the end of table.

Supplementary Table 5: Maternal and paternal orphanood, orphanhood, grandparent caregiver loss and all caregiver loss prevalence among US children in 2021 by US state.

Prevalence in 2021										
US state‖	Maternal Orphanhood†		Paternal Orphanhood‡		Orphanhood*		Grandparent Caregiver Loss		All Caregiver Loss	
	(n, 95% UI)	rate per 100 children (rate, 95% UI)	(n, 95% UI)	rate per 100 children (rate, 95% UI)	(n, 95% UI)	rate per 100 children (rate, 95% UI)	(n, 95% UI)	rate per 100 children (rate, 95% UI)	(n, 95% UI)	rate per 100 children (rate, 95% UI)
Oklahoma	12,178	1.27	23,599	2.45	35,776	3.72	8,370	0.87	43,768	4.55
	(8,139, 17,714)	(0.85, 1.84)	(17,463, 30,637)	(1.82, 3.19)	(25,602, 48,352)	(2.66, 5.03)	(7,320,9,451)	(0.76,0.98)	(32,598, 57,387)	(3.39, 5.97)
Oregon	7,384	0.86	15,479	1.80	22,862	2.65	5,326	0.62	28,018	3.25
	(4,444, 11,159)	(0.52, 1.30)	(11,066, 20,530)	(1.28, 2.38)	(15,511, 31,690)	(1.80, 3.68)	(4,607,6,075)	(0.53,0.71)	(19,922, 37,607)	(2.31, 4.37)
Pennsylvania	30,453	1.14	64,870	2.43	95,322	3.56	21,828	0.82	116,259	4.35
	(24,048, 37,988)	(0.90, 1.42)	(54,533, 75,814)	(2.04, 2.84)	(78,581, 113,803)	(2.94, 4.26)	(20,356,23,475)	(0.76,0.88)	(98,120, 136,371)	(3.67, 5.10)
Rhode Island	1,226	0.59	3,565	1.71	4,792	2.29	1,522	0.73	6,311	3.02
	(507, 2,588)	(0.24, 1.24)	(1,749, 5,769)	(0.84, 2.76)	(2,256, 8,358)	(1.08, 4.00)	(1,166,1,986)	(0.56,0.95)	(3,405, 10,346)	(1.63, 4.95)
South Carolina	14,462	1.29	24,626	2.20	39,090	3.50	10,478	0.94	49,133	4.40
	(10,239, 19,938)	(0.92, 1.78)	(18,961, 30,941)	(1.70, 2.77)	(29,200, 50,878)	(2.61, 4.55)	(9,345,11,736)	(0.84,1.05)	(38,104, 62,079)	(3.41, 5.56)
South Dakota	1,623	0.74	3,764	1.71	5,385	2.44	892	0.40	6,278	2.85
	(758, 4,548)	(0.34, 2.06)	(1,867, 7,442)	(0.85, 3.38)	(2,624, 11,990)	(1.19, 5.44)	(670,1,286)	(0.30,0.58)	(3,292, 13,277)	(1.49, 6.02)
Tennessee	21,830	1.42	40,828	2.65	62,657	4.07	14,828	0.96	76,784	4.98
	(16,312, 28,600)	(1.06, 1.86)	(32,815, 49,698)	(2.13, 3.23)	(49,128, 78,298)	(3.19, 5.08)	(13,433,16,303)	(0.87,1.06)	(61,938, 93,874)	(4.02, 6.09)
Texas	73,706	0.99	148,370	1.98	222,077	2.97	53,736	0.72	273,540	3.66
	(62,002, 86,480)	(0.83, 1.16)	(131,878, 165,808)	(1.76, 2.22)	(193,884, 252,286)	(2.59, 3.37)	(50,836,56,968)	(0.68,0.76)	(242,341, 306,853)	(3.24, 4.10)
Utah	8,334	0.88	16,017	1.69	24,352	2.57	4,064	0.43	28,338	2.99
	(4,932, 13,484)	(0.52, 1.42)	(10,840, 22,487)	(1.14, 2.37)	(15,774, 35,974)	(1.67, 3.80)	(3,204,4,816)	(0.34,0.51)	(18,886, 40,717)	(1.99, 4.30)
Vermont	576	0.49	1,793	1.53	2,368	2.02	530	0.45	2,898	2.48
	(152, 1,686)	(0.13, 1.44)	(667, 3,248)	(0.57, 2.78)	(818, 4,934)	(0.70, 4.22)	(304,798)	(0.26,0.68)	(1,116, 5,730)	(0.95, 4.90)
Virginia	19,117	1.01	37,544	1.99	56,662	3.01	13,836	0.73	69,940	3.71
	(14,028, 25,346)	(0.74, 1.34)	(29,929, 46,071)	(1.59, 2.44)	(43,957, 71,417)	(2.33, 3.79)	(12,556,15,280)	(0.67,0.81)	(56,025, 86,043)	(2.97, 4.57)
Washington	14,190	0.85	28,438	1.70	42,628	2.54	9,104	0.54	51,380	3.07
	(9,886, 19,707)	(0.59, 1.18)	(21,730, 35,772)	(1.30, 2.13)	(31,616, 55,479)	(1.89, 3.31)	(8,169,10,097)	(0.49,0.60)	(39,446, 65,205)	(2.35, 3.89)
West Virginia	5,706	1.59	12,048	3.36	17,754	4.94	4,571	1.27	22,136	6.17
	(3,467, 9,184)	(0.97, 2.56)	(8,312, 16,393)	(2.32, 4.57)	(11,778, 25,574)	(3.28, 7.12)	(3,821,5,372)	(1.06,1.50)	(15,491, 30,731)	(4.31, 8.56)
Wisconsin	11,368	0.89	18,221	1.43	29,588	2.32	5,716	0.45	35,111	2.75
	(7,695, 16,350)	(0.60, 1.28)	(13,661, 23,282)	(1.07, 1.83)	(21,354, 39,631)	(1.68, 3.11)	(5,144,6,401)	(0.40,0.50)	(26,344, 45,864)	(2.07, 3.60)
Wyoming	837	0.63	2,159	1.63	2,994	2.26	582	0.44	3,577	2.70
	(292, 2,994)	(0.22, 2.26)	(852, 4,298)	(0.64, 3.25)	(1,143, 7,293)	(0.86, 5.51)	(375,839)	(0.28,0.63)	(1,516, 8,132)	(1.15, 6.14)

†: Maternal orphanhood – "Children experiencing the death of their mother, including children experiencing the death of both their mother and father; Eq. (9)"

‡: Paternal orphanhood – "Children experiencing the death of their father, including children experiencing the death of both their father and mother; Eq. (9)"

*: Orphanhood – "Children experiencing the death of one or both parents; Eq. (7)"

‖: Discrepancies between the sum of US state-level estimates and the national-level estimates in Table 2 are due to working from partially suppressed counts from CDC WONDER (see Methods).

Supplementary Table 5: Maternal and paternal orphanood, orphanhood, grandparent caregiver loss and all caregiver loss prevalence among US children in 2021 by US state (continued).

LaTeX template

standardized race categories	Time period (years)<br>1984-1991	1992-2003	2004-2020	2021
American Indian<br>or Alaska Native	American Indian<br>(includes Aleuts and Eskimos)	American Indian<br>(includes Aleuts and Eskimos)	American Indian	American Indian<br>or Alaskan Native
Asian<br>or Pacific Islander	Chinese; Japanese; Filipino;<br>Hawaiian (includes Part-Hawaiian);<br>Other Asian or Pacific Islander	Chinese; Japanese; Filipino;<br>Hawaiian (includes Part-Hawaiian);<br>Asian Indian; Korean; Samoan;<br>Vietnamese; Guamanian;<br>Other Asian or Pacific Islander in areas;<br>Combined other Asian or Pacific Islander	Asian<br>or Pacific Islander	Chinese; Japanese; Filipino;<br>Hawaiian; Asian Indian;<br>Korean; Samoan;<br>Vietnamese; Guamanian;<br>Other or Multiple Asian;<br>Other or Multiple Pacific Islander
Black<br>or African American	Black	Black	Black	Black
White	White	White	White	White
Not included in study†	Other races	-	-	More than one race
**Ethnicity**				
Hispanic origin	Spaniard; Mexican; Puerto Rican; Cuban; Dominican; Central or South American; Central American; South American; Latin American; Other and Unknown Spanish; Other Hispanic			

†: Individuals of other races in 1984-1991 and individuals of more than one race in 2021 were not coded consistently and not included in this study, which corresponded to 0.0068% of all death records in 1983-1991 and 0.47% in 2021.

Supplementary Table 6: Mapping of single-race mortality data to standardized race categories and Hispanic origin.

standardized race categories	Time period (years)<br>1969-1977	1978-1988	1989-1991	1992-2002	2003-2016	2017-2021
American Indian<br>or Alaska Native	American Indian<br>(includes Aleuts and Eskimos)	American Indian<br>(includes Aleuts and Eskimos)	American Indian<br>(includes Aleuts and Eskimos)	American Indian<br>(includes Aleuts and Eskimos)	American Indian	American Indian
Asian<br>or Pacific Islander	Chinese; Japanese; Filipino;<br>Guamian; Hawaiian<br>(includes Part-Hawaiian)	Chinese; Japanese; Filipino;<br>Hawaiian<br>(includes Part-Hawaiian)<br>Other Asian<br>or Pacific Islander in areas	Chinese; Japanese; Filipino;<br>Hawaiian<br>(includes Part-Hawaiian)<br>Other Asian<br>or Pacific Islander in areas	Chinese; Japanese; Filipino;<br>Hawaiian<br>(includes Part-Hawaiian);<br>Asian Indian; Korean; Samoan;<br>Vietnamese; Guamanian;<br>Other Asian<br>or Pacific Islander in areas	Asian<br>or Pacific Islander	Asian;<br>Native Hawaiian or<br>Other Pacific Islander
Black<br>or African American	Negro	Black	Black	Black	Black	Black
White	White	White	White	White	White	White
Not included in study†	Other non-White<br>Not stated	Other non-White<br>Not stated	Other races<br>Not stated	More than one race<br>Not stated	-<br>Not stated	More than one race<br>Not stated
**Ethnicity**	**1978-2021**					
Hispanic origin	Mexican; Puerto Rican; Cuban; Dominican; Central or South American; Other and Unknown Spanish					

†: Individuals of other non-White; note stated; other races in 1969-1991 and 2003-2016 and individuals of more than one race or note stated race in 1992-2002 and 2017-2021 were not coded consistently and not included in this study.

Supplementary Table 7: Mapping of single-race live birth data to standardized race categories and Hispanic origin.

LaTeX template

**standardized race categories**	**Time period (years)** 1990-2020	2021
American Indian or Alaska Native	American Indian or Alaska Native	American Indian or Alaska Native
Asian or Pacific Islander	Asian or Pacific Islander	Asian
		Native Hawaiian or Other Pacific Islander
Black or African American	Black or African American	Black or African American
White	White	White
Not included in study†		More than one race
**Ethnicity**	**1978-2021**	
Hispanic origin	Hispanic or Latino	

†: Individuals of more than one race in 2021 were not coded consistently and not included in this study.

Supplementary Table 8: Mapping of single-race population size data to standardized race categories and Hispanic origin.

	Before the COVID-19 pandemic			Since the COVID-19 pandemic			
	2000 (Number of Children)	2019 (Number of Children)	2000-2019 changes	2020 (Number of Children)	2021 (Number of Children)	2019-2021 changes	2000-2021 changes
**Incidence (n, (95% uncertainty interval))**							
**Total**	262,036 (237,122, 290,407)	292,118 (265,553, 321,908)	+11.5% (+10.9%, +12.0%)	360,192 (329,378, 394,370)	407,377 (374,905, 442,874)	+39.4% (+37.6%, +41.2%)	+55.5% (+52.5%, +58.1%)
**Ages 0-4 years**	45,495 (40,357, 51,509)	54,034 (48,535, 60,284)	+18.8% (+17.0%, +20.3%)	66,077 (59,762, 73,150)	72,937 (66,366, 80,145)	+35.0% (+32.9%, +36.7%)	+60.3% (+55.6%, +64.4%)
**Ages 5-9 years**	61,911 (55,496, 69,290)	69,823 (63,015, 77,449)	+12.8% (+11.8%, +13.5%)	87,129 (79,166, 95,940)	99,829 (91,380, 109,050)	+42.9% (+40.8%, +45.0%)	+61.2% (+57.4%, +64.7%)
**Ages 10-17 years**	154,630 (141,269, 169,608)	168,261 (154,003, 184,175)	+8.8% (+8.6%, +9.0%)	206,986 (190,450, 225,280)	234,611 (217,159, 253,679)	+39.4% (+37.7%, +41.0%)	+51.7% (+49.6%, +53.7%)
**Incidence rate per 100 children (rate, (95% uncertainty interval))**							
**Total**	0.36 (0.33, 0.40)	0.40 (0.36, 0.44)	+10.4% (+9.8%, +10.9%)	0.49 (0.45, 0.54)	0.59 (0.54, 0.64)	+46.9% (+44.9%, +48.7%)	+62.1% (+59.1%, +64.9%)
**Ages 0-4 years**	0.24 (0.21, 0.27)	0.28 (0.25, 0.31)	+16.4% (+14.7%, +17.8%)	0.34 (0.31, 0.38)	0.41 (0.37, 0.45)	+49.2% (+46.9%, +51.1%)	+73.7% (+68.6%, +78.1%)
**Ages 5-9 years**	0.30 (0.27, 0.34)	0.35 (0.31, 0.38)	+14.1% (+13.1%, +14.9%)	0.43 (0.39, 0.47)	0.52 (0.48, 0.57)	+51.6% (+49.4%, +53.8%)	+73.0% (+68.9%, +76.7%)
**Ages 10-17 years**	0.47 (0.43, 0.52)	0.51 (0.46, 0.55)	+7.0% (+6.8%, +7.2%)	0.62 (0.57, 0.68)	0.72 (0.67, 0.78)	+42.3% (+40.6%, +43.9%)	+52.2% (+50.1%, +54.2%)
**Incidence rate ratio (ratio relative to children aged 0-4; 95% UI)**							
**Ages 0-4 years**	1.00 (1.00, 1.00)	1.00 (1.00, 1.00)	-	1.00 (1.00, 1.00)	1.00 (1.00, 1.00)	-	-
**Ages 5-9 years**	1.28 (1.26, 1.29)	1.25 (1.24, 1.26)	-	1.26 (1.25, 1.26)	1.27 (1.26, 1.28)	-	-
**Ages 10-17 years**	1.99 (1.93, 2.05)	1.83 (1.80, 1.87)	-	1.82 (1.79, 1.85)	1.75 (1.72, 1.78)	-	-
**Prevalence (n, (95 uncertainty interval))**							
**Total**	2,220,606 (2,001,053, 2,472,064)	2,159,537 (1,941,498, 2,408,054)	-2.7% (-3.0%, -2.6%)	2,251,322 (2,027,683, 2,505,227)	2,378,250 (2,148,223, 2,638,221)	+10.1% (+9.6%, +10.6%)	+7.1% (+6.7%, +7.4%)
**Ages 0-4 years**	131,319 (116,251, 148,867)	156,399 (140,220, 174,788)	+19.1% (+17.4%, +20.6%)	168,258 (151,403, 187,337)	183,052 (165,511, 202,691)	+17.0% (+16.0%, +18.0%)	+39.4% (+36.2%, +42.4%)
**Ages 5-9 years**	441,375 (394,679, 495,407)	436,193 (390,167, 488,974)	-1.2% (-1.3%, -1.1%)	464,081 (416,366, 518,312)	503,279 (453,518, 559,484)	+15.4% (+14.4%, +16.2%)	+14.0% (+12.9%, +14.9%)
**Ages 10-17 years**	1,647,912 (1,490,123, 1,827,790)	1,566,944 (1,411,111, 1,744,292)	-4.9% (-5.3%, -4.6%)	1,618,982 (1,459,914, 1,799,578)	1,691,918 (1,529,195, 1,876,046)	+8.0% (+7.6%, +8.4%)	+2.7% (+2.6%, +2.7%)
**Prevalence rate per 100 children (rate, (95% uncertainty interval))**							
**Total**	3.07 (2.76, 3.42)	2.95 (2.66, 3.29)	-3.70% (-3.92%, -3.54%)	3.09 (2.78, 3.44)	3.43 (3.10, 3.80)	+15.99% (+15.39%, +16.54%)	+11.70% (+11.31%, +11.97%)
**Ages 0-4 years**	0.68 (0.61, 0.78)	0.80 (0.72, 0.89)	+16.71% (+15.05%, +18.19%)	0.87 (0.78, 0.97)	1.03 (0.93, 1.14)	+29.38% (+28.19%, +30.48%)	+51.00% (+47.49%, +54.21%)
**Ages 5-9 years**	2.16 (1.93, 2.42)	2.16 (1.93, 2.42)	+0.02% (-0.13%, +0.08%)	2.29 (2.06, 2.56)	2.64 (2.38, 2.93)	+22.35% (+21.34%, +23.27%)	+22.37% (+21.20%, +23.32%)
**Ages 10-17 years**	5.03 (4.55, 5.58)	4.71 (4.24, 5.24)	-6.52% (-6.90%, -6.18%)	4.86 (4.39, 5.41)	5.19 (4.69, 5.75)	+10.20% (+9.77%, +10.60%)	+3.02% (+2.96%, +3.05%)
**Prevalence rate ratio (ratio relative to children aged 0-4; 95% UI)**							
**Ages 0-4 years**	1.00 (1.00, 1.00)	1.00 (1.00, 1.00)	-	1.00 (1.00, 1.00)	1.00 (1.00, 1.00)	-	-
**Ages 5-9 years**	3.15 (3.12, 3.18)	2.70 (2.69, 2.71)	-	2.63 (2.62, 2.64)	2.55 (2.54, 2.56)	-	-
**Ages 10-17 years**	7.35 (7.19, 7.51)	5.89 (5.87, 5.92)	-	5.58 (5.57, 5.59)	5.02 (5.01, 5.02)	-	-

Supplementary Table 9: Age groups of children newly experiencing orphanhood in the US from 2000 to 2021.

LaTeX template

	2000 (Number of Children)	2019 (Number of Children)	2000-2019 changes	2020 (Number of Children)	2021 (Number of Children)	2019-2021 changes	2000-2021 changes
	**Before the COVID-19 pandemic**			**Since the COVID-19 pandemic**			
**Incidence (n, (95% uncertainty interval))**							
**Total***	262,036 (237,122, 290,407)	292,118 (265,553, 321,908)	+11.5% (+10.9%, +12.0%)	360,192 (329,378, 394,370)	407,377 (374,905, 442,874)	+39.4% (+37.6%, +41.2%)	+55.5% (+52.5%, +58.1%)
**Mother†**	90,377 (79,380, 103,234)	100,066 (88,525, 113,265)	+10.7% (+9.7%, +11.5%)	119,969 (106,773, 134,938)	137,202 (123,350, 152,612)	+37.1% (+34.8%, +39.3%)	+51.8% (+47.8%, +55.4%)
**Father‡**	171,662 (157,744, 187,177)	192,056 (177,031, 208,647)	+11.9% (+11.5%, +12.2%)	240,231 (222,611, 259,441)	270,188 (251,565, 290,278)	+40.7% (+39.1%, +42.1%)	+57.4% (+55.1%, +59.5%)
**Incidence rate per 100 children (rate, (95% uncertainty interval))**							
**Total**	0.36 (0.33, 0.40)	0.40 (0.36, 0.44)	+10.4% (+9.8%, +10.9%)	0.49 (0.45, 0.54)	0.59 (0.54, 0.64)	+46.9% (+44.9%, +48.7%)	+62.1% (+59.1%, +64.9%)
**Mother**	0.12 (0.11, 0.14)	0.14 (0.12, 0.15)	+9.6% (+8.6%, +10.4%)	0.16 (0.15, 0.19)	0.20 (0.18, 0.22)	+44.4% (+41.9%, +46.8%)	+58.3% (+54.2%, +62.1%)
**Father**	0.24 (0.22, 0.26)	0.26 (0.24, 0.29)	+10.8% (+10.4%, +11.2%)	0.33 (0.31, 0.36)	0.39 (0.36, 0.42)	+48.2% (+46.5%, +49.7%)	+64.2% (+61.7%, +66.3%)
**Incidence rate ratio (ratio relative to maternal orphanhood; 95% UI)**							
**Mother**	1.00 (1.00, 1.00)	1.00 (1.00, 1.00)	-	1.00 (1.00, 1.00)	1.00 (1.00, 1.00)	-	-
**Father**	1.90 (1.81, 1.99)	1.92 (1.84, 2.00)	-	2.00 (1.92, 2.08)	1.97 (1.90, 2.04)	-	-
**Prevalence (n, (95 uncertainty interval))**							
**Total**	2,220,606 (2,001,053, 2,472,064)	2,159,537 (1,941,498, 2,408,054)	-2.7% (-3.0%, -2.6%)	2,251,322 (2,027,683, 2,505,227)	2,378,250 (2,148,223, 2,638,221)	+10.1% (+9.6%, +10.6%)	+7.1% (+6.7%, +7.4%)
**Mother**	700,049 (606,552, 810,479)	720,770 (627,664, 829,774)	+3.0% (+2.4%, +3.5%)	748,383 (653,059, 859,658)	789,423 (691,479, 903,061)	+9.5% (+8.8%, +10.2%)	+12.8% (+11.4%, +14.0%)
**Father**	1,520,560 (1,394,504, 1,661,590)	1,438,772 (1,313,838, 1,578,285)	-5.4% (-5.8%, -5.0%)	1,502,948 (1,374,630, 1,645,578)	1,588,843 (1,456,757, 1,735,178)	+10.4% (+9.9%, +10.9%)	+4.5% (+4.4%, +4.5%)
**Prevalence rate per 100 children (rate, (95% uncertainty interval))**							
**Total**	3.07 (2.76, 3.42)	2.95 (2.66, 3.29)	-3.7% (-3.9%, -3.5%)	3.09 (2.78, 3.44)	3.43 (3.10, 3.80)	+16.0% (+15.4%, +16.5%)	+11.7% (+11.3%, +12.0%)
**Mother**	0.97 (0.84, 1.12)	0.99 (0.86, 1.14)	+2.0% (+1.4%, +2.5%)	1.03 (0.90, 1.18)	1.14 (1.00, 1.30)	+15.4% (+14.6%, +16.0%)	+17.6% (+16.2%, +18.9%)
**Father**	2.10 (1.93, 2.30)	1.97 (1.80, 2.16)	-6.3% (-6.7%, -5.9%)	2.06 (1.89, 2.26)	2.29 (2.10, 2.50)	+16.3% (+15.8%, +16.8%)	+9.0% (+8.9%, +9.0%)
**Prevalence rate ratio (ratio relative to maternal orphanhood; 95% UI)**							
**Mother**	1.00 (1.00, 1.00)	1.00 (1.00, 1.00)	-	1.00 (1.00, 1.00)	1.00 (1.00, 1.00)	-	-
**Father**	2.17 (2.05, 2.30)	2.00 (1.90, 2.09)	-	2.01 (1.91, 2.10)	2.01 (1.92, 2.11)	-	-

*: Children experiencing the death of one or both parents; Eq. (7)

†: Children experiencing the death of their mother, including children experiencing the death of both their mother and father; Eq. (9)

‡: Children experiencing the death of their father, including children experiencing the death of both their father and mother. Eq. (9)

Supplementary Table 10: Sex of parent of children newly experiencing orphanhood in the US from 2000 to 2021.

	Before the COVID-19 pandemic			Since the COVID-19 pandemic			
	**2000 (Number of Children)**	**2019 (Number of Children)**	**2000-2019 changes**	**2020 (Number of Children)**	**2021 (Number of Children)**	**2019-2021 changes**	**2000-2021 changes**
**Incidence (n, (95% uncertainty interval))**							
**Total**	262,036 (237,122, 290,407)	292,118 (265,553, 321,908)	+11.5% (+10.9%, +12.0%)	360,192 (329,378, 394,370)	407,377 (374,905, 442,874)	+39.4% (+37.6%, +41.2%)	+55.5% (+52.5%, +58.1%)
**Non-Hispanic American Indian or Alaska Native**	2,497 (1,433, 3,959)	4,213 (2,821, 6,056)	+69.7% (+53.3%, +96.8%)	6,190 (4,380, 8,492)	6,907 (4,958, 9,158)	+64.0% (+50.9%, +75.9%)	+178.0% (+131.4%, +245.9%)
**Non-Hispanic Asian**	5,524 (3,946, 7,657)	9,120 (7,017, 11,710)	+65.1% (+52.9%, +77.8%)	11,510 (9,074, 14,583)	11,852 (9,405, 14,818)	+30.0% (+26.6%, +33.8%)	+114.4% (+93.5%, +138.3%)
**Non-Hispanic Black**	58,468 (51,597, 66,116)	57,664 (51,200, 64,756)	-1.4% (-2.0%, -0.8%)	75,604 (68,010, 83,934)	84,172 (76,204, 92,857)	+46.0% (+43.4%, +48.9%)	+44.0% (+40.4%, +47.7%)
**Hispanic**	30,971 (25,843, 37,141)	46,644 (40,628, 53,750)	+50.6% (+44.7%, +57.1%)	66,979 (59,579, 75,184)	78,122 (70,284, 86,788)	+67.5% (+61.5%, +73.0%)	+152.2% (+133.7%, +172.0%)
**non-Hispanic white**	164,576 (154,302, 175,535)	174,477 (163,887, 185,636)	+6.0% (+5.8%, +6.2%)	199,909 (188,334, 212,177)	226,324 (214,053, 239,253)	+29.7% (+28.9%, +30.6%)	+37.5% (+36.3%, +38.7%)
**Incidence rate per 100 children (rate, (95% uncertainty interval))**							
**Total**	0.36 (0.33, 0.40)	0.40 (0.36, 0.44)	+10.4% (+9.8%, +10.9%)	0.49 (0.45, 0.54)	0.59 (0.54, 0.64)	+46.9% (+44.9%, +48.7%)	+62.1% (+59.1%, +64.9%)
**Non-Hispanic American Indian or Alaska Native**	0.32 (0.18, 0.51)	0.59 (0.40, 0.85)	+86.8% (+68.8%, +116.7%)	0.88 (0.62, 1.21)	1.20 (0.86, 1.59)	+102.5% (+86.4%, +117.1%)	+277.9% (+214.5%, +370.2%)
**Non-Hispanic Asian**	0.19 (0.14, 0.26)	0.20 (0.15, 0.26)	+4.9% (-2.8%, +13.0%)	0.25 (0.20, 0.32)	0.29 (0.23, 0.36)	+42.8% (+39.1%, +47.0%)	+49.7% (+35.1%, +66.4%)
**Non-Hispanic Black**	0.52 (0.46, 0.59)	0.52 (0.46, 0.58)	-0.7% (-1.3%, -0.1%)	0.68 (0.61, 0.75)	0.84 (0.76, 0.92)	+61.3% (+58.5%, +64.6%)	+60.3% (+56.3%, +64.4%)
**Hispanic**	0.25 (0.21, 0.30)	0.25 (0.22, 0.29)	+0.9% (-3.1%, +5.2%)	0.36 (0.32, 0.40)	0.42 (0.37, 0.46)	+66.1% (+60.1%, +71.6%)	+67.5% (+55.2%, +80.6%)
**non-Hispanic white**	0.37 (0.34, 0.39)	0.46 (0.43, 0.49)	+25.3% (+25.0%, +25.6%)	0.53 (0.50, 0.56)	0.63 (0.60, 0.67)	+37.8% (+37.0%, +38.8%)	+72.7% (+71.2%, +74.2%)
**Incidence rate ratio (ratio relative to Non-Hispanic Asian children; 95% UI)**							
**Non-Hispanic American Indian or Alaska Native**	1.66 (1.34, 1.91)	2.96 (2.57, 3.31)	-	3.50 (3.14, 3.79)	4.19 (3.80, 4.45)	-	-
**Non-Hispanic Asian**	1.00 (1.00, 1.00)	1.00 (1.00, 1.00)	-	1.00 (1.00, 1.00)	1.00 (1.00, 1.00)	-	-
**Non-Hispanic Black**	2.73 (2.23, 3.37)	2.58 (2.26, 2.98)	-	2.71 (2.37, 3.09)	2.92 (2.58, 3.33)	-	-
**Hispanic**	1.30 (1.13, 1.52)	1.25 (1.12, 1.42)	-	1.43 (1.27, 1.62)	1.46 (1.29, 1.65)	-	-
**non-Hispanic white**	1.91 (1.47, 2.51)	2.28 (1.89, 2.79)	-	2.11 (1.77, 2.52)	2.21 (1.86, 2.63)	-	-
**Prevalence (n, (95% uncertainty interval))**							
**Total**	2,220,606 (2,001,053, 2,472,064)	2,159,537 (1,941,498, 2,408,054)	-2.7% (-3.0%, -2.6%)	2,251,322 (2,027,683, 2,505,227)	2,378,250 (2,148,223, 2,638,221)	+10.1% (+9.6%, +10.6%)	+7.1% (+6.7%, +7.4%)
**Non-Hispanic American Indian or Alaska Native**	20,561 (11,700, 32,659)	30,515 (19,453, 45,781)	+48.8% (+40.2%, +66.3%)	33,389 (21,733, 49,258)	36,749 (24,395, 53,121)	+20.5% (+16.0%, +25.4%)	+79.1% (+62.7%, +108.5%)
**Non-Hispanic Asian**	39,597 (26,965, 57,050)	60,711 (44,469, 81,590)	+53.3% (+43.0%, +64.9%)	64,836 (48,011, 86,406)	68,741 (51,420, 90,823)	+13.2% (+11.3%, +15.6%)	+73.6% (+59.2%, +90.7%)
**Non-Hispanic Black**	533,644 (470,653, 604,344)	418,707 (366,251, 477,991)	-21.5% (-22.2%, -20.9%)	443,065 (389,101, 503,818)	474,139 (418,373, 536,615)	+13.2% (+12.3%, +14.2%)	-11.1% (-11.3%, -11.1%)
**Hispanic**	269,114 (224,165, 322,767)	336,445 (286,308, 394,470)	+25.0% (+22.2%, +27.7%)	364,278 (312,408, 423,803)	399,746 (346,059, 461,079)	+18.8% (+16.9%, +20.9%)	+48.5% (+42.9%, +54.4%)
**non-Hispanic white**	1,357,690 (1,267,569, 1,455,244)	1,313,158 (1,225,016, 1,408,221)	-3.3% (-3.4%, -3.2%)	1,345,752 (1,256,429, 1,441,941)	1,398,875 (1,307,976, 1,496,582)	+6.5% (+6.3%, +6.8%)	+3.0% (+2.8%, +3.2%)
**Prevalence rate per 100 children (rate, (95% uncertainty interval))**							
**Total**	3.07 (2.76, 3.42)	2.95 (2.66, 3.29)	-3.7% (-3.9%, -3.5%)	3.09 (2.78, 3.44)	3.27 (2.95, 3.62)	+10.5% (+10.0%, +11.1%)	+6.5% (+6.1%, +6.7%)
**Non-Hispanic American Indian or Alaska Native**	2.63 (1.50, 4.17)	4.29 (2.74, 6.44)	+63.8% (+54.3%, +83.1%)	4.74 (3.09, 6.99)	6.38 (4.24, 9.23)	+48.7% (+43.3%, +54.8%)	+143.4% (+121.1%, +183.4%)
**Non-Hispanic Asian**	1.37 (0.93, 1.97)	1.33 (0.98, 1.79)	-2.6% (-9.1%, +4.8%)	1.41 (1.05, 1.88)	1.66 (1.24, 2.19)	+24.4% (+22.3%, +27.0%)	+21.2% (+11.1%, +33.1%)
**Non-Hispanic Black**	4.76 (4.20, 5.39)	3.76 (3.29, 4.29)	-21.0% (-21.6%, -20.4%)	3.98 (3.50, 4.53)	4.71 (4.15, 5.33)	+25.2% (+24.1%, +26.3%)	-1.1% (-1.2%, -1.0%)
**Hispanic**	2.16 (1.80, 2.59)	1.81 (1.54, 2.12)	-16.3% (-18.2%, -14.5%)	1.96 (1.68, 2.27)	2.13 (1.85, 2.46)	+17.8% (+15.9%, +19.9%)	-1.4% (-5.1%, +2.5%)
**non-Hispanic white**	3.01 (2.81, 3.23)	3.45 (3.21, 3.70)	+14.3% (+14.2%, +14.4%)	3.56 (3.33, 3.82)	3.90 (3.65, 4.17)	+13.2% (+12.9%, +13.5%)	+29.4% (+29.2%, +29.6%)
**Prevalence rate ratio (ratio relative to Non-Hispanic Asian children; 95% UI)**							
**Non-Hispanic American Indian or Alaska Native**	1.92 (1.60, 2.11)	3.22 (2.80, 3.59)	-	3.35 (2.95, 3.71)	3.85 (3.41, 4.21)	-	-
**Non-Hispanic Asian**	1.00 (1.00, 1.00)	1.00 (1.00, 1.00)	-	1.00 (1.00, 1.00)	1.00 (1.00, 1.00)	-	-
**Non-Hispanic Black**	3.48 (2.73, 4.50)	2.82 (2.39, 3.37)	-	2.82 (2.40, 3.34)	2.84 (2.43, 3.35)	-	-
**Hispanic**	1.58 (1.31, 1.93)	1.36 (1.18, 1.58)	-	1.38 (1.21, 1.60)	1.28 (1.12, 1.49)	-	-
**non-Hispanic white**	2.20 (1.64, 3.02)	2.58 (2.06, 3.29)	-	2.52 (2.02, 3.17)	2.35 (1.90, 2.94)	-	-

Supplementary Table 11: Racial and ethnicity of children newly experiencing orphanhood in the US from 2000 to 2021.

LaTeX template

Race and ethnicity	Parental cause of death	Orphanhood* Incidence rate per 100,000 children			Maternal Orphanhood† Incidence rate per 100,000 children			Paternal Orphanhood‡ Incidence rate per 100,000 children		
		2000 rate, (95% UI)	2021 rate, (95% UI)	2000-2021 changes	2000 rate, (95% UI)	2021 rate, (95% UI)	2000-2021 changes	2000 rate, (95% UI)	2021 rate, (95% UI)	2000-2021 changes
Non-Hispanic American Indian or Alaska Native	COVID-19	0.00	173.70	-	0.00	73.65	-	0.00	100.05	-
		(0.00, 0.00)	(134.62, 212.83)		(0.00, 0.00)	(55.58, 93.66)		(0.00, 0.00)	(79.03, 119.18)	
	Drug overdose	12.78	167.62	+1212.99%	6.39	74.87	+1074.33%	6.39	92.76	+1356.71%
		(4.34, 24.93)	(129.75, 208.83)	(+737.80%, +2890.06%)	(2.04, 13.17)	(55.75, 94.52)	(+617.92%, +2624.95%)	(2.30, 11.76)	(74.00, 114.32)	(+865.99%, +3118.50%)
	Unintentional injuries excluding drug overdose	87.15	140.52	+63.35%	35.27	54.02	+54.67%	51.88	86.50	+69.15%
		(61.45, 112.34)	(106.65, 177.00)	(+57.57%, +72.14%)	(23.12, 47.54)	(38.39, 71.39)	(+46.90%, +62.20%)	(38.33, 64.80)	(68.26, 105.61)	(+62.89%, +76.96%)
	Suicide excluding drug overdose	22.24	65.66	+197.57%	6.13	18.41	+203.98%	16.10	47.25	+196.71%
		(9.20, 34.92)	(44.98, 92.43)	(+160.49%, +398.48%)	(0.00, 11.40)	(10.07, 29.72)	(+159.09%, +1411.69%)	(9.20, 23.51)	(34.90, 62.71)	(+161.05%, +285.22%)
	Homicide excluding drug overdose	15.72	36.13	+128.75%	5.62	8.34	+51.40%	10.10	27.79	+171.88%
		(6.01, 27.09)	(18.35, 53.16)	(+95.41%, +197.71%)	(0.00, 9.84)	(1.33, 15.11)	(+36.03%, +171.35%)	(6.01, 17.25)	(17.02, 38.05)	(+117.88%, +200.69%)
	Heart disease	38.08	83.72	+121.82%	11.12	26.23	+140.56%	26.96	57.50	+112.61%
		(22.99, 56.23)	(58.19, 111.37)	(+98.13%, +149.73%)	(4.86, 19.81)	(15.63, 38.56)	(+94.80%, +191.18%)	(18.14, 36.42)	(42.56, 72.81)	(+98.99%, +136.29%)
	Malignant neoplasms	35.40	55.06	+58.40%	17.64	27.97	+60.49%	17.76	27.10	+54.04%
		(20.79, 51.76)	(33.87, 78.35)	(+49.48%, +70.39%)	(9.80, 26.45)	(17.89, 39.79)	(+49.81%, +81.90%)	(10.99, 25.30)	(15.98, 38.56)	(+46.24%, +64.38%)
	Chronic liver disease and cirrhosis**	35.91	187.95	+424.86%	17.89	89.98	+410.73%	18.02	97.97	+443.67%
		(22.11, 51.76)	(149.68, 230.90)	(+347.92%, +574.20%)	(9.58, 25.05)	(69.80, 111.55)	(+347.19%, +618.06%)	(12.52, 26.71)	(79.87, 119.36)	(+347.46%, +545.14%)
	Cerebrovascular diseases	3.71	12.16	+185.16%	1.92	6.60	+129.34%	1.79	5.56	+204.15%
		(0.13, 13.56)	(1.04, 22.94)	(+75.82%, +445.10%)	(0.13, 8.32)	(0.52, 12.16)	(+54.66%, +283.33%)	(0.00, 5.25)	(0.52, 10.78)	(+105.53%, +838.46%)
	Chronic lower respiratory diseases	0.26	3.13	+171.35%	0.00	1.91	+172.17%	0.26	1.22	+172.55%
		(0.00, 5.76)	(0.00, 10.07)	(+77.09%, +2307.69%)	(0.00, 2.68)	(0.00, 6.95)	(+106.27%, +251.69%)	(0.00, 3.08)	(0.00, 3.13)	(+23.72%, +838.46%)
Non-Hispanic Asian	COVID-19	0.00	52.28	-	0.00	15.55	-	0.00	36.73	-
		(0.00, 0.00)	(45.03, 59.83)		(0.00, 0.00)	(12.60, 18.57)		(0.00, 0.00)	(32.43, 41.27)	
	Drug overdose	3.22	16.90	+425.78%	1.14	4.01	+245.45%	2.08	12.89	+523.56%
		(1.11, 6.23)	(13.00, 21.30)	(+242.22%, +1061.80%)	(0.03, 2.56)	(2.61, 5.60)	(+118.26%, +9233.33%)	(1.07, 3.67)	(10.41, 15.70)	(+330.74%, +870.23%)
	Unintentional injuries excluding drug overdose	19.93	15.98	-19.17%	7.58	4.44	-40.06%	12.35	11.54	-6.56%
		(14.14, 26.08)	(12.24, 20.40)	(-22.04%, -13.04%)	(4.98, 10.31)	(2.97, 6.25)	(-41.64%, -37.25%)	(9.17, 15.77)	(9.27, 14.15)	(-10.22%, +1.14%)
	Suicide excluding drug overdose	10.48	14.27	+36.16%	2.70	3.86	+44.32%	7.78	10.41	+33.79%
		(6.53, 14.60)	(10.58, 18.11)	(+24.44%, +61.89%)	(1.07, 4.32)	(2.51, 5.38)	(+22.85%, +134.58%)	(5.46, 10.27)	(8.06, 12.73)	(+24.82%, +50.09%)
	Homicide excluding drug overdose	7.02	3.84	-45.24%	2.53	1.23	-53.33%	4.50	2.61	-40.48%
		(3.42, 10.79)	(1.62, 6.08)	(-55.17%, -43.11%)	(0.66, 4.19)	(0.14, 2.10)	(-85.57%, -49.54%)	(2.77, 6.61)	(1.47, 3.98)	(-46.96%, -37.86%)
	Heart disease	33.87	43.34	+27.91%	5.57	6.79	+19.14%	28.30	36.56	+29.15%
		(27.15, 41.69)	(37.40, 49.81)	(+19.23%, +38.00%)	(3.81, 8.27)	(5.02, 8.79)	(+5.90%, +30.00%)	(23.35, 33.42)	(32.38, 41.02)	(+22.48%, +39.12%)
	Malignant neoplasms**	70.26	68.94	-2.02%	32.86	33.32	+1.30%	37.40	35.62	-4.95%
		(59.42, 82.24)	(60.38, 77.50)	(-5.66%, +1.84%)	(28.01, 38.51)	(29.14, 37.72)	(-2.10%, +4.65%)	(31.41, 43.73)	(31.25, 39.77)	(-8.66%, -0.36%)

See footnotes at the end of table.

Supplementary Table 12: Leading causes of orphanhood among US children in 2021 by race and ethnicity and sex of parent.

Race and ethnicity	Parental cause of death	Orphanhood * Incidence rate per 100,000 children			Maternal Orphanhood† Incidence rate per 100,000 children			Paternal Orphanhood‡ Incidence rate per 100,000 children		
		**2000 rate, (95% UI)**	**2021 rate, (95% UI)**	**2000-2021 changes**	**2000 rate, (95% UI)**	**2021 rate, (95% UI)**	**2000-2021 changes**	**2000 rate, (95% UI)**	**2021 rate, (95% UI)**	**2000-2021 changes**
Non-Hispanic Asian	Chronic liver disease	3.39	7.85	+133.99%	0.55	1.88	+254.84%	2.84	5.96	+111.62%
	and cirrhosis	(1.25, 5.50)	(5.22, 10.87)	(+100.36%, +315.32%)	(0.00, 1.07)	(0.99, 2.99)	(+191.86%, +1250.00%)	(1.25, 4.43)	(4.23, 7.87)	(+79.62%, +236.12%)
	Cerebrovascular diseases	11.45	12.39	+8.21%	4.91	4.18	-15.41%	6.54	8.21	+25.17%
		(7.89, 16.19)	(8.81, 15.82)	(-2.80%, +16.34%)	(3.36, 7.13)	(2.73, 5.67)	(-20.68%, -8.81%)	(4.53, 9.06)	(6.08, 10.14)	(+10.82%, +36.84%)
	Chronic lower respiratory diseases	2.14	0.99	-53.62%	0.66	0.10	-83.56%	1.49	0.89	-45.36%
		(0.59, 4.81)	(0.10, 2.34)	(-84.12%, -49.28%)	(0.00, 1.83)	(0.00, 0.94)	(-95.56%, -47.06%)	(0.59, 2.98)	(0.10, 1.40)	(-83.05%, -24.44%)
Non-Hispanic Black	COVID-19	0.00	105.84	-	0.00	44.97	-	0.00	60.88	-
		(0.00, 0.00)	(98.72, 113.34)		(0.00, 0.00)	(41.67, 48.46)		(0.00, 0.00)	(57.05, 64.88)	
	Drug overdose	18.15	115.41	+536.07%	6.99	37.22	+433.62%	11.15	78.19	+600.00%
		(15.43, 21.12)	(107.73, 123.16)	(+483.11%, +598.19%)	(5.67, 8.35)	(34.03, 40.46)	(+384.32%, +500.36%)	(9.76, 12.77)	(73.70, 82.71)	(+547.66%, +655.14%)
	Unintentional injuries	42.34	63.75	+50.71%	13.78	18.13	+31.93%	28.56	45.61	+59.70%
	excluding drug overdose	(37.80, 46.81)	(58.36, 69.25)	(+47.94%, +54.17%)	(11.69, 15.71)	(16.10, 20.34)	(+29.46%, +36.52%)	(26.11, 31.10)	(42.27, 48.91)	(+57.32%, +61.89%)
	Suicide	10.18	19.59	+92.44%	2.07	3.93	+90.48%	8.11	15.66	+93.09%
	excluding drug overdose	(8.19, 12.21)	(16.74, 22.53)	(+83.86%, +104.45%)	(1.35, 2.85)	(3.01, 4.98)	(+74.62%, +125.00%)	(6.84, 9.37)	(13.73, 17.56)	(+86.51%, +100.74%)
	Homicide	44.69	81.29	+81.96%	11.58	14.46	+24.62%	33.11	66.83	+102.03%
	excluding drug overdose	(40.38, 49.22)	(75.51, 87.18)	(+77.30%, +87.21%)	(9.85, 13.54)	(12.53, 16.39)	(+21.36%, +27.42%)	(30.53, 35.68)	(62.97, 70.80)	(+98.58%, +106.29%)
	Heart disease**	89.47	128.29	+43.38%	32.57	37.73	+15.81%	56.90	90.56	+59.16%
		(83.18, 96.11)	(120.36, 136.21)	(+41.76%, +44.77%)	(29.67, 35.61)	(34.57, 40.85)	(+14.88%, +16.64%)	(53.51, 60.50)	(85.79, 95.37)	(+57.65%, +60.40%)
	Malignant neoplasms	86.37	74.80	-13.41%	42.09	34.32	-18.42%	44.28	40.48	-8.69%
		(79.99, 92.64)	(69.11, 80.63)	(-13.79%, -12.97%)	(38.75, 45.37)	(31.49, 37.29)	(-18.76%, -17.81%)	(41.24, 47.26)	(37.62, 43.34)	(-9.18%, -8.28%)
	Chronic liver disease	9.80	13.70	+39.38%	3.53	6.02	+69.50%	6.26	7.69	+22.49%
	and cirrhosis	(8.08, 11.89)	(11.34, 16.23)	(+36.11%, +40.77%)	(2.74, 4.57)	(4.86, 7.22)	(+57.93%, +78.07%)	(5.34, 7.32)	(6.48, 9.01)	(+20.58%, +23.85%)
	Cerebrovascular diseases	21.05	21.45	+2.00%	9.97	7.69	-22.81%	11.09	13.76	+24.41%
		(18.26, 23.94)	(18.56, 24.54)	(+1.42%, +2.68%)	(8.49, 11.54)	(6.42, 9.01)	(-24.33%, -21.80%)	(9.77, 12.39)	(12.15, 15.53)	(+23.48%, +25.36%)
	Chronic lower respiratory diseases	7.95	8.73	+10.25%	3.64	3.19	-10.97%	4.31	5.54	+27.70%
		(6.03, 10.04)	(6.95, 10.83)	(+7.80%, +14.78%)	(2.69, 4.68)	(2.37, 4.17)	(-12.64%, -9.46%)	(3.34, 5.35)	(4.58, 6.66)	(+24.45%, +36.26%)
Hispanic	COVID-19**	0.00	98.19	-	0.00	28.10	-	0.00	70.08	-
		(0.00, 0.00)	(92.67, 103.79)		(0.00, 0.00)	(25.95, 30.30)		(0.00, 0.00)	(66.72, 73.48)	
	Drug overdose	13.66	57.61	+321.74%	3.13	14.62	+367.09%	10.53	42.98	+308.26%
		(11.18, 16.39)	(53.57, 61.93)	(+278.18%, +379.19%)	(2.20, 4.15)	(13.07, 16.27)	(+291.07%, +495.91%)	(8.98, 12.23)	(40.50, 45.66)	(+273.58%, +351.12%)
	Unintentional injuries	40.07	40.36	+0.72%	9.62	9.67	+0.63%	30.45	30.69	+0.72%
	excluding drug overdose	(35.68, 44.68)	(36.95, 43.87)	(-1.84%, +3.47%)	(7.89, 11.34)	(8.40, 10.98)	(-3.53%, +6.46%)	(27.80, 33.34)	(28.55, 32.89)	(-1.32%, +2.68%)

See footnotes at the end of table.

Supplementary Table 12: Leading causes of orphanhood among US children in 2021 by race and ethnicity and sex of parent.

Race and ethnicity	Parental cause of death	Orphanhood* Incidence rate per 100,000 children			Maternal Orphanhood† Incidence rate per 100,000 children			Paternal Orphanhood‡ Incidence rate per 100,000 children		
		2000 rate, (95% UI)	2021 rate, (95% UI)	2000-2021 changes	2000 rate, (95% UI)	2021 rate, (95% UI)	2000-2021 changes	2000 rate, (95% UI)	2021 rate, (95% UI)	2000-2021 changes
Hispanic	Suicide	10.86	16.85	+55.12%	1.72	3.42	+99.42%	9.14	13.43	+46.86%
	excluding drug overdose	(8.71, 13.23)	(14.73, 19.05)	(+43.98%, +68.89%)	(1.03, 2.51)	(2.69, 4.24)	(+69.05%, +162.14%)	(7.68, 10.72)	(12.04, 14.80)	(+38.07%, +56.40%)
	Homicide	18.36	16.49	-10.21%	4.02	3.52	-12.50%	14.34	12.97	-9.56%
	excluding drug overdose	(15.48, 21.51)	(14.47, 18.69)	(-13.01%, -6.41%)	(2.98, 5.25)	(2.75, 4.30)	(-18.11%, -7.36%)	(12.50, 16.25)	(11.72, 14.39)	(-11.43%, -6.35%)
	Heart disease	27.43	34.82	+26.88%	6.64	7.71	+16.14%	20.79	27.11	+30.35%
		(23.96, 31.03)	(31.83, 37.80)	(+21.79%, +32.90%)	(5.27, 8.06)	(6.64, 8.79)	(+9.06%, +25.62%)	(18.69, 22.98)	(25.20, 29.01)	(+26.19%, +35.11%)
	Malignant neoplasms	42.07	43.35	+3.05%	21.60	22.27	+3.24%	20.47	21.08	+2.98%
		(37.40, 46.91)	(39.81, 47.02)	(+0.26%, +6.46%)	(19.12, 24.17)	(20.41, 24.22)	(+0.12%, +6.73%)	(18.28, 22.73)	(19.40, 22.81)	(+0.39%, +6.28%)
	Chronic liver disease	13.02	20.96	+61.06%	2.26	4.99	+122.07%	10.76	15.97	+48.42%
	and cirrhosis	(10.89, 15.47)	(18.67, 23.45)	(+51.58%, +71.06%)	(1.59, 3.08)	(4.10, 5.92)	(+90.85%, +156.59%)	(9.30, 12.39)	(14.57, 17.53)	(+41.56%, +56.47%)
	Cerebrovascular diseases	8.21	9.05	+9.99%	3.42	2.81	-18.65%	4.79	6.24	+30.27%
		(6.27, 10.44)	(7.52, 10.66)	(+2.01%, +19.49%)	(2.54, 4.43)	(2.19, 3.46)	(-23.44%, -13.20%)	(3.74, 6.02)	(5.33, 7.20)	(+19.60%, +40.99%)
	Chronic lower respiratory diseases	1.69	1.75	+3.55%	0.79	0.75	-5.00%	0.90	1.00	+11.11%
		(0.85, 2.76)	(1.06, 2.49)	(-8.83%, +33.33%)	(0.39, 1.34)	(0.42, 1.13)	(-15.15%, +23.40%)	(0.47, 1.42)	(0.64, 1.36)	(-3.57%, +48.94%)
non-Hispanic white	COVID-19	0.00	68.95	-	0.00	24.02	-	0.00	44.94	-
		(0.00, 0.00)	(66.15, 71.81)		(0.00, 0.00)	(22.83, 25.24)		(0.00, 0.00)	(43.32, 46.58)	
	Drug overdose**	23.24	130.99	+463.64%	8.15	45.37	+456.07%	15.08	85.62	+467.64%
		(21.77, 24.77)	(126.80, 135.25)	(+446.01%, +482.32%)	(7.51, 8.83)	(43.56, 47.23)	(+435.11%, +480.17%)	(14.26, 15.94)	(83.24, 88.01)	(+452.00%, +483.66%)
	Unintentional injuries	46.33	50.89	+9.83%	13.02	14.79	+13.56%	33.31	36.11	+8.37%
	excluding drug overdose	(44.15, 48.55)	(48.39, 53.42)	(+9.59%, +10.15%)	(12.15, 13.89)	(13.81, 15.79)	(+13.27%, +13.93%)	(32.00, 34.66)	(34.58, 37.63)	(+8.14%, +8.65%)
	Suicide	25.35	40.11	+58.28%	4.58	7.98	+74.07%	20.77	32.13	+54.77%
	excluding drug overdose	(23.82, 26.96)	(38.01, 42.32)	(+56.98%, +59.43%)	(4.09, 5.11)	(7.28, 8.74)	(+71.26%, +77.76%)	(19.73, 21.86)	(30.73, 33.58)	(+53.63%, +55.66%)
	Homicide	7.20	9.10	+26.33%	2.72	2.92	+7.12%	4.48	6.18	+37.94%
	excluding drug overdose	(6.36, 8.07)	(8.07, 10.18)	(+25.74%, +27.03%)	(2.34, 3.12)	(2.49, 3.38)	(+5.88%, +8.70%)	(4.01, 4.95)	(5.58, 6.80)	(+37.04%, +39.31%)
	Heart disease	63.78	77.26	+21.09%	13.51	20.14	+49.01%	50.28	57.14	+13.62%
		(61.47, 66.16)	(74.38, 80.25)	(+20.93%, +21.30%)	(12.73, 14.32)	(19.00, 21.24)	(+48.25%, +49.46%)	(48.74, 51.84)	(55.39, 59.02)	(+13.50%, +13.87%)
	Malignant neoplasms	83.54	76.67	-8.23%	37.36	34.80	-6.88%	46.19	41.88	-9.32%
		(80.71, 86.44)	(73.74, 79.63)	(-8.64%, -7.87%)	(36.00, 38.77)	(33.39, 36.24)	(-7.29%, -6.51%)	(44.71, 47.68)	(40.36, 43.40)	(-9.73%, -8.92%)
	Chronic liver disease	12.78	29.59	+131.50%	3.40	10.69	+215.29%	9.38	18.90	+101.17%
	and cirrhosis	(11.81, 13.79)	(27.73, 31.40)	(+127.86%, +135.13%)	(3.02, 3.80)	(9.88, 11.49)	(+202.62%, +227.21%)	(8.79, 9.99)	(17.86, 19.92)	(+99.32%, +103.48%)
	Cerebrovascular diseases	9.18	10.64	+16.06%	3.79	4.07	+6.95%	5.38	6.57	+22.50%
		(8.27, 10.09)	(9.64, 11.71)	(+15.65%, +16.55%)	(3.38, 4.24)	(3.59, 4.56)	(+6.18%, +7.56%)	(4.89, 5.86)	(6.05, 7.15)	(+21.92%, +23.56%)
	Chronic lower respiratory diseases	5.70	8.09	+42.09%	2.14	3.22	+50.48%	3.56	4.87	+37.05%
		(5.05, 6.35)	(7.26, 8.90)	(+40.31%, +43.48%)	(1.82, 2.46)	(2.82, 3.59)	(+46.75%, +54.14%)	(3.23, 3.90)	(4.43, 5.31)	(+35.99%, +37.86%)

*: Orphanhood – "Children experiencing the death of one or both parents; Eq. (7)"
†: Maternal orphanhood – "Children experiencing the death of their mother, including children experiencing the death of both their mother and father; Eq. (9)"
‡: Paternal orphanhood – "Children experiencing the death of their father, including children experiencing the death of both their father and mother; Eq. (9)"
**: Leading parental cause of death.

Supplementary Table 12: Leading causes of orphanhood among US children in 2021 by race and ethnicity and sex of parent (continued).

Race and ethnicity	Parental cause of death	Orphanhood* Incidence rate per 100,000 children			Maternal Orphanhood† Incidence rate per 100,000 children			Paternal Orphanhood‡ Incidence rate per 100,000 children		
		2000 rate, (95% UI)	2019 rate, (95% UI)	2000-2019 changes	2000 rate, (95% UI)	2019 rate, (95% UI)	2000-2019 changes	2000 rate, (95% UI)	2019 rate, (95% UI)	2000-2019 changes
Non-Hispanic American Indian	Drug overdose	12.78	83.15	+550.7%	6.39	38.55	+503.3%	6.39	44.60	+598.0%
or Alaska Native		(4.34, 24.93)	(59.23, 108.50)	(+335.3%, +1263.3%)	(2.04, 13.17)	(26.17, 50.95)	(+287.8%, +1179.9%)	(2.30, 11.76)	(33.06, 57.55)	(+387.1%, +1337.4%)
	Unintentional injuries	87.15	97.08	+14.1%	35.27	36.86	+6.4%	51.88	60.22	+19.2%
	excluding drug overdose	(61.45, 112.33)	(72.01, 127.35)	(+12.0%, +17.1%)	(23.12, 47.54)	(25.30, 51.21)	(+4.4%, +9.2%)	(38.33, 64.80)	(46.70, 76.14)	(+16.6%, +22.2%)
	Suicide	22.24	47.41	+113.0%	6.13	14.07	+129.4%	16.10	33.34	+108.0%
	excluding drug overdose	(9.20, 34.91)	(29.96, 66.41)	(+89.6%, +228.8%)	(0.00, 11.40)	(6.61, 22.09)	(+92.3%, +982.6%)	(9.20, 23.51)	(23.36, 44.33)	(+86.7%, +163.7%)
	Homicide	15.72	27.15	+72.8%	5.62	8.72	+54.0%	10.10	18.43	+82.2%
	excluding drug overdose	(6.01, 27.09)	(12.52, 41.08)	(+50.9%, +110.7%)	(0.00, 9.84)	(2.25, 15.05)	(+35.1%, +246.0%)	(6.01, 17.25)	(10.27, 26.03)	(+50.8%, +104.7%)
	Heart disease	38.08	63.31	+66.3%	11.12	19.13	+72.1%	26.96	44.18	+63.7%
		(22.99, 56.23)	(44.74, 85.30)	(+53.5%, +91.4%)	(4.86, 19.81)	(11.68, 28.31)	(+47.0%, +132.1%)	(18.13, 36.42)	(33.06, 56.99)	(+55.4%, +81.6%)
	Malignant neoplasms	35.40	43.19	+23.2%	17.64	21.39	+22.9%	17.76	21.81	+23.5%
		(20.79, 51.76)	(27.98, 61.07)	(+16.5%, +34.6%)	(9.80, 26.45)	(13.78, 29.97)	(+12.8%, +40.1%)	(10.99, 25.30)	(14.20, 31.10)	(+18.1%, +32.6%)
	Chronic liver disease	35.91	83.15	+131.8%	17.89	43.05	+144.7%	18.02	40.10	+122.5%
	and cirrhosis	(22.11, 51.76)	(61.19, 109.18)	(+112.2%, +176.9%)	(9.58, 25.05)	(30.67, 56.42)	(+125.3%, +219.6%)	(12.52, 26.71)	(30.52, 52.76)	(+97.3%, +145.7%)
	Cerebrovascular diseases	3.71	5.35	+48.9%	1.92	2.11	-10.5%	1.79	3.24	+68.8%
		(0.13, 13.56)	(1.13, 14.35)	(0.0%, +257.8%)	(0.13, 8.31)	(0.56, 7.32)	(-33.9%, +72.0%)	(0.00, 5.25)	(0.56, 7.03)	(+18.3%, +1221.2%)
	Chronic lower respiratory diseases	0.26	0.84	+53.2%	0.00	0.14	-38.8%	0.26	0.70	+175.2%
		(0.00, 5.76)	(0.00, 6.61)	(-6.8%, +340.4%)	(0.00, 2.68)	(0.00, 2.39)	(-84.3%, +11.3%)	(0.00, 3.07)	(0.00, 4.22)	(+40.3%, +340.4%)
Non-Hispanic Asian	Drug overdose	3.22	11.80	+267.7%	1.14	3.08	+165.4%	2.08	8.73	+325.7%
		(1.11, 6.23)	(8.37, 15.50)	(+148.4%, +649.0%)	(0.03, 2.56)	(1.84, 4.48)	(+75.4%, +5618.9%)	(1.07, 3.67)	(6.53, 11.02)	(+199.3%, +508.8%)
	Unintentional injuries	19.93	14.49	-26.7%	7.58	4.31	-41.6%	12.35	10.18	-17.6%
	excluding drug overdose	(14.14, 26.08)	(10.92, 18.71)	(-28.9%, -21.7%)	(4.98, 10.31)	(2.96, 6.09)	(-43.6%, -38.6%)	(9.16, 15.77)	(7.96, 12.62)	(-20.2%, -11.8%)
	Suicide	10.48	14.95	+44.1%	2.70	4.26	+63.7%	7.78	10.68	+38.1%
	excluding drug overdose	(6.53, 14.60)	(11.25, 18.99)	(+30.3%, +72.6%)	(1.07, 4.32)	(2.77, 5.91)	(+35.2%, +154.2%)	(5.46, 10.27)	(8.49, 13.08)	(+28.2%, +57.2%)
	Homicide	7.02	3.93	-45.4%	2.53	1.52	-41.9%	4.50	2.42	-46.9%
	excluding drug overdose	(3.42, 10.79)	(1.82, 5.94)	(-48.9%, -43.4%)	(0.66, 4.19)	(0.57, 2.42)	(-52.6%, -25.2%)	(2.77, 6.61)	(1.25, 3.52)	(-53.6%, -45.1%)
	Heart disease	33.87	37.11	+9.2%	5.57	5.89	+2.1%	28.30	31.21	+10.2%
		(27.15, 41.69)	(31.50, 42.51)	(+1.8%, +16.1%)	(3.81, 8.27)	(4.11, 7.56)	(-8.5%, +9.9%)	(23.35, 33.42)	(27.39, 34.95)	(+4.4%, +17.3%)
	Malignant neoplasms	70.26	62.32	-11.6%	32.86	29.57	-10.7%	37.40	32.75	-12.5%
		(59.42, 82.24)	(54.56, 70.15)	(-14.7%, -8.1%)	(28.01, 38.51)	(25.67, 33.37)	(-13.5%, -7.8%)	(31.41, 43.73)	(28.88, 36.78)	(-15.7%, -8.1%)
	Chronic liver disease	3.39	5.25	+56.9%	0.55	1.43	+168.3%	2.84	3.82	+35.8%
	and cirrhosis	(1.25, 5.50)	(3.19, 7.67)	(+41.8%, +143.0%)	(0.00, 1.07)	(0.59, 2.33)	(+124.0%, +768.4%)	(1.25, 4.43)	(2.59, 5.34)	(+22.0%, +103.0%)
	Cerebrovascular diseases	11.45	9.74	-14.5%	4.91	3.54	-29.3%	6.54	6.20	-4.0%
		(7.89, 16.20)	(6.79, 12.84)	(-20.9%, -10.2%)	(3.36, 7.13)	(2.09, 4.75)	(-33.8%, -26.4%)	(4.53, 9.07)	(4.70, 8.09)	(-11.9%, +4.8%)
	Chronic lower respiratory diseases	2.14	1.03	-53.4%	0.66	0.31	-55.5%	1.49	0.73	-51.6%
		(0.59, 4.81)	(0.29, 2.46)	(-64.7%, -48.0%)	(0.00, 1.83)	(0.00, 1.10)	(-100.0%, -40.1%)	(0.59, 2.98)	(0.29, 1.36)	(-59.6%, -44.4%)

See footnotes at the end of table.

Supplementary Table 13: Leading causes of orphanhood among US children in 2019 by race and ethnicity and sex of parent.

LaTeX template

Race and ethnicity	Parental cause of death	Orphanhood * Incidence rate per 100,000 children			Maternal Orphanhood† Incidence rate per 100,000 children			Paternal Orphanhood‡ Incidence rate per 100,000 children		
		2000 rate, (95% UI)	2019 rate, (95% UI)	2000-2019 changes	2000 rate, (95% UI)	2019 rate, (95% UI)	2000-2019 changes	2000 rate, (95% UI)	2019 rate, (95% UI)	2000-2019 changes
Non-Hispanic Black	Drug overdose	18.15	57.15	+215.2%	6.99	18.80	+169.6%	11.15	38.35	+243.4%
		(15.43, 21.12)	(52.14, 62.31)	(+195.1%, +237.8%)	(5.67, 8.35)	(16.74, 20.95)	(+150.8%, +195.0%)	(9.76, 12.77)	(35.41, 41.36)	(+224.1%, +262.8%)
	Unintentional injuries	42.34	42.70	+1.2%	13.78	12.41	-9.6%	28.56	30.30	+6.3%
	excluding drug overdose	(37.80, 46.81)	(38.47, 47.28)	(+0.8%, +1.7%)	(11.69, 15.71)	(10.68, 14.21)	(-10.3%, -8.6%)	(26.11, 31.10)	(27.79, 33.07)	(+6.0%, +6.7%)
	Suicide	10.18	15.69	+54.1%	2.07	3.41	+66.0%	8.11	12.28	+51.4%
	excluding drug overdose	(8.19, 12.21)	(13.30, 18.15)	(+48.6%, +62.3%)	(1.35, 2.85)	(2.52, 4.30)	(+52.3%, +90.4%)	(6.84, 9.37)	(10.77, 13.86)	(+47.3%, +57.4%)
	Homicide	44.69	52.06	+16.6%	11.58	8.64	-25.7%	33.11	43.41	+31.3%
	excluding drug overdose	(40.38, 49.22)	(47.68, 56.69)	(+15.2%, +18.1%)	(9.85, 13.54)	(7.26, 10.18)	(-27.0%, -24.9%)	(30.53, 35.68)	(40.42, 46.51)	(+30.3%, +32.5%)
	Heart disease	89.47	97.53	+9.0%	32.57	29.19	-10.4%	56.90	68.34	+20.1%
		(83.18, 96.11)	(90.95, 103.99)	(+8.3%, +9.4%)	(29.67, 35.61)	(26.56, 31.82)	(-10.7%, -10.1%)	(53.51, 60.50)	(64.39, 72.17)	(+19.4%, +20.5%)
	Malignant neoplasms	86.37	69.85	-19.1%	42.09	32.11	-23.8%	44.28	37.74	-14.8%
		(79.99, 92.64)	(64.60, 75.26)	(-19.5%, -18.7%)	(38.74, 45.37)	(29.49, 34.77)	(-24.1%, -23.3%)	(41.24, 47.27)	(35.11, 40.49)	(-15.2%, -14.3%)
	Chronic liver disease	9.80	7.59	-22.5%	3.53	3.02	-15.1%	6.26	4.57	-27.1%
	and cirrhosis	(8.08, 11.89)	(6.08, 9.26)	(-24.8%, -20.9%)	(2.74, 4.57)	(2.28, 3.80)	(-17.6%, -13.7%)	(5.34, 7.32)	(3.80, 5.46)	(-29.5%, -23.6%)
	Cerebrovascular diseases	21.05	16.70	-20.7%	9.97	6.21	-37.5%	11.09	10.49	-5.9%
		(18.26, 23.94)	(14.28, 19.29)	(-21.8%, -19.7%)	(8.49, 11.55)	(5.11, 7.45)	(-39.8%, -35.7%)	(9.77, 12.39)	(9.16, 11.84)	(-6.6%, -5.0%)
	Chronic lower respiratory diseases	7.95	8.26	+3.6%	3.64	3.34	-8.5%	4.31	4.91	+13.5%
		(6.03, 10.04)	(6.51, 10.03)	(-0.2%, +7.5%)	(2.69, 4.68)	(2.52, 4.19)	(-11.0%, -6.4%)	(3.34, 5.35)	(4.00, 5.84)	(+8.6%, +19.3%)
Hispanic	Drug overdose	13.66	34.40	+151.9%	3.13	8.64	+175.8%	10.53	25.76	+144.8%
		(11.18, 16.39)	(31.26, 37.75)	(+130.4%, +179.6%)	(2.20, 4.15)	(7.43, 9.96)	(+139.6%, +238.5%)	(8.98, 12.23)	(23.83, 27.78)	(+127.1%, +165.4%)
	Unintentional injuries	40.07	32.44	-19.0%	9.62	7.91	-17.7%	30.45	24.53	-19.5%
	excluding drug overdose	(35.68, 44.68)	(29.42, 35.74)	(-20.0%, -17.6%)	(7.89, 11.34)	(6.79, 9.19)	(-19.2%, -13.7%)	(27.79, 33.34)	(22.63, 26.55)	(-20.4%, -18.6%)
	Suicide	10.86	14.99	+37.9%	1.72	3.27	+90.6%	9.14	11.72	+27.9%
	excluding drug overdose	(8.71, 13.23)	(12.93, 17.16)	(+29.6%, +48.3%)	(1.03, 2.51)	(2.57, 4.10)	(+62.4%, +150.1%)	(7.68, 10.73)	(10.37, 13.05)	(+21.8%, +34.9%)
	Homicide	18.36	11.92	-35.0%	4.02	2.73	-32.1%	14.34	9.19	-35.9%
	excluding drug overdose	(15.48, 21.51)	(10.16, 13.82)	(-35.6%, -34.4%)	(2.98, 5.25)	(2.08, 3.45)	(-34.2%, -30.1%)	(12.50, 16.25)	(8.08, 10.37)	(-36.1%, -35.3%)
	Heart disease	27.43	29.62	+8.0%	6.64	6.53	-1.4%	20.79	23.09	+11.1%
		(23.96, 31.03)	(26.95, 32.53)	(+4.8%, +12.6%)	(5.27, 8.06)	(5.58, 7.65)	(-5.1%, +5.8%)	(18.69, 22.98)	(21.37, 24.88)	(+8.3%, +14.7%)
	Malignant neoplasms	42.07	43.45	+3.3%	21.60	22.27	+3.2%	20.47	21.18	+3.4%
		(37.39, 46.91)	(39.83, 47.27)	(+0.7%, +6.5%)	(19.12, 24.17)	(20.39, 24.32)	(+0.6%, +6.5%)	(18.28, 22.74)	(19.44, 22.95)	(+0.8%, +6.5%)
	Chronic liver disease	13.02	14.73	+13.2%	2.26	3.44	+52.8%	10.76	11.29	+4.9%
	and cirrhosis	(10.89, 15.47)	(12.81, 16.72)	(+8.1%, +17.5%)	(1.59, 3.08)	(2.74, 4.23)	(+36.9%, +71.5%)	(9.30, 12.39)	(10.08, 12.49)	(+1.0%, +8.3%)
	Cerebrovascular diseases	8.21	7.66	-6.5%	3.42	2.66	-22.0%	4.79	5.00	+4.5%
		(6.27, 10.44)	(6.29, 9.29)	(-11.2%, -0.5%)	(2.54, 4.43)	(2.09, 3.38)	(-24.6%, -17.6%)	(3.74, 6.02)	(4.20, 5.91)	(-1.9%, +11.8%)
	Chronic lower respiratory diseases	1.69	1.75	+3.6%	0.79	0.75	-5.0%	0.90	1.00	+11.2%
		(0.85, 2.76)	(1.07, 2.52)	(-8.2%, +28.1%)	(0.38, 1.34)	(0.37, 1.11)	(-15.8%, +15.5%)	(0.47, 1.42)	(0.70, 1.41)	(-0.9%, +53.6%)

See footnotes at the end of table.

Supplementary Table 13: Leading causes of orphanhood among US children in 2019 by race and ethnicity and sex of parent.

Race and ethnicity	Parental cause of death	Orphanhood* Incidence rate per 100,000 children			Maternal Orphanhood† Incidence rate per 100,000 children			Paternal Orphanhood‡ Incidence rate per 100,000 children		
		2000 rate, (95% UI)	2019 rate, (95% UI)	2000-2019 changes	2000 rate, (95% UI)	2019 rate, (95% UI)	2000-2019 changes	2000 rate, (95% UI)	2019 rate, (95% UI)	2000-2019 changes
non-Hispanic white	Drug overdose	23.24	88.64	+281.4%	8.15	31.99	+292.2%	15.08	56.65	+275.6%
		(21.77, 24.77)	(85.29, 91.94)	(+271.1%, +291.8%)	(7.51, 8.83)	(30.50, 33.46)	(+279.0%, +306.3%)	(14.26, 15.94)	(54.80, 58.48)	(+266.8%, +284.0%)
	Unintentional injuries	46.33	42.30	-8.7%	13.02	12.02	-7.7%	33.31	30.27	-9.1%
	excluding drug overdose	(44.15, 48.55)	(40.09, 44.56)	(-9.1%, -8.3%)	(12.15, 13.89)	(11.11, 12.96)	(-8.5%, -6.8%)	(32.00, 34.66)	(28.98, 31.61)	(-9.4%, -8.8%)
	Suicide	25.35	39.36	+55.3%	4.58	8.29	+80.8%	20.77	31.07	+49.6%
	excluding drug overdose	(23.82, 26.96)	(37.25, 41.55)	(+54.1%, +56.3%)	(4.09, 5.11)	(7.53, 9.06)	(+77.2%, +84.1%)	(19.73, 21.86)	(29.72, 32.50)	(+48.7%, +50.5%)
	Homicide	7.20	7.07	-1.9%	2.72	2.36	-13.4%	4.48	4.71	+5.0%
	excluding drug overdose	(6.36, 8.07)	(6.15, 8.00)	(-3.2%, -1.0%)	(2.34, 3.12)	(1.98, 2.76)	(-15.6%, -11.8%)	(4.01, 4.95)	(4.17, 5.24)	(+4.0%, +5.8%)
	Heart disease	63.78	65.16	+2.2%	13.51	16.58	+22.6%	50.28	48.59	-3.4%
		(61.47, 66.16)	(62.58, 67.81)	(+1.8%, +2.5%)	(12.73, 14.32)	(15.61, 17.54)	(+22.4%, +22.9%)	(48.74, 51.84)	(46.98, 50.27)	(-3.6%, -3.0%)
	Malignant neoplasms	83.54	74.37	-11.0%	37.36	33.41	-10.6%	46.19	40.97	-11.3%
		(80.71, 86.44)	(71.59, 77.21)	(-11.3%, -10.6%)	(36.00, 38.77)	(32.05, 34.84)	(-11.0%, -10.1%)	(44.71, 47.68)	(39.54, 42.38)	(-11.6%, -11.0%)
	Chronic liver disease	12.78	19.42	+52.1%	3.40	6.91	+103.8%	9.38	12.51	+33.4%
	and cirrhosis	(11.81, 13.79)	(18.05, 20.90)	(+51.5%, +53.0%)	(3.02, 3.80)	(6.32, 7.56)	(+99.1%, +109.4%)	(8.79, 9.99)	(11.73, 13.34)	(+33.1%, +33.9%)
	Cerebrovascular diseases	9.18	8.93	-2.6%	3.79	3.49	-8.1%	5.38	5.45	+1.4%
		(8.27, 10.09)	(8.02, 9.90)	(-3.1%, -1.7%)	(3.38, 4.24)	(3.06, 3.92)	(-9.2%, -6.9%)	(4.89, 5.86)	(4.95, 5.98)	(+0.9%, +2.1%)
	Chronic lower respiratory diseases	5.70	8.23	+44.4%	2.14	3.41	+59.0%	3.56	4.82	+35.7%
		(5.05, 6.35)	(7.43, 9.01)	(+41.8%, +46.9%)	(1.82, 2.46)	(3.03, 3.79)	(+55.1%, +65.3%)	(3.23, 3.90)	(4.40, 5.22)	(+33.7%, +37.2%)

*: Orphanhood – "Children experiencing the death of one or both parents; Eq. (7)"

†: Maternal orphanhood – "Children experiencing the death of their mother, including children experiencing the death of both their mother and father; Eq. (9)"

‡: Paternal orphanhood – "Children experiencing the death of their father, including children experiencing the death of both their father and mother; Eq. (9)"

Supplementary Table 13: Leading causes of orphanhood among US children in 2019 by race and ethnicity and sex of parent (continued).

LaTeX template

	Orphanhood Incidence in 2021				Orphanhood Prevalence in 2021			
**US state**[‖]	**Incidence** (n, 95% uncertainty interval)	**Incidence rate per 100 children** (rate, 95% uncertainty interval)	**First ranked cause** (name, % contribution)	**Second ranked cause** (name, % contribution)	**Prevalence** (n, 95%) uncertainty interval)	**Prevalence rate per 100 children** (rate, 95% uncertainty interval)	**First ranked cause** (name, % contribution)	**Second ranked cause** (name, % contribution)
Alabama	8,006 (6,312, 9,886)	0.71 (0.56, 0.88)	Heart disease 16.3%	COVID-19 16.2%	44,842 (33,750, 58,038)	4.00 (3.01, 5.17)	Heart disease 17.0%	Unintentional injuries excluding drug overdose 14.0%
Alaska	1,103 (610, 1,932)	0.61 (0.34, 1.08)	Drug overdose 16.0%	Unintentional injuries excluding drug overdose 14.7%	5,784 (2,906, 12,106)	3.22 (1.62, 6.75)	Unintentional injuries excluding drug overdose 23.9%	Suicide excluding drug overdose 10.9%
Arizona	10,978 (8,948, 13,308)	0.68 (0.55, 0.82)	COVID-19 17.8%	Drug overdose 17.0%	58,592 (44,874, 74,683)	3.63 (2.78, 4.63)	Drug overdose 16.7%	Unintentional injuries excluding drug overdose 12.9%
Arkansas	4,474 (3,259, 5,958)	0.64 (0.46, 0.85)	Heart disease 15.3%	COVID-19 13.2%	25,246 (17,292, 35,399)	3.59 (2.46, 5.03)	Unintentional injuries excluding drug overdose 16.7%	Heart disease 15.5%
California	45,422 (41,255, 49,685)	0.52 (0.47, 0.57)	COVID-19 16.4%	Drug overdose 15.0%	263,472 (233,794, 294,797)	3.00 (2.67, 3.36)	Malignant neoplasms 16.9%	Heart disease 12.9%
Colorado	6,600 (5,113, 8,345)	0.53 (0.41, 0.67)	Drug overdose 18.4%	COVID-19 10.3%	37,739 (27,398, 50,079)	3.04 (2.20, 4.03)	Drug overdose 16.7%	Suicide excluding drug overdose 13.8%
Connecticut	3,340 (2,381, 4,440)	0.46 (0.33, 0.61)	Drug overdose 29.4%	Malignant neoplasms 13.1%	20,860 (14,148, 29,067)	2.86 (1.94, 3.98)	Drug overdose 26.7%	Malignant neoplasms 14.7%
Delaware	963 (535, 1,573)	0.46 (0.26, 0.76)	Drug overdose 34.9%	Malignant neoplasms 9.4%	5,361 (2,630, 9,911)	2.57 (1.26, 4.76)	Drug overdose 29.3%	Unintentional injuries excluding drug overdose 10.2%
District of Columbia	624 (337, 1,005)	0.50 (0.27, 0.80)	Drug overdose 18.1%	Homicide excluding drug overdose 10.1%	3,806 (1,747, 6,465)	3.02 (1.39, 5.14)	Heart disease 13.0%	Drug overdose 12.2%
Florida	28,758 (25,532, 32,149)	0.67 (0.60, 0.75)	Drug overdose 19.3%	COVID-19 17.8%	158,570 (136,729, 182,303)	3.70 (3.19, 4.25)	Drug overdose 18.4%	Malignant neoplasms 14.3%
Georgia	15,808 (13,325, 18,526)	0.63 (0.53, 0.73)	COVID-19 16.5%	Heart disease 15.3%	86,644 (69,907, 105,222)	3.43 (2.77, 4.17)	Heart disease 15.7%	Malignant neoplasms 13.2%

See footnotes at the end of table.

Supplementary Table 14: Leading causes of orphanhood among US children in 2021 by US state.

	Orphanhood Incidence in 2021				Orphanhood Prevalence in 2021			
**US state**[‖]	**Incidence (n, 95% uncertainty interval)**	**Incidence rate per 100 children (rate, 95% uncertainty interval)**	**First ranked cause (name, % contribution)**	**Second ranked cause (name, % contribution)**	**Prevalence (n, 95%) uncertainty interval)**	**Prevalence rate per 100 children (rate, 95% uncertainty interval)**	**First ranked cause (name, % contribution)**	**Second ranked cause (name, % contribution)**
Hawaii	1,218 (744, 1,938)	0.40 (0.24, 0.64)	Malignant neoplasms 16.6%	Heart disease 15.7%	7,572 (4,053, 13,296)	2.49 (1.33, 4.37)	Malignant neoplasms 15.3%	Heart disease 15.3%
Idaho	1,945 (1,186, 2,963)	0.41 (0.25, 0.63)	COVID-19 14.6%	Drug overdose 12.9%	10,372 (5,642, 18,139)	2.21 (1.20, 3.87)	Unintentional injuries excluding drug overdose 20.5%	Suicide excluding drug overdose 13.3%
Illinois	13,960 (11,678, 16,409)	0.50 (0.42, 0.59)	Drug overdose 16.8%	Heart disease 14.1%	89,434 (72,544, 108,037)	3.19 (2.59, 3.85)	Drug overdose 16.6%	Malignant neoplasms 15.5%
Indiana	9,204 (7,319, 11,243)	0.58 (0.46, 0.71)	Drug overdose 23.2%	Heart disease 11.9%	54,354 (41,559, 69,327)	3.42 (2.62, 4.37)	Drug overdose 20.6%	Heart disease 13.1%
Iowa	2,776 (1,898, 3,918)	0.38 (0.26, 0.53)	Heart disease 14.5%	Malignant neoplasms 12.4%	16,491 (10,524, 25,000)	2.24 (1.43, 3.39)	Unintentional injuries excluding drug overdose 15.9%	Malignant neoplasms 14.3%
Kansas	3,488 (2,371, 4,824)	0.50 (0.34, 0.69)	Drug overdose 15.0%	COVID-19 13.0%	19,704 (12,710, 29,423)	2.80 (1.81, 4.18)	Unintentional injuries excluding drug overdose 15.9%	Malignant neoplasms 12.4%
Kentucky	7,238 (5,714, 8,994)	0.71 (0.56, 0.89)	Drug overdose 24.9%	Heart disease 13.8%	41,655 (31,353, 54,148)	4.10 (3.09, 5.33)	Drug overdose 23.3%	Heart disease 14.0%
Louisiana	8,172 (6,473, 10,058)	0.75 (0.60, 0.93)	Drug overdose 23.9%	Heart disease 12.5%	45,957 (34,581, 59,512)	4.24 (3.19, 5.50)	Drug overdose 17.7%	Heart disease 14.2%
Maine	1,379 (844, 2,029)	0.55 (0.33, 0.81)	Drug overdose 30.5%	Heart disease 10.4%	7,235 (3,784, 11,981)	2.87 (1.50, 4.76)	Drug overdose 26.7%	Unintentional injuries excluding drug overdose 13.9%
Maryland	7,472 (5,893, 9,225)	0.55 (0.43, 0.68)	Drug overdose 23.9%	Heart disease 13.6%	47,860 (36,414, 61,059)	3.51 (2.67, 4.48)	Drug overdose 23.0%	Heart disease 14.6%
Massachusetts	6,108 (4,815, 7,571)	0.45 (0.35, 0.56)	Drug overdose 28.0%	Malignant neoplasms 14.6%	41,334 (31,355, 52,807)	3.03 (2.30, 3.88)	Drug overdose 27.6%	Malignant neoplasms 15.7%
Michigan	12,256 (10,186, 14,589)	0.57 (0.47, 0.68)	Drug overdose 17.4%	Heart disease 13.6%	74,604 (59,491, 91,589)	3.46 (2.76, 4.25)	Drug overdose 18.1%	Heart disease 14.5%

See footnotes at the end of table.

Supplementary Table 14: Leading causes of orphanhood among US children in 2021 by US state.

LaTeX template

US state[‖]	Orphanhood Incidence in 2021: Incidence (n, 95% uncertainty interval)	Incidence rate per 100 children (rate, 95% uncertainty interval)	First ranked cause (name, % contribution)	Second ranked cause (name, % contribution)	Orphanhood Prevalence in 2021: Prevalence (n, 95%) uncertainty interval)	Prevalence rate per 100 children (rate, 95% uncertainty interval)	First ranked cause (name, % contribution)	Second ranked cause (name, % contribution)
Minnesota	5,463 (4,050, 7,039)	0.41 (0.31, 0.53)	Drug overdose 17.7%	Malignant neoplasms 13.7%	30,401 (21,374, 41,688)	2.31 (1.62, 3.16)	Malignant neoplasms 16.7%	Drug overdose 14.7%
Mississippi	5,239 (3,905, 6,843)	0.76 (0.56, 0.99)	COVID-19 15.0%	Heart disease 14.7%	29,612 (21,050, 40,550)	4.27 (3.04, 5.85)	Unintentional injuries excluding drug overdose 16.9%	Heart disease 15.6%
Missouri	8,162 (6,501, 10,053)	0.59 (0.47, 0.73)	Drug overdose 18.9%	Heart disease 13.4%	49,053 (37,188, 62,962)	3.54 (2.69, 4.55)	Drug overdose 18.5%	Heart disease 14.0%
Montana	1,210 (697, 2,015)	0.51 (0.30, 0.86)	Unintentional injuries excluding drug overdose 18.1%	Suicide excluding drug overdose 10.8%	6,387 (3,236, 12,544)	2.72 (1.38, 5.34)	Unintentional injuries excluding drug overdose 25.0%	Suicide excluding drug overdose 13.0%
Nebraska	1,805 (1,086, 2,810)	0.37 (0.22, 0.58)	Malignant neoplasms 13.5%	Unintentional injuries excluding drug overdose 12.1%	10,511 (6,037, 18,121)	2.18 (1.25, 3.75)	Unintentional injuries excluding drug overdose 18.8%	Malignant neoplasms 14.1%
Nevada	4,054 (2,913, 5,399)	0.58 (0.42, 0.77)	COVID-19 19.1%	Heart disease 13.6%	21,742 (14,418, 31,050)	3.11 (2.06, 4.44)	Drug overdose 15.6%	Heart disease 14.8%
New Hampshire	1,080 (634, 1,694)	0.42 (0.25, 0.66)	Drug overdose 25.3%	Malignant neoplasms 11.4%	6,792 (3,587, 11,720)	2.65 (1.40, 4.57)	Drug overdose 30.7%	Malignant neoplasms 11.3%
New Jersey	9,794 (7,958, 11,816)	0.48 (0.39, 0.58)	Drug overdose 22.3%	Malignant neoplasms 13.8%	64,008 (50,326, 79,466)	3.16 (2.49, 3.93)	Drug overdose 21.6%	Malignant neoplasms 15.7%
New Mexico	4,154 (2,951, 5,527)	0.88 (0.62, 1.17)	Drug overdose 16.9%	COVID-19 15.9%	21,418 (13,994, 31,009)	4.53 (2.96, 6.55)	Drug overdose 17.7%	Unintentional injuries excluding drug overdose 15.2%
New York	19,384 (16,829, 22,115)	0.47 (0.41, 0.54)	Drug overdose 19.6%	Heart disease 14.7%	124,204 (105,602, 144,622)	3.02 (2.57, 3.52)	Malignant neoplasms 17.8%	Drug overdose 16.1%
North Carolina	14,166 (11,917, 16,660)	0.62 (0.52, 0.72)	Drug overdose 21.1%	COVID-19 12.5%	78,268 (62,890, 95,571)	3.40 (2.73, 4.15)	Drug overdose 18.0%	Malignant neoplasms 12.6%
North Dakota	671 (340, 1,366)	0.36 (0.18, 0.74)	Unintentional injuries excluding drug overdose 15.4%	Drug overdose 10.3%	3,750 (1,760, 8,793)	2.02 (0.95, 4.74)	Unintentional injuries excluding drug overdose 18.1%	Suicide excluding drug overdose 7.8%
Ohio	15,912 (13,558, 18,525)	0.61 (0.52, 0.71)	Drug overdose 25.4%	Heart disease 12.2%	96,450 (79,472, 115,385)	3.70 (3.05, 4.43)	Drug overdose 26.4%	Heart disease 12.9%

See footnotes at the end of table.

Supplementary Table 14: Leading causes of orphanhood among US children in 2021 by US state.

	Orphanhood Incidence in 2021				Orphanhood Prevalence in 2021			
US state[‖]	Incidence (n, 95% uncertainty interval)	Incidence rate per 100 children (rate, 95% uncertainty interval)	First ranked cause (name, % contribution)	Second ranked cause (name, % contribution)	Prevalence (n, 95%) uncertainty interval)	Prevalence rate per 100 children (rate, 95% uncertainty interval)	First ranked cause (name, % contribution)	Second ranked cause (name, % contribution)
Oklahoma	6,142 (4,684, 7,880)	0.64 (0.49, 0.82)	COVID-19 16.8%	Heart disease 14.4%	35,776 (25,602, 48,352)	3.72 (2.66, 5.03)	Unintentional injuries excluding drug overdose 14.9%	Heart disease 14.8%
Oregon	4,193 (3,027, 5,457)	0.49 (0.35, 0.63)	Drug overdose 16.1%	Malignant neoplasms 13.2%	22,862 (15,511, 31,690)	2.65 (1.80, 3.68)	Malignant neoplasms 14.8%	Unintentional injuries excluding drug overdose 13.7%
Pennsylvania	14,822 (12,520, 17,258)	0.55 (0.47, 0.65)	Drug overdose 25.2%	Malignant neoplasms 12.0%	95,322 (78,581, 113,803)	3.56 (2.94, 4.26)	Drug overdose 26.5%	Malignant neoplasms 13.5%
Rhode Island	791 (443, 1,246)	0.38 (0.21, 0.60)	Drug overdose 35.4%	Heart disease 9.7%	4,792 (2,256, 8,358)	2.29 (1.08, 4.00)	Drug overdose 29.8%	Malignant neoplasms 11.2%
South Carolina	7,321 (5,749, 9,077)	0.66 (0.51, 0.81)	Drug overdose 18.7%	COVID-19 14.6%	39,090 (29,200, 50,878)	3.50 (2.61, 4.55)	Drug overdose 15.2%	Unintentional injuries excluding drug overdose 14.8%
South Dakota	1,074 (588, 1,962)	0.49 (0.27, 0.89)	Chronic liver disease and cirrhosis 17.2%	Unintentional injuries excluding drug overdose 11.8%	5,385 (2,624, 11,990)	2.44 (1.19, 5.44)	Unintentional injuries excluding drug overdose 19.9%	Suicide excluding drug overdose 8.5%
Tennessee	12,006 (9,896, 14,281)	0.78 (0.64, 0.93)	Drug overdose 23.8%	COVID-19 13.4%	62,657 (49,128, 78,298)	4.07 (3.19, 5.08)	Drug overdose 19.0%	Heart disease 14.5%
Texas	40,518 (36,402, 44,832)	0.54 (0.49, 0.60)	COVID-19 23.1%	Heart disease 12.0%	222,077 (193,884, 252,286)	2.97 (2.59, 3.37)	Malignant neoplasms 14.0%	Heart disease 13.8%
Utah	3,770 (2,585, 5,271)	0.40 (0.27, 0.56)	Drug overdose 14.9%	COVID-19 14.1%	24,352 (15,774, 35,974)	2.57 (1.67, 3.80)	Drug overdose 20.1%	Suicide excluding drug overdose 16.1%
Vermont	470 (229, 824)	0.40 (0.20, 0.70)	Drug overdose 32.6%	Unintentional injuries excluding drug overdose 10.2%	2,369 (818, 4,934)	2.03 (0.70, 4.22)	Drug overdose 19.5%	Unintentional injuries excluding drug overdose 11.6%
Virginia	9,712 (7,902, 11,782)	0.52 (0.42, 0.63)	Drug overdose 18.9%	Heart disease 13.8%	56,662 (43,957, 71,417)	3.01 (2.33, 3.79)	Drug overdose 16.4%	Malignant neoplasms 15.3%

See footnotes at the end of table.

Supplementary Table 14: Leading causes of orphanhood among US children in 2021 by US state.

LaTeX template

US state[‖]	Orphanhood Incidence in 2021: Incidence (n, 95% uncertainty interval)	Incidence rate per 100 children (rate, 95% uncertainty interval)	First ranked cause (name, % contribution)	Second ranked cause (name, % contribution)	Orphanhood Prevalence in 2021: Prevalence (n, 95%) uncertainty interval)	Prevalence rate per 100 children (rate, 95% uncertainty interval)	First ranked cause (name, % contribution)	Second ranked cause (name, % contribution)
Washington	7,492 (5,852, 9,290)	0.45 (0.35, 0.55)	Drug overdose 18.1%	Malignant neoplasms 13.4%	42,628 (31,616, 55,479)	2.54 (1.89, 3.31)	Drug overdose 15.9%	Malignant neoplasms 15.5%
West Virginia	3,175 (2,253, 4,252)	0.88 (0.63, 1.18)	Drug overdose 35.9%	COVID-19 10.6%	17,754 (11,778, 25,574)	4.94 (3.28, 7.12)	Drug overdose 33.2%	Unintentional injuries excluding drug overdose 11.3%
Wisconsin	4,946 (3,779, 6,333)	0.39 (0.30, 0.50)	Drug overdose 22.0%	Malignant neoplasms 11.9%	29,588 (21,354, 39,631)	2.32 (1.68, 3.11)	Drug overdose 20.0%	Malignant neoplasms 14.6%
Wyoming	542 (250, 1,096)	0.41 (0.19, 0.83)	Unintentional injuries excluding drug overdose 13.5%	Suicide excluding drug overdose 10.5%	2,994 (1,143, 7,293)	2.26 (0.86, 5.51)	Unintentional injuries excluding drug overdose 25.6%	Suicide excluding drug overdose 11.6%

‖: Discrepancies between the sum of US state-level estimates and the national-level estimates in Table 2 are due to working from partially suppressed counts from CDC WONDER (see Methods).

Supplementary Table 14: Leading causes of orphanhood among US children in 2021 by US state (continued).

Rank*	US state	Primary parental cause of death	standardized race and ethnicity	Population size**	Deaths***	Exclusion criteria	Orphanhood prevalence rate per 100 children†
1	West Virginia	Drug overdose	Hispanic	16,747	3	small populations & small death counts	-
			Non-Hispanic American Indian or Alaska Native	3,265	0	small populations & small death counts	-
			Non-Hispanic Asian	11,802	0	small populations & small death counts	-
			Non-Hispanic Black	52,936	14	small death counts	-
			non-Hispanic white	1,425,205	605		1.91 (1.41,2.38)
2	New Mexico	Drug overdose	Hispanic	698,062	279		0.88 (0.22,1.66)
			Non-Hispanic American Indian or Alaska Native	135,495	14	small death counts	-
			Non-Hispanic Asian	25,511	2	small populations & small death counts	-
			Non-Hispanic Black	32,153	8	small death counts	-
			non-Hispanic white	709,715	211		0.77 (0.56,1.85)
3	Mississippi	Unintentional injuries excluding drug overdose	Hispanic	54,559	23	small death counts	-
			Non-Hispanic American Indian or Alaska Native	10,509	7	small populations & small death counts	-
			Non-Hispanic Asian	23,108	6	small death counts	-
			Non-Hispanic Black	827,308	450		0.85 (0.52,1.29)
			non-Hispanic white	1,413,502	921		0.81 (0.49,1.15)
4	Louisiana	Drug overdose	Hispanic	140,666	9	small death counts	-
			Non-Hispanic American Indian or Alaska Native	23,284	3	small death counts	-
			Non-Hispanic Asian	61,623	3	small death counts	-
			Non-Hispanic Black	1,121,505	244		0.79 (0.61,1.11)
			non-Hispanic white	2,275,684	593		0.85 (0.66,1.16)
5	Kentucky	Drug overdose	Hispanic	86,829	6	small death counts	-
			Non-Hispanic American Indian or Alaska Native	7,830	0	small populations & small death counts	-
			Non-Hispanic Asian	44,705	1	small death counts	-
			Non-Hispanic Black	268,423	42	small death counts	-
			non-Hispanic white	3,066,947	973		1.18 (0.97,1.39)

See footnotes at the end of table.

Supplementary Table 15: Race and ethnicity groups most affected by the leading parental cause of death in the ten US states with highest orphanhood prevalence rates in 2021.

LaTeX template

Rank*	US state	Primary parental cause of death	standardized race and ethnicity	Population size**	Deaths***	Exclusion criteria	Orphanhood prevalence rate per 100 children†
6	Tennessee	Drug overdose	Hispanic	188,570	11	small death counts	-
			Non-Hispanic American Indian or Alaska Native	15,235	2	small populations & small death counts	-
			Non-Hispanic Asian	82,733	3	small death counts	-
			Non-Hispanic Black	821,884	218		0.89 (0.70,1.24)
			non-Hispanic white	4,003,852	1,045		0.90 (0.74,1.07)
7	Alabama	Heart disease	Hispanic	113,163	34	small death counts	-
			Non-Hispanic American Indian or Alaska Native	22,716	14	small populations & small death counts	-
			Non-Hispanic Asian	48,085	14	small death counts	-
			Non-Hispanic Black	973,707	2,920		0.71 (0.49,1.01)
			non-Hispanic white	2,658,856	9,896		0.68 (0.54,0.83)
8	Oklahoma	Unintentional injuries excluding drug overdose	Hispanic	218,343	60	small death counts	-
			Non-Hispanic American Indian or Alaska Native	254,133	199		0.98 (0.70,1.92)
			Non-Hispanic Asian	63,860	11	small death counts	-
			Non-Hispanic Black	225,681	112		0.70 (0.46,1.82)
			non-Hispanic white	2,205,212	1,249		0.87 (0.62,1.15)
9	Ohio	Drug overdose	Hispanic	246,561	35	small death counts	-
			Non-Hispanic American Indian or Alaska Native	23,565	3	small populations & small death counts	-
			Non-Hispanic Asian	182,029	5	small death counts	-
			Non-Hispanic Black	1,104,289	394		0.97 (0.71,1.31)
			non-Hispanic white	7,747,715	1,941		1.19 (1.02,1.32)
10	Florida	Drug overdose	Hispanic	3,423,469	379	large discrepancy in estimates	-
			Non-Hispanic American Indian or Alaska Native	46,070	7	small death counts & large discrepancy in estimates	-
			Non-Hispanic Asian	422,975	10	small death counts & large discrepancy in estimates	-
			Non-Hispanic Black	2,271,752	326	large discrepancy in estimates	-
			non-Hispanic white	9,595,347	2,496	large discrepancy in estimates	-

*: US states ranked by highest orphanhood prevalence in 2021. **: Average population size of people older than 15 years between 2000 to 2021. ***: Average number of suppression-adjusted death counts between 1999 to 2021 by primary parental cause of death. †: Point estimate of orphanhood prevalence rate per 100 children in 2021 in the listed US state and standardized race and ethnicity along with 95% uncertainty ranges. '-' indicates that estimates could not be reliably estimated.

Supplementary Table 15: Race and ethnicity groups most affected by the primary parental cause of death in the ten US states with highest orphanhood prevalence rates in 2021 (continued).

Year	Data Source	Mortality data by leading parental cause of death and sex							
		COVID-19		Diseases of heart		Drug overdose		Homicide excluding drug overdose	
		**Men**	**Women**	**Men**	**Women**	**Men**	**Women**	**Men**	**Women**
2000	NCHS			344,236	365,520	11,504	5,825	12,163	3,470
	CDC WONDER			344,236	365,520	11,467	5,776	12,172	3,478
2005	NCHS			322,407	328,886	18,655	11,041	13,747	3,300
	CDC WONDER			322,407	328,886	18,605	10,985	13,768	3,306
2010	NCHS			306,982	290,006	22,923	15,273	12,164	3,081
	CDC WONDER			306,982	290,006	22,863	15,240	12,191	3,089
2011	NCHS			308,035	287,838	24,918	16,304	12,117	3,066
	CDC WONDER			308,035	287,838	24,856	16,255	12,154	3,083
2012	NCHS			312,092	286,917	25,042	16,344	12,627	3,062
	CDC WONDER			312,092	286,917	24,991	16,313	12,662	3,077
2013	NCHS			320,964	289,467	26,745	17,126	12,145	3,010
	CDC WONDER			320,964	289,467	26,660	17,081	12,180	3,021
2014	NCHS			324,692	288,956	28,759	18,181	11,938	2,916
	CDC WONDER			324,692	288,956	28,692	18,141	12,032	2,932
2015	NCHS			334,644	298,520	32,887	19,368	13,654	3,136
	CDC WONDER			334,644	298,520	32,820	19,323	13,691	3,149
2016	NCHS			338,932	295,694	41,483	21,997	14,918	3,462
	CDC WONDER			338,932	295,694	41,411	21,951	14,950	3,474
2017	NCHS			347,534	299,273	46,472	23,604	14,899	3,554
	CDC WONDER			347,534	299,273	46,410	23,548	14,931	3,582
2018	NCHS			354,075	300,694	44,871	22,362	14,233	3,592
	CDC WONDER			354,075	300,694	44,833	22,303	14,252	3,621
2019	NCHS			357,424	301,000	47,800	22,659	14,641	3,506
	CDC WONDER			357,424	301,000	47,736	22,606	14,676	3,533
2020	NCHS	192,447	158,277	382,496	313,920	63,592	27,944	19,228	4,207
	CDC WONDER	192,447	158,277	382,496	313,920	63,542	27,898	19,252	4,239
2021	NCHS	236,455	180,148	384,555	310,389	74,136	32,244	20,327	4,502
	CDC WONDER	236,455	180,148	384,555	310,389	74,086	32,170	20,349	4,535

Supplementary Table 16: Comparison of aggregated line-list US mortality data from NCHS to mortality counts from CDC WONDER by leading parental cause of death.

Year	Data Source	Mortality data by leading parental cause of death and sex							
		Unintentional injuries excluding drug overdose		Suicide excluding drug overdose		Malignant neoplasms		Others	
		Men	Women	Men	Women	Men	Women	Men	Women
2000	NCHS	51,935	28,525	21,842	3,965	285,220	266,334	427,446	534,583
	CDC WONDER	51,937	28,534	21,842	3,965	285,220	266,334	427,472	534,615
2005	NCHS	58,252	31,949	23,732	4,392	289,598	268,253	458,449	575,303
	CDC WONDER	58,257	31,956	23,738	4,392	289,598	268,253	458,467	575,346
2010	NCHS	54,561	32,199	27,539	5,256	300,322	273,092	488,638	602,092
	CDC WONDER	54,562	32,201	27,548	5,257	300,322	273,092	488,661	602,114
2011	NCHS	55,864	33,370	28,327	5,607	301,523	273,879	505,184	625,660
	CDC WONDER	55,867	33,379	28,332	5,610	301,523	273,879	505,201	625,680
2012	NCHS	56,620	33,971	28,972	5,852	304,896	276,344	514,803	632,543
	CDC WONDER	56,621	33,971	28,970	5,855	304,896	276,344	514,820	632,556
2013	NCHS	56,771	34,146	29,232	6,094	306,869	276,716	534,694	650,034
	CDC WONDER	56,772	34,149	29,242	6,098	306,869	276,716	534,733	650,061
2014	NCHS	58,355	35,146	30,376	6,550	310,593	279,868	545,232	652,398
	CDC WONDER	58,249	35,130	30,431	6,546	310,593	279,869	545,256	652,441
2015	NCHS	61,488	36,958	31,515	7,077	313,116	281,531	567,563	678,204
	CDC WONDER	61,490	36,965	31,511	7,072	313,116	281,531	567,595	678,234
2016	NCHS	64,253	38,250	32,333	7,117	313,836	282,871	576,116	680,140
	CDC WONDER	64,257	38,250	32,330	7,122	313,836	282,871	576,155	680,169
2017	NCHS	65,252	39,258	34,214	7,375	314,476	283,386	598,204	704,087
	CDC WONDER	65,257	39,258	34,209	7,383	314,476	283,386	598,234	704,107
2018	NCHS	65,012	39,421	35,305	7,629	314,889	283,156	612,583	710,492
	CDC WONDER	65,020	39,429	35,300	7,633	314,889	283,156	612,599	710,510
2019	NCHS	66,881	40,123	34,873	7,340	315,271	283,203	619,929	709,947
	CDC WONDER	66,888	40,131	34,874	7,334	315,271	283,203	619,950	709,971
2020	NCHS	71,610	41,960	34,199	6,882	317,127	284,067	672,859	764,064
	CDC WONDER	71,615	41,962	34,199	6,875	317,127	284,067	672,880	764,083
2021	NCHS	77,322	45,110	36,040	7,188	318,036	286,040	674,659	747,276
	CDC WONDER	77,336	45,118	36,043	7,183	318,036	286,040	674,670	747,314

Supplementary Table 16: Comparison of aggregated line-list US mortality data from NCHS to mortality counts from CDC WONDER by leading parental cause of death (continued).

	Before the COVID-19 pandemic					Since the COVID-19 pandemic	
	**2000**	**2005**	**2010**	**2015**	**2019**	**2020**	**2021**
**Incidence (n, (95% uncertainty interval))**							
COVID-19	0	0	0	0	0	26,186	56,956
	(0, 0)	(0, 0)	(0, 0)	(0, 0)	(0, 0)	(24,050, 28,455)	(53,672, 60,327)
Drug overdose	14,395	21,979	25,736	35,037	47,664	62,722	71,065
	(12,994, 15,940)	(20,205, 23,857)	(23,815, 27,716)	(32,780, 37,420)	(44,922, 50,466)	(59,567, 66,060)	(67,652, 74,602)
Unintentional injuries	31,861	32,856	27,040	27,565	28,251	31,572	33,709
excluding drug overdose	(29,453, 34,312)	(30,420, 35,384)	(24,844, 29,360)	(25,361, 29,920)	(26,037, 30,645)	(29,162, 34,070)	(31,277, 36,219)
Suicide	14,388	15,208	16,887	19,052	20,548	19,934	20,487
excluding drug overdose	(12,994, 15,857)	(13,763, 16,740)	(15,339, 18,507)	(17,395, 20,776)	(18,804, 22,381)	(18,219, 21,675)	(18,776, 22,301)
Homicide	10,862	11,366	9,718	10,480	11,074	14,133	14,906
excluding drug overdose	(9,462, 12,354)	(9,985, 12,895)	(8,398, 11,045)	(9,173, 11,875)	(9,712, 12,488)	(12,623, 15,694)	(13,382, 16,491)
Malignant neoplasms	54,858	53,897	51,508	49,995	47,336	46,800	46,327
	(51,857, 57,943)	(50,992, 56,980)	(48,621, 54,484)	(47,125, 52,941)	(44,559, 50,216)	(44,049, 49,652)	(43,561, 49,152)
Heart disease	43,452	44,482	41,571	42,877	43,333	48,777	49,429
	(40,962, 46,083)	(41,912, 47,199)	(39,126, 44,183)	(40,324, 45,596)	(40,734, 46,003)	(45,992, 51,666)	(46,648, 52,288)
Other	92,220	90,963	81,908	88,125	93,912	110,068	114,498
	(79,400, 107,919)	(78,245, 106,435)	(69,779, 96,700)	(75,424, 103,384)	(80,785, 109,709)	(95,717, 127,098)	(99,936, 131,495)
**Incidence rate per 100,000 children (rate, (95% uncertainty interval))**							
COVID-19	0.00	0.00	0.00	0.00	0.00	35.96	82.07
	(0.00, 0.00)	(0.00, 0.00)	(0.00, 0.00)	(0.00, 0.00)	(0.00, 0.00)	(33.03, 39.07)	(77.34, 86.93)
Drug overdose	19.89	29.89	34.72	47.57	65.21	86.13	102.41
	(17.95, 22.02)	(27.48, 32.45)	(32.13, 37.39)	(44.51, 50.81)	(61.46, 69.05)	(81.80, 90.71)	(97.49, 107.50)
Unintentional injuries	44.02	44.69	36.48	37.43	38.65	43.35	48.58
excluding drug overdose	(40.69, 47.41)	(41.37, 48.13)	(33.52, 39.61)	(34.43, 40.62)	(35.62, 41.93)	(40.05, 46.79)	(45.07, 52.19)
Suicide	19.88	20.68	22.78	25.87	28.11	27.37	29.52
excluding drug overdose	(17.95, 21.91)	(18.72, 22.77)	(20.69, 24.97)	(23.62, 28.21)	(25.73, 30.62)	(25.02, 29.76)	(27.06, 32.14)
Homicide	15.01	15.46	13.11	14.23	15.15	19.41	21.48
excluding drug overdose	(13.07, 17.07)	(13.58, 17.54)	(11.33, 14.90)	(12.45, 16.12)	(13.29, 17.09)	(17.33, 21.55)	(19.28, 23.76)
Malignant neoplasms	75.80	73.31	69.49	67.88	64.77	64.27	66.76
	(71.65, 80.06)	(69.35, 77.50)	(65.60, 73.50)	(63.98, 71.88)	(60.97, 68.71)	(60.49, 68.18)	(62.77, 70.83)
Heart disease	60.04	60.50	56.08	58.21	59.29	66.98	71.23
	(56.60, 63.67)	(57.01, 64.20)	(52.79, 59.61)	(54.75, 61.91)	(55.73, 62.94)	(63.16, 70.95)	(67.22, 75.35)
Other	127.42	123.72	110.50	119.65	128.49	151.15	164.99
	(109.70, 149.11)	(106.42, 144.76)	(94.14, 130.46)	(102.40, 140.37)	(110.53, 150.10)	(131.44, 174.53)	(144.01, 189.49)
**Prevalence (n, (95 uncertainty interval))**							
COVID-19	0	0	0	0	0	25,954	79,626
	(0, 0)	(0, 0)	(0, 0)	(0, 0)	(0, 0)	(23,837, 28,203)	(74,425, 85,032)
Drug overdose	104,515	140,358	182,710	234,233	318,343	351,770	390,520
	(92,631, 117,383)	(126,750, 154,831)	(167,348, 199,010)	(216,587, 252,736)	(297,536, 340,037)	(329,876, 374,659)	(367,399, 414,695)
Unintentional injuries	340,900	318,203	292,485	272,136	267,170	269,135	272,871
excluding drug overdose	(316,946, 365,940)	(294,943, 342,477)	(270,216, 315,645)	(250,536, 294,675)	(245,735, 289,641)	(247,610, 291,663)	(251,255, 295,441)
Suicide	147,622	143,650	143,630	155,122	170,432	173,195	176,021
excluding drug overdose	(134,091, 162,099)	(130,280, 157,923)	(130,186, 158,028)	(140,832, 170,357)	(155,158, 186,616)	(157,733, 189,499)	(160,410, 192,475)
Homicide	150,713	133,263	115,967	107,984	109,865	113,260	117,231
excluding drug overdose	(134,644, 167,641)	(118,413, 149,114)	(102,314, 130,464)	(94,944, 121,883)	(96,757, 123,808)	(99,980, 127,319)	(103,791, 131,408)
Malignant neoplasms	379,696	371,532	353,627	346,036	335,004	331,588	327,357
	(355,721, 404,922)	(347,881, 396,464)	(330,596, 377,690)	(323,037, 370,050)	(312,165, 358,765)	(308,858, 355,211)	(304,749, 350,830)
Heart disease	303,371	305,626	296,453	297,452	301,179	306,993	312,113
	(282,953, 324,866)	(285,209, 327,072)	(276,416, 317,513)	(277,068, 318,869)	(280,349, 322,819)	(285,904, 328,854)	(290,866, 334,148)
Other	793,790	743,457	661,908	639,866	657,544	679,427	702,510
	(684,066, 929,221)	(636,512, 875,867)	(560,307, 788,300)	(538,383, 766,029)	(553,797, 786,367)	(573,887, 809,819)	(595,328, 834,192)
**Prevalence rate per 100,000 children (rate, (95% uncertainty interval))**							
COVID-19	0.00	0.00	0.00	0.00	0.00	35.64	114.74
	(0.00, 0.00)	(0.00, 0.00)	(0.00, 0.00)	(0.00, 0.00)	(0.00, 0.00)	(32.73, 38.73)	(107.25, 122.53)
Drug overdose	144.41	190.90	246.50	318.02	435.56	483.05	562.75
	(127.99, 162.18)	(172.39, 210.59)	(225.77, 268.49)	(294.06, 343.14)	(407.09, 465.24)	(452.99, 514.49)	(529.43, 597.58)
Unintentional injuries	471.01	432.79	394.60	369.48	365.54	369.58	393.21
excluding drug overdose	(437.91, 505.61)	(401.15, 465.81)	(364.55, 425.84)	(340.15, 400.08)	(336.21, 396.29)	(340.02, 400.51)	(362.06, 425.74)
Suicide	203.96	195.38	193.77	210.61	233.19	237.83	253.65
excluding drug overdose	(185.27, 223.97)	(177.19, 214.79)	(175.64, 213.20)	(191.21, 231.29)	(212.29, 255.33)	(216.60, 260.22)	(231.15, 277.36)
Homicide	208.24	181.25	156.45	146.61	150.32	155.53	168.93
excluding drug overdose	(186.03, 231.62)	(161.05, 202.81)	(138.03, 176.01)	(128.91, 165.48)	(132.38, 169.39)	(137.29, 174.84)	(149.56, 189.36)
Malignant neoplasms	524.61	505.32	477.08	469.81	458.35	455.34	471.73
	(491.49, 559.47)	(473.15, 539.23)	(446.01, 509.55)	(438.59, 502.42)	(427.10, 490.86)	(424.13, 487.78)	(439.15, 505.55)
Heart disease	419.16	415.68	399.95	403.85	412.07	421.57	449.76
	(390.95, 448.86)	(387.91, 444.85)	(372.92, 428.36)	(376.18, 432.93)	(383.57, 441.68)	(392.61, 451.59)	(419.14, 481.51)
Other	1096.76	1011.18	892.99	868.75	899.65	933.00	1012.33
	(945.15, 1283.88)	(865.72, 1191.27)	(755.92, 1063.51)	(730.96, 1040.04)	(757.71, 1075.91)	(788.07, 1112.05)	(857.88, 1202.09)

Supplementary Table 17: Leading causes of orphanhood among US children from 2000 to 2021.

	**Before the COVID-19 pandemic**					**Since the COVID-19 pandemic**	
	**2000**	**2005**	**2010**	**2015**	**2019**	**2020**	**2021**
**Incidence (n, (95% uncertainty interval))**							
COVID-19	0	0	0	0	0	3,847	4,161
	(0, 0)	(0, 0)	(0, 0)	(0, 0)	(0, 0)	(3,725, 3,971)	(4,033, 4,296)
Drug overdose	199	312	368	492	607	742	838
	(182, 232)	(275, 346)	(325, 399)	(448, 531)	(568, 658)	(693, 785)	(784, 884)
Unintentional injuries	829	895	851	984	983	979	997
excluding drug overdose	(764, 908)	(828, 970)	(789, 921)	(915, 1,043)	(920, 1,038)	(924, 1,039)	(944, 1,057)
Suicide	210	222	243	290	290	265	253
excluding drug overdose	(180, 238)	(190, 254)	(214, 274)	(263, 325)	(254, 317)	(228, 291)	(221, 282)
Homicide	148	146	117	130	124	146	148
excluding drug overdose	(121, 179)	(123, 172)	(109, 144)	(111, 153)	(112, 147)	(126, 176)	(129, 177)
Malignant neoplasms	7,334	7,096	6,974	7,094	6,392	6,054	5,753
	(7,146, 7,534)	(6,911, 7,284)	(6,803, 7,157)	(6,930, 7,265)	(6,249, 6,549)	(5,910, 6,198)	(5,623, 5,896)
Heart disease	9,526	8,376	7,277	7,541	6,991	7,012	6,583
	(9,320, 9,736)	(8,185, 8,571)	(7,116, 7,453)	(7,389, 7,709)	(6,845, 7,147)	(6,864, 7,161)	(6,447, 6,727)
Other	13,329	13,646	13,652	15,274	14,589	14,973	14,007
	(12,386, 14,360)	(12,756, 14,626)	(12,824, 14,590)	(14,423, 16,194)	(13,801, 15,438)	(14,184, 15,799)	(13,240, 14,820)
**Incidence rate per 100,000 children (rate, (95% uncertainty interval))**							
COVID-19	0.00	0.00	0.00	0.00	0.00	5.28	6.00
	(0.00, 0.00)	(0.00, 0.00)	(0.00, 0.00)	(0.00, 0.00)	(0.00, 0.00)	(5.12, 5.45)	(5.81, 6.19)
Drug overdose	0.27	0.42	0.50	0.67	0.83	1.02	1.21
	(0.25, 0.32)	(0.37, 0.47)	(0.44, 0.54)	(0.61, 0.72)	(0.78, 0.90)	(0.95, 1.08)	(1.13, 1.27)
Unintentional injuries	1.15	1.22	1.15	1.34	1.34	1.34	1.44
excluding drug overdose	(1.06, 1.25)	(1.13, 1.32)	(1.06, 1.24)	(1.24, 1.42)	(1.26, 1.42)	(1.27, 1.43)	(1.36, 1.52)
Suicide	0.29	0.30	0.33	0.39	0.40	0.36	0.36
excluding drug overdose	(0.25, 0.33)	(0.26, 0.35)	(0.29, 0.37)	(0.36, 0.44)	(0.35, 0.43)	(0.31, 0.40)	(0.32, 0.41)
Homicide	0.20	0.20	0.16	0.18	0.17	0.20	0.21
excluding drug overdose	(0.17, 0.25)	(0.17, 0.23)	(0.15, 0.19)	(0.15, 0.21)	(0.15, 0.20)	(0.17, 0.24)	(0.19, 0.26)
Malignant neoplasms	10.13	9.65	9.41	9.63	8.75	8.31	8.29
	(9.87, 10.41)	(9.40, 9.91)	(9.18, 9.66)	(9.41, 9.86)	(8.55, 8.96)	(8.12, 8.51)	(8.10, 8.50)
Heart disease	13.16	11.39	9.82	10.24	9.57	9.63	9.49
	(12.88, 13.45)	(11.13, 11.66)	(9.60, 10.06)	(10.03, 10.47)	(9.37, 9.78)	(9.43, 9.83)	(9.29, 9.69)
Other	18.42	18.56	18.42	20.74	19.96	20.56	20.18
	(17.11, 19.84)	(17.35, 19.89)	(17.30, 19.68)	(19.58, 21.99)	(18.88, 21.12)	(19.48, 21.69)	(19.08, 21.36)
**Prevalence (n, (95 uncertainty interval))**							
COVID-19	0	0	0	0	0	3,811	7,519
	(0, 0)	(0, 0)	(0, 0)	(0, 0)	(0, 0)	(3,690, 3,934)	(7,278, 7,757)
Drug overdose	1,409	1,892	2,510	3,193	4,056	4,359	4,729
	(1,236, 1,633)	(1,672, 2,147)	(2,242, 2,785)	(2,912, 3,501)	(3,739, 4,389)	(4,025, 4,697)	(4,377, 5,062)
Unintentional injuries	7,206	7,117	7,111	7,462	7,820	7,873	7,944
excluding drug overdose	(6,509, 7,867)	(6,503, 7,744)	(6,541, 7,720)	(6,906, 8,022)	(7,299, 8,350)	(7,365, 8,399)	(7,442, 8,464)
Suicide	1,969	1,886	1,905	2,129	2,314	2,319	2,311
excluding drug overdose	(1,707, 2,253)	(1,629, 2,139)	(1,692, 2,170)	(1,926, 2,398)	(2,067, 2,583)	(2,066, 2,586)	(2,056, 2,570)
Homicide	1,912	1,514	1,245	1,124	1,104	1,125	1,146
excluding drug overdose	(1,639, 2,219)	(1,282, 1,783)	(1,076, 1,472)	(1,002, 1,350)	(994, 1,309)	(1,008, 1,331)	(1,026, 1,355)
Malignant neoplasms	49,371	47,422	45,943	46,100	44,583	43,728	42,698
	(47,671, 51,053)	(45,844, 48,998)	(44,471, 47,429)	(44,681, 47,524)	(43,220, 45,938)	(42,387, 45,047)	(41,397, 43,998)
Heart disease	67,674	60,984	53,668	50,279	48,398	47,951	47,172
	(65,628, 69,673)	(59,150, 62,805)	(52,037, 55,326)	(48,766, 51,774)	(46,966, 49,810)	(46,541, 49,319)	(45,789, 48,528)
Other	92,505	94,483	95,626	101,446	104,063	104,540	104,005
	(84,523, 101,172)	(86,980, 102,744)	(88,525, 103,516)	(94,490, 109,113)	(97,261, 111,465)	(97,787, 111,922)	(97,374, 111,239)
**Prevalence rate per 100,000 children (rate, (95% uncertainty interval))**							
COVID-19	0.00	0.00	0.00	0.00	0.00	5.23	10.84
	(0.00, 0.00)	(0.00, 0.00)	(0.00, 0.00)	(0.00, 0.00)	(0.00, 0.00)	(5.07, 5.40)	(10.49, 11.18)
Drug overdose	1.95	2.57	3.39	4.34	5.55	5.99	6.81
	(1.71, 2.26)	(2.27, 2.92)	(3.02, 3.76)	(3.95, 4.75)	(5.12, 6.00)	(5.53, 6.45)	(6.31, 7.29)
Unintentional injuries	9.96	9.68	9.59	10.13	10.70	10.81	11.45
excluding drug overdose	(8.99, 10.87)	(8.84, 10.53)	(8.82, 10.42)	(9.38, 10.89)	(9.99, 11.42)	(10.11, 11.53)	(10.72, 12.20)
Suicide	2.72	2.57	2.57	2.89	3.17	3.18	3.33
excluding drug overdose	(2.36, 3.11)	(2.22, 2.91)	(2.28, 2.93)	(2.61, 3.26)	(2.83, 3.53)	(2.84, 3.55)	(2.96, 3.70)
Homicide	2.64	2.06	1.68	1.53	1.51	1.54	1.65
excluding drug overdose	(2.26, 3.07)	(1.74, 2.42)	(1.45, 1.99)	(1.36, 1.83)	(1.36, 1.79)	(1.38, 1.83)	(1.48, 1.95)
Malignant neoplasms	68.22	64.50	61.98	62.59	61.00	60.05	61.53
	(65.87, 70.54)	(62.35, 66.64)	(60.00, 63.99)	(60.66, 64.52)	(59.13, 62.85)	(58.21, 61.86)	(59.65, 63.40)
Heart disease	93.50	82.95	72.40	68.26	66.22	65.85	67.98
	(90.68, 96.26)	(80.45, 85.42)	(70.20, 74.64)	(66.21, 70.29)	(64.26, 68.15)	(63.91, 67.73)	(65.98, 69.93)
Other	127.81	128.51	129.01	137.73	142.38	143.56	149.87
	(116.78, 139.79)	(118.30, 139.74)	(119.43, 139.65)	(128.29, 148.14)	(133.07, 152.51)	(134.28, 153.69)	(140.32, 160.30)

Supplementary Table 18: Leading causes of primary grandparent caregiver loss among US children from 2000 to 2021.

	Before the COVID-19 pandemic					Since the COVID-19 pandemic	
	2000	2005	2010	2015	2019	2020	2021
**Incidence (n, (95% uncertainty interval))**							
COVID-19	0	0	0	0	0	7,265	7,943
	(0, 0)	(0, 0)	(0, 0)	(0, 0)	(0, 0)	(7,037, 7,499)	(7,690, 8,206)
Drug overdose	245	378	441	718	1,004	1,246	1,442
	(213, 280)	(337, 433)	(405, 502)	(647, 797)	(931, 1,083)	(1,159, 1,338)	(1,351, 1,535)
Unintentional injuries	1,073	1,155	1,087	1,468	1,660	1,682	1,760
excluding drug overdose	(980, 1,180)	(1,062, 1,258)	(1,013, 1,186)	(1,365, 1,567)	(1,543, 1,769)	(1,565, 1,789)	(1,649, 1,878)
Suicide	270	293	326	447	490	456	468
excluding drug overdose	(226, 332)	(238, 337)	(287, 370)	(387, 491)	(440, 552)	(400, 505)	(412, 518)
Homicide	183	170	139	176	197	238	255
excluding drug overdose	(149, 240)	(147, 207)	(117, 170)	(141, 201)	(155, 223)	(202, 262)	(225, 296)
Malignant neoplasms	9,324	9,054	8,912	10,605	10,840	10,462	10,174
	(9,059, 9,600)	(8,802, 9,308)	(8,680, 9,157)	(10,332, 10,882)	(10,570, 11,126)	(10,184, 10,729)	(9,917, 10,453)
Heart disease	12,052	10,615	9,209	11,084	11,603	11,889	11,360
	(11,775, 12,331)	(10,359, 10,864)	(8,991, 9,438)	(10,836, 11,342)	(11,347, 11,878)	(11,620, 12,161)	(11,104, 11,638)
Other	16,968	17,403	17,492	22,808	24,601	25,701	24,510
	(15,700, 18,388)	(16,192, 18,757)	(16,360, 18,763)	(21,445, 24,275)	(23,184, 26,164)	(24,241, 27,267)	(23,070, 26,066)
**Incidence rate per 100,000 children (rate, (95% uncertainty interval))**							
COVID-19	0.00	0.00	0.00	0.00	0.00	9.98	11.45
	(0.00, 0.00)	(0.00, 0.00)	(0.00, 0.00)	(0.00, 0.00)	(0.00, 0.00)	(9.66, 10.30)	(11.08, 11.83)
Drug overdose	0.34	0.51	0.59	0.97	1.37	1.71	2.08
	(0.29, 0.39)	(0.46, 0.59)	(0.55, 0.68)	(0.88, 1.08)	(1.27, 1.48)	(1.59, 1.84)	(1.95, 2.21)
Unintentional injuries	1.48	1.57	1.47	1.99	2.27	2.31	2.54
excluding drug overdose	(1.35, 1.63)	(1.44, 1.71)	(1.37, 1.60)	(1.85, 2.13)	(2.11, 2.42)	(2.15, 2.46)	(2.38, 2.71)
Suicide	0.37	0.40	0.44	0.61	0.67	0.63	0.67
excluding drug overdose	(0.31, 0.46)	(0.32, 0.46)	(0.39, 0.50)	(0.53, 0.67)	(0.60, 0.76)	(0.55, 0.69)	(0.59, 0.75)
Homicide	0.25	0.23	0.19	0.24	0.27	0.33	0.37
excluding drug overdose	(0.21, 0.33)	(0.20, 0.28)	(0.16, 0.23)	(0.19, 0.27)	(0.21, 0.31)	(0.28, 0.36)	(0.32, 0.43)
Malignant neoplasms	12.88	12.31	12.02	14.40	14.83	14.37	14.66
	(12.52, 13.26)	(11.97, 12.66)	(11.71, 12.35)	(14.03, 14.77)	(14.46, 15.22)	(13.98, 14.73)	(14.29, 15.06)
Heart disease	16.65	14.44	12.42	15.05	15.88	16.33	16.37
	(16.27, 17.04)	(14.09, 14.78)	(12.13, 12.73)	(14.71, 15.40)	(15.52, 16.25)	(15.96, 16.70)	(16.00, 16.77)
Other	23.44	23.67	23.60	30.97	33.66	35.29	35.32
	(21.69, 25.41)	(22.02, 25.51)	(22.07, 25.31)	(29.12, 32.96)	(31.72, 35.80)	(33.29, 37.44)	(33.24, 37.56)
**Prevalence (n, (95 uncertainty interval))**							
COVID-19	0	0	0	0	0	7,203	14,290
	(0, 0)	(0, 0)	(0, 0)	(0, 0)	(0, 0)	(6,976, 7,434)	(13,810, 14,767)
Drug overdose	1,779	2,331	3,059	4,140	5,869	6,497	7,263
	(1,473, 2,081)	(2,026, 2,679)	(2,744, 3,468)	(3,738, 4,667)	(5,350, 6,458)	(5,950, 7,114)	(6,693, 7,899)
Unintentional injuries	9,213	9,182	9,175	10,067	11,526	11,896	12,305
excluding drug overdose	(8,326, 10,185)	(8,385, 10,054)	(8,455, 9,979)	(9,339, 10,848)	(10,737, 12,371)	(11,078, 12,742)	(11,482, 13,167)
Suicide	2,723	2,544	2,565	2,958	3,498	3,577	3,656
excluding drug overdose	(2,140, 3,155)	(2,045, 3,000)	(2,133, 2,999)	(2,543, 3,401)	(3,066, 3,921)	(3,136, 4,000)	(3,213, 4,080)
Homicide	2,387	1,905	1,548	1,439	1,559	1,623	1,696
excluding drug overdose	(1,951, 2,905)	(1,577, 2,312)	(1,309, 1,855)	(1,187, 1,653)	(1,270, 1,750)	(1,329, 1,815)	(1,412, 1,910)
Malignant neoplasms	62,688	60,544	58,853	62,807	66,985	67,377	67,411
	(60,252, 65,094)	(58,267, 62,789)	(56,748, 60,954)	(60,710, 64,907)	(64,802, 69,157)	(65,158, 69,531)	(65,206, 69,581)
Heart disease	85,283	77,180	67,968	67,236	71,153	72,358	72,888
	(82,349, 88,080)	(74,508, 79,689)	(65,619, 70,213)	(65,023, 69,391)	(68,893, 73,365)	(70,037, 74,557)	(70,571, 75,127)
Other	117,157	120,161	122,118	137,491	155,010	159,728	162,703
	(106,070, 129,450)	(109,643, 131,771)	(112,246, 132,994)	(127,411, 148,557)	(144,392, 166,767)	(148,780, 171,694)	(151,605, 174,895)
**Prevalence rate per 100,000 children (rate, (95% uncertainty interval))**							
COVID-19	0.00	0.00	0.00	0.00	0.00	9.89	20.59
	(0.00, 0.00)	(0.00, 0.00)	(0.00, 0.00)	(0.00, 0.00)	(0.00, 0.00)	(9.58, 10.21)	(19.90, 21.28)
Drug overdose	2.46	3.17	4.13	5.62	8.03	8.92	10.47
	(2.04, 2.87)	(2.76, 3.64)	(3.70, 4.68)	(5.08, 6.34)	(7.32, 8.84)	(8.17, 9.77)	(9.65, 11.38)
Unintentional injuries	12.73	12.49	12.38	13.67	15.77	16.34	17.73
excluding drug overdose	(11.50, 14.07)	(11.40, 13.67)	(11.41, 13.46)	(12.68, 14.73)	(14.69, 16.93)	(15.21, 17.50)	(16.55, 18.97)
Suicide	3.76	3.46	3.46	4.02	4.79	4.91	5.27
excluding drug overdose	(2.96, 4.36)	(2.78, 4.08)	(2.88, 4.05)	(3.45, 4.62)	(4.20, 5.37)	(4.31, 5.49)	(4.63, 5.88)
Homicide	3.30	2.59	2.09	1.95	2.13	2.23	2.44
excluding drug overdose	(2.70, 4.01)	(2.15, 3.14)	(1.77, 2.50)	(1.61, 2.24)	(1.74, 2.39)	(1.83, 2.49)	(2.03, 2.75)
Malignant neoplasms	86.61	82.35	79.40	85.27	91.65	92.52	97.14
	(83.25, 89.94)	(79.25, 85.40)	(76.56, 82.23)	(82.43, 88.12)	(88.66, 94.62)	(89.48, 95.48)	(93.96, 100.27)
Heart disease	117.83	104.97	91.70	91.29	97.35	99.36	105.03
	(113.78, 121.70)	(101.34, 108.38)	(88.53, 94.73)	(88.28, 94.21)	(94.26, 100.38)	(96.18, 102.38)	(101.69, 108.26)
Other	161.87	163.43	164.75	186.67	212.09	219.34	234.46
	(146.55, 178.86)	(149.13, 179.22)	(151.43, 179.42)	(172.99, 201.70)	(197.56, 228.17)	(204.31, 235.77)	(218.47, 252.03)

Supplementary Table 19: Leading causes of secondary grandparent caregiver loss among US children from 2000 to 2021.
