# Supplementary material for: Orphanhood and caregiver death among children in the United States by all-cause mortality, 2000–2021
Source: Nat Med. 2025 Jan 10;31(2):672–83. doi: 10.1038/s41591-024-03343-6 (PMC11835524; doi:10.1038/s41591-024-03343-6)
Supplement: Supplementary file 2 — Reporting Summary [file 41591_2024_3343_MOESM2_ESM.pdf]

nature portfolio

Corresponding author(s): Andres Villaveces, Oliver Ratmann

Last updated by author(s): Jul 31, 2024

Reporting Summary

Nature Portfolio wishes to improve the reproducibility of the work that we publish. This form provides structure for consistency and transparency in reporting. For further information on Nature Portfolio policies, see our Editorial Policies and the Editorial Policy Checklist.

Statistics

For all statistical analyses, confirm that the following items are present in the figure legend, table legend, main text, or Methods section.

n/a	Confirmed	
☐	☒	The exact sample size (*n*) for each experimental group/condition, given as a discrete number and unit of measurement
☐	☒	A statement on whether measurements were taken from distinct samples or whether the same sample was measured repeatedly
☒	☐	The statistical test(s) used AND whether they are one- or two-sided<br>*Only common tests should be described solely by name; describe more complex techniques in the Methods section.*
☐	☒	A description of all covariates tested
☐	☒	A description of any assumptions or corrections, such as tests of normality and adjustment for multiple comparisons
☐	☒	A full description of the statistical parameters including central tendency (e.g. means) or other basic estimates (e.g. regression coefficient) AND variation (e.g. standard deviation) or associated estimates of uncertainty (e.g. confidence intervals)
☒	☐	For null hypothesis testing, the test statistic (e.g. *F*, *t*, *r*) with confidence intervals, effect sizes, degrees of freedom and *P* value noted<br>*Give P values as exact values whenever suitable.*
☒	☐	For Bayesian analysis, information on the choice of priors and Markov chain Monte Carlo settings
☒	☐	For hierarchical and complex designs, identification of the appropriate level for tests and full reporting of outcomes
☒	☐	Estimates of effect sizes (e.g. Cohen's *d*, Pearson's *r*), indicating how they were calculated

Our web collection on statistics for biologists contains articles on many of the points above.

Software and code

Policy information about availability of computer code

Data collection	Publicly available mortality and natality data from NCHS were downloaded with R version 3.5.1. Code to download the data is freely available at https://github.com/MLGlobalHealth/orphanhood-caregiver-death-in-US-from-all-causes-of-mortality.
Data analysis	All data were analyzed using R with version 3.5.1. All code to analyze the data is freely available at https://github.com/MLGlobalHealth/orphanhood-caregiver-death-in-US-from-all-causes-of-mortality. Team communications were supported through the Zulip chat app 5.10.2 (https://zulip.com/).

For manuscripts utilizing custom algorithms or software that are central to the research but not yet described in published literature, software must be made available to editors and reviewers. We strongly encourage code deposition in a community repository (e.g. GitHub). See the Nature Portfolio guidelines for submitting code & software for further information.

Data

Policy information about availability of data

All manuscripts must include a data availability statement. This statement should provide the following information, where applicable:

- Accession codes, unique identifiers, or web links for publicly available datasets
- A description of any restrictions on data availability
- For clinical datasets or third party data, please ensure that the statement adheres to our policy

All data used to calculate estimates of mortality and orphanhood are publicly available, and via the Zenodo repository https://zenodo.org/records/11423745.

Mortality and natality data were sourced from NCHS (https://www.cdc.gov/nchs/data_access/vitalstatsonline.htm). Mortality, natality data after year 2005 at the state level were sourced from CDC WONDER (https://wonder.cdc.gov/). Population data from 1969 to 1989 were sourced from https://seer.cancer.gov/popdata/singleages.html. Population data from 1990 to 2020 were sourced from CDC WONDER (https://wonder.cdc.gov/bridged-race-population.html). Child mortality data were sourced from United Nations (https://population.un.org/wpp/Download/Standard/Mortality/). Household data from 2010 to 2021 were sourced from American Community Survey (https://data.census.gov/table/ACSST5Y2019.S1002).

Research involving human participants, their data, or biological material

Policy information about studies with human participants or human data. See also policy information about sex, gender (identity/presentation), and sexual orientation and race, ethnicity and racism.

Reporting on sex and gender	Sex was reported in primary data sources, and was assigned in birth/death records, and based on self-reported census data for population size estimates and grandparent caregivers.
Reporting on race, ethnicity, or other socially relevant groupings	Race categories and ethnicity categories were provided separately in the publicly available data sources. Individuals of other races and individuals of more than one race were not coded consistently and not included in this study. We defined standardized race & ethnicity categories ('Hispanic', 'Non-Hispanic American Indian or Alaska Native', 'Non-Hispanic Asian or Pacific Islander', 'Non-Hispanic Black' and 'Non-Hispanic White'), see Methods section.
Population characteristics	Publicly available data were available by 1 year age bands, sex, race & ethnicity. Death registrations were additionally stratified by cause-of-death using either ICD-9 or ICD-10 codes.
Recruitment	No participants were recruited. Analyses are based on publicly available vital statistics.
Ethics oversight	n/a. The manuscript was reviewed and cleared by CDC and NCHS.

Note that full information on the approval of the study protocol must also be provided in the manuscript.

Field-specific reporting

Please select the one below that is the best fit for your research. If you are not sure, read the appropriate sections before making your selection.

☐ Life sciences ☒ Behavioural & social sciences ☐ Ecological, evolutionary & environmental sciences

For a reference copy of the document with all sections, see nature.com/documents/nr-reporting-summary-flat.pdf

Behavioural & social sciences study design

All studies must disclose on these points even when the disclosure is negative.

Study description	This is a modeling study (statistical analysis) based on population-based, final, published vital statistics data. Mortality data span from 1983 to 2021, natality data from 1968 to 2021, population size estimates from 1966 to 2021. All data obtained from publicly available websites were of quantitative nature. Age of child, sex of parent/grandparent, race/ethnicity of parent/grandparent, state of residence of parent/grandparent, and cause of parental death were considered in the study design.
Research sample	We used population-based, final, published vital statistics on U.S. residents. Mortality data comprised U.S. residents older than 15 years who died from all causes-of-death (women: up to age 66 years, men: up to age 94 years). Live birth data comprised U.S. residents older than 15 years (women: up to age 49 years, men: up to age 77 years). U.S. residents were stratified by race & ethnicity (individuals of multiple race were excluded), sex, and cause-of-death (for mortality records).
Sampling strategy	Data retrieval was based on population-based, final vital statistics data published by NCHS.
Data collection	No participants were recruited for this study, and no additional data were collected.
Timing	We used NCHS vital statistics annual mortality data from 1983 to 2021, annual natality data from 1968 to 2021 and annual population size data from 1966 to 2021. ACS annual household data were from 2010 to 2021.
Data exclusions	We assumed a maximum childbearing age of 49 years for women and 77 years for men; women of age 50 years mothering a child were excluded, as were men of age 78 years and above fathering a child. Individuals with unreported or not-stated demographic information were excluded, as were individuals of multiple race; as explained in the Extended Methods section.
Non-participation	No participants were recruited for this study, and no additional data were collected.
Randomization	We used population-based, final vital statistics data published by NCHS.

Reporting for specific materials, systems and methods

We require information from authors about some types of materials, experimental systems and methods used in many studies. Here, indicate whether each material, system or method listed is relevant to your study. If you are not sure if a list item applies to your research, read the appropriate section before selecting a response.

Materials & experimental systems

n/a	Involved in the study
☒	☐ Antibodies
☒	☐ Eukaryotic cell lines
☒	☐ Palaeontology and archaeology
☒	☐ Animals and other organisms
☒	☐ Clinical data
☒	☐ Dual use research of concern
☒	☐ Plants

Methods

n/a	Involved in the study
☒	☐ ChIP-seq
☒	☐ Flow cytometry
☒	☐ MRI-based neuroimaging

Plants

Seed stocks	n/a
Novel plant genotypes	n/a
Authentication	n/a
