# Supplementary material for: Orphanhood and caregiver death among children in the United States by all-cause mortality, 2000–2021
Source: Nat Med. Author manuscript; Available in PMC 2025 Feb 19. (PMC11835524; doi:10.1038/s41591-024-03343-6)
Supplement: Supplementary file 1 [file NIHMS2048424-supplement-Published_research_brief.pdf]

Check for updates

Visualizing the effects and causes of caregiver deaths on children in the USA

Death of caregivers threaten child wellbeing. We estimate that 2.91 million children and adolescents in the USA had experienced death of a caregiver between 2000 and 2021. We describe the drivers of orphanhood and identify populations that are disproportionately affected. Our findings may inform policies and programs that address caregiver loss and its consequences.

The problem

Across the globe, the lives of children are increasingly affected by intersecting crises, including infectious diseases, exposures to violence, conflict that leads to migration, and climate change. These crises also affect the caregivers who are responsible for most of the needs of their children or grandchildren. At present, available data on caregiver loss are limited to specific causes such as HIV-related infections, COVID-19, maternal cancers, or drug overdoses[1–4]. We estimated the magnitude of orphanhood (death of one or both parents) and caregiver loss (including co-residing grandparent caregivers) in the USA due to all causes and due to leading causes of death. Understanding the magnitude of caregiver loss due to both all causes and leading causes, including incidence, prevalence trends and associated inequalities, is essential for informing evidence-based prevention and response strategies for affected children and families.

The observation

We estimated the incidence and prevalence of all-cause orphanhood, as well as loss of co-residing grandparent caregivers, among children in the USA. This was achieved by leveraging modeling methodologies previously used to estimate COVID-19 associated orphanhood and caregiver death[5]. We combined multiple datasets that included state-level population and mortality estimates by sex, age, race and ethnicity, survey estimates of family composition to determine numbers of grandparent caregivers in homes, and fertility rates from 1969 to 2021 to estimate the number of children in families by age categories. This allowed us to determine the incidence and prevalence of children affected by death of a caregiver or a co-residing grandparent caregiver each year.

By 2021, we estimate that 2.91 million children (4.2% of all children in the USA) had experienced prevalent orphanhood and caregiver death, with the incidence (number of new cases in the population per unit of time) increasing by 49.5% between 2000 and 2021, and the prevalence (total number of cases in the population per unit of time) increasing by 7.9%. Most children lost a parent, and nearly 20% lost a grandparent caregiver who provided housing and some needs. The five states with the highest orphanhood burden were also the five states with highest poverty levels (Fig. 1).

Since 2020, drug overdose has been the leading cause of orphanhood incidence and prevalence, surpassing COVID-19. The incidence and prevalence of orphanhood due to fatal injuries (drug overdose, suicide, homicide and unintentional injuries) exceeded orphanhood due to leading chronic disease (such as heart disease and cancers) causes of death in parents as of 2021. Children were more likely to experience the death of a father than a mother. One of every twenty children aged 10–17 years in the USA experienced orphanhood. In general, children of non-Hispanic American Indian or Indigenous Alaska heritage had the greatest orphanhood burden, followed by non-Hispanic Black people, and non-Hispanic white people. However, burdens varied by cause and state.

The implications

Effective policies and programs that support nearly 3 million bereaved children are needed to reduce the negative effects of orphanhood in the USA. This requires approaches contextualized to the disparities affecting subgroups, geographies and orphanhood causes. Respective investments in social determinants of health may range from tackling drug overdose to addressing disparities of minoritized populations, and prioritizing support in states with the greatest poverty.

Our findings across the USA shed light on the broader global relevance of this urgent public health imperative, given emerging data on escalating all-cause orphanhood in sub-Saharan Africa and Latin America. Leveraging lessons from two decades of evidence-based care for African children who are orphans due to AIDS can inform urgent action.

Our modelling data of orphanhood are likely underestimations due to mortality undercounts. Estimations of fertility may over or undercount the number of children and hence number of estimated orphans. In addition, limited information on the roles of grandparents supporting children may underestimate their contributions to caregiving.

Policies that address needs shared across orphanhood causes and health disparities are a public health and moral imperative. Strategies that focus on preventing death of caregivers by accelerating equitable access to health and social services, preparing families and caregivers to provide safe and nurturing family-based support, and protecting children using evidence-based strategies that address their childhood adversities and strengthen their recovery are needed.

Andrés Villaveces[1] & Susan Hillis[2]
[1]US Centers for Disease Control and Prevention, Atlanta, GA, USA.
[2]Imperial College, London, UK.

This is a summary of:
Villaveces, A. et al. Orphanhood and caregiver death among children in the United States by all-cause mortality, 2000–2021. *Nat. Med.* https://doi.org/10.1038/s41591-024-03343-6 (2025).

Publisher's note
Springer Nature remains neutral with regard to jurisdictional claims in published maps and institutional affiliations.

Published online: 10 January 2025

EXPERT OPINION

“This is a strong study that presents important, detailed information that could allow governments to push for and target additional services. I think the work will generate a lot of vital attention, particularly as the study found that drug overdoses exceed COVID-19 as a cause of orphanhood in 2020–2021. This is a public health challenge that requires far more prevention and mitigation services.” **Rachel Kidman, Stony Brook University, Stony Brook, NY, USA.**

FIGURE

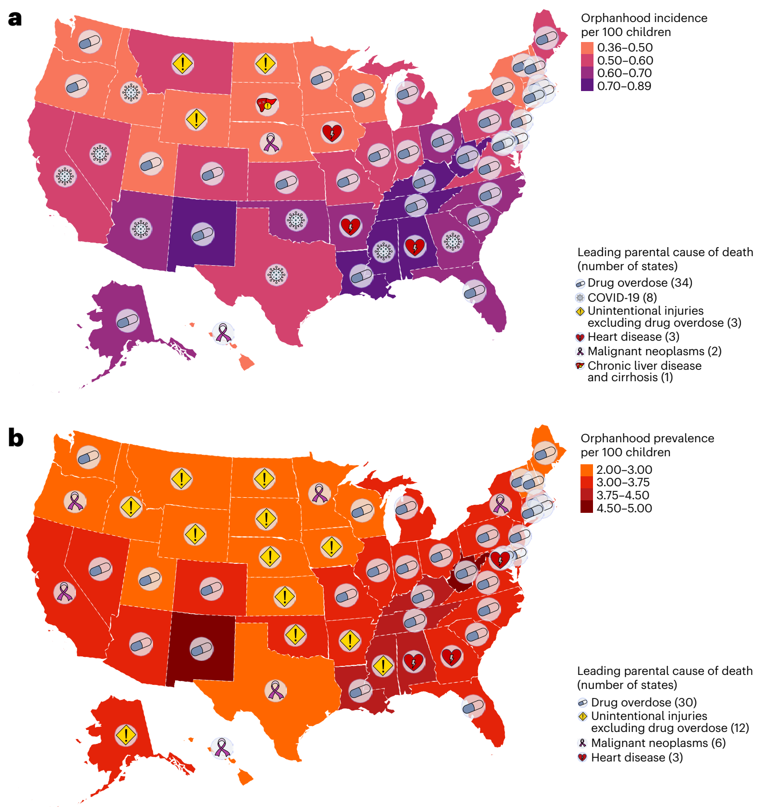

Fig. 1 | Spatial distribution of children experiencing orphanhood in 2021 in the USA. **a**, Map of orphanhood incidence rates per 100 children (color) by state and leading cause (symbol). **b**, Map of orphanhood prevalence rates per 100 children (color) by state and leading cause (symbol). © 2024, Basemaps from Mapbox and OpenStreetMap were generated by ‘Leaflet’ package version 2.2.0 in R under a Creative Commons license CC BY-SA 2.0.

REFERENCES

1. Guida, F. et al. Global and regional estimates of orphans attributed to maternal cancer mortality in 2020. *Nat. Med.* **28**, 2563–2572 (2022).
A research article estimating orphanhood due to maternal cancer.
2. Hillis, S. D. et al. COVID-19-associated orphanhood and caregiver death in the United States. *Pediatrics* **148**, e2021053760 (2021).
Our previous manuscript estimating orphanhood due to COVID-19 associated caregiver deaths in the USA.
3. Jones, C. M. et al. Estimated number of children who lost a parent to drug overdose in the US from 2011 to 2021. *JAMA Psychiatry* **81**, 789–796 (2024).
A manuscript that presents estimations of orphanhood due to parent overdose-associated deaths.
4. Schluter, B. S., Alburez-Gutierrez, D., Bibbins-Domingo, K., Alexander, M. J. & Kiang, M. V. Youth experiencing parental death due to drug poisoning and firearm violence in the US, 1999–2020. *JAMA* **331**, 1741–1747 (2024).
A manuscript that presents estimations of orphanhood due to parental overdose and firearm violence-associated deaths.
5. Hillis, S. D. et al. Global minimum estimates of children affected by COVID-19-associated orphanhood and deaths of caregivers: a modelling study. *Lancet* **398**, 391–402 (2021).
Our previous manuscript estimating global COVID-19-associated orphanhood due to death of caregivers.

BEHIND THE PAPER

The Global Reference Group for Children Affected by Crisis comprises a small interdisciplinary network of global experts who came together during the COVID-19 pandemic to produce global estimates of pandemic-related orphanhood and link these estimates to a unified strategy for evidence-based action. Now, given the escalating compound crises threatening the wellbeing of children globally, the Global Reference Group for Children Affected by Crisis aims to link science to evidence-based action and advocacy across poly-crises of contagion, conflict and climate. Consequently, documenting orphanhood associated with all-cause mortality among caregivers was a necessary step. Our biggest challenges were related to availability and correct interpretation of data, but we closely collaborated with the US National Center for Health Statistics to address these difficulties. Analyzing these data where available is key to better document the magnitude and nature of this problem and implement more targeted public health measures. **A.V. & S.H.**

FROM THE EDITOR

“Orphanhood is a significant yet overlooked public health crisis that endangers children’s health, wellbeing, and social and financial security. This study highlights the prevalence and causes of orphanhood and caregiver loss in the USA, revealing the burden created by multiple crises from the opioid epidemic, gun violence, institutional racism and economic instability. These factors have lasting effects across generations that also intensify social disparities.” **Editorial Team, *Nature Medicine*.**
